# Accuracy of colposcopy to triage HPV-positive women in cervical cancer screening: a systematic review and meta-analysis

**DOI:** 10.1016/j.eclinm.2026.104009

**Published:** 2026-06-17

**Authors:** Nina Dhollander, Iman Jaafar, Maribel Almonte, Remila Rezhake, Laura Downham, Joan Valls, Arianis Tatiana Ramirez, Armando Baena, Lynne Gaffikin, Paul D. Blumenthal, Siri Hovland, Murat Gultekin, Fanghui Zhao, Rengaswamy Sankaranarayanan, Partha Basu, Le Dang, Youlin Qiao, Marc Arbyn

**Affiliations:** a Unit Cancer Epidemiology, Belgian Cancer Centre, Sciensano, Brussels, Belgium; b Department of Non-Communicable Diseases, World Health Organisation, Geneva, Switzerland; c International Agency for Research on Cancer, Lyon, France; d Affiliated Cancer Hospital of Xinjiang Medical University, Urumqi, China; e Department of Nursing and Physiotherapy, University of Lleida, Lleida, Spain; f Biomedical Research Institute of Lleida (IRBLleida), Lleida, Spain; g Division of Cancer Epidemiology and Genetics, National Cancer Institute, Bethesda, MD, USA; h Department of Obstetrics and Gynecology, Stanford University, Stanford, CA, USA; i Center for Innovation in Global Health, Stanford University, Stanford, CA, USA; j PreTect AS, Klokkarstua, Norway; k European Society of Gynaecological Oncology (ESGO), European Network of Gynaecological Cancers Advocacy Groups (ENGAGe) Executive Group, Brussels, Belgium; l Department of Cancer Epidemiology, National Cancer Center/National Clinical Research Center for Cancer/Cancer Hospital, Chinese Academy of Medical Sciences & Peking Union Medical College, Beijing, China; m Karkinos Healthcare, Kerala Operations, Ernakulam, India; n Department of Obstetrics and Gynecology, Peking Union Medical College Hospital, Chinese Academy of Medical Sciences & Peking Union Medical College, Beijing, China; o School of Population Medicine and Public Health, Chinese Academy of Medical Sciences & Peking Union Medical College, Beijing, China; p Department of Human Structure and Repair, Faculty of Medicine and Health Sciences, University Ghent, Ghent, Belgium

**Keywords:** Cervical cancer screening, Triage HPV-Positive women, Colposcopy, Cervical cytology, Systematic review, Diagnostic test accuracy

## Abstract

**Background:**

The World Health Organization (WHO) guidelines on cervical cancer screening recommends colposcopy as one of the methods to triage human papillomavirus (HPV)-positive women. We aimed to assess the diagnostic accuracy of colposcopy in this context.

**Methods:**

In this systematic review and meta-analysis, we searched for articles reporting the diagnostic accuracy of colposcopy in women with a positive HPV screening test, published in PubMed, Embase or the Cochrane library up to Oct 20, 2025. We included cross-sectional or longitudinal studies if the number of true and false positive and negatives could be derived or obtained after contacting the authors. Studies were excluded if the study population was not representative of a screening population. We collected data on colposcopy triage (index) together with data on cytology triage (comparator), if available, and assessed risk of bias using an adapted Quality Assessment of Diagnostic Accuracy Studies (QUADAS)-2 checklist. The pooled sensitivity and specificity to detect cervical intraepithelial neoplasia grade 2 or worse (CIN2+) and grade 3 or worse (CIN3+) were calculated, as well as the relative sensitivity and specificity of colposcopy triage compared to cytology triage, using a bivariate logistic random effects model. Heterogeneity was measured using τ^2^ and I^2^. Sample size effects were assessed by Deeks’ funnel plots and regression test. Certainty of evidence was assessed using the GRADE tool. This study was registered in PROSPERO, CRD42023389772.

**Findings:**

The literature search identified 5774 records. After screening, we included 11 studies (13,311 participants) in the review, but restricted the primary analysis to 8 studies (10,632 participants) that had complete follow-up after colposcopy triage. The pooled sensitivity for CIN2+ and CIN3+ using colposcopy (low-grade colposcopic impression) were 84.4% (95% CI: 79.0%–88.6%) and 86.9% (95% CI: 81.7–90.8%), respectively. The pooled specificity for < CIN2 was 64.3% (95% CI: 57.4%–70.7%). While heterogeneity between studies was high, with I^2^ up to 95.5%, most studies reported a similar or higher sensitivity and lower specificity compared to cytology (at the lowest cut-off): the pooled relative sensitivity for CIN2+ and CIN3+ were 1.74 (95% CI: 1.53–1.97) and 1.58 (95% CI: 1.35–1.85). The relative specificity for < CIN2 was 0.67 (95% CI 0.61–0.72). Certainty of evidence was considered low to very low due to subjectivity in the assessment of colposcopy, cytology and the reference standard, as well as the high inter-study heterogeneity and the risk of reference standard misclassification.

**Interpretation:**

Studies consistently reported good sensitivity but low specificity of colposcopy to triage women with a positive HPV screening test, showing colposcopy may be an acceptable triage test. While our findings are consistent with current WHO guidelines, low certainty of evidence and risk of over-treatment should be carefully considered.

**Funding:**

World Health Organization; Horizon 2020 Framework Programme for Research and Innovation of the European Commission the European Joint Action EUCanScreen and the European Commission Initiative on Cervical Cancer.

**Research in context**

**Evidence before this study**

Four meta-analyses have been identified in the framework of the preparation of the IARC Handbook on Cervical Screening (IARC handbooks on Cervical Cancer Screening, Volume 18, 2022) that summarized the diagnostic accuracy as a diagnostic method. The Supplementary Material of this Handbook contained a summary of meta-analyses including six triage tests or combination of triage tests useable to manage human papillomavirus (HPV)-positive women. One of them included a meta-analysis of the absolute accuracy of cytology at cut-off Atypical Squamous Cells of Undetermined Significance (ASC-US) + to detect cervical precancer among HPV-positive women but none involved triage using colposcopy. The web annex A of the WHO guideline for screening and treatment of cervical pre-cancer lesions for cervical cancer prevention (2021)^1^ included results of a rapid systematic review on the absolute accuracy of colposcopy among women who tested positive on an HPV DNA screening test to detect cervical intraepithelial neoplasia grade 2 or worse (CIN2+) and CIN3+, which included seven reports from three studies. The WHO concluded that colposcopy is one of the acceptable triage methods to identify women who need immediate treatment, besides cytology and visual inspection after application of acetic acid (VIA). This rapid review was not submitted for publication in a peer-reviewed journal. Our updated literature search in PubMed, Embase and the Cochrane library until October 2025 included a combination of terms related to “cervical cancer”, “HPV”, “screening”, “triage” and “diagnostic accuracy”. We did not identify published papers containing a systematic review on the performance of colposcopy in the context of triage of women who tested positive for HPV during primary cervical cancer screening.

**Added value of this study**

We have conducted a systematic review and meta-analysis with the primary objective of evaluating the diagnostic accuracy of colposcopy for CIN2+ and CIN3+, specifically in the particular context of triaging women who tested positive for HPV during primary cervical cancer screening. The current review was more comprehensive and up-to-date and comprised 22 reports from 11 studies published between Jan 1, 1999, and Oct 20, 2025. We reported on study characteristics, risk of bias and certainty of evidence. Our meta-analysis provides diagnostic test accuracy results for both CIN2+ and CIN3+ and compares the diagnostic test accuracy of colposcopy to cytology at different cut-offs. We found that colposcopy was generally reported more sensitive but less specific than cytology. Our review includes an extensive evaluation of the quality and design of included studies, their consistency, heterogeneity and precision of included studies and we were able to establish the level of evidence.

**Implications of all the available evidence**

Studies consistently reported better sensitivity for CIN2+/CIN3+ but lower specificity when using low-grade colposcopic impression compared to cytology at ASC-US to triage HPV-positive women. These results indicate that colposcopy triage may be an acceptable triage approach in settings where recall of women after a positive screening test is difficult yielding lost-to-follow, but accepting the risk of harms by over-treatment. However, substantial and significant heterogeneity between studies is revealed and therefor the level of evidence should be considered as low.

## Introduction

Cervical cancer is a leading cause of cancer death, with over 660,000 new cases and 350,000 deaths globally in 2022.^1^^,^^2^ Around 90% of all these deaths occur in low- and middle-income countries.^3^ The World Health Organization (WHO) Cervical Cancer Elimination Initiative lists cervical cancer screening as a public health priority. The current WHO guideline recommends women and people who have a cervix (hereinafter referred to as ‘women’) are screened using human papillomavirus (HPV) DNA detection for primary screening followed by treatment of HPV-positive women (screen-and-treat approach) or by triage of HPV-positive women to determine who needs treatment (screen, triage and treat approach). Suggested triage tests are cytology, dual-stain cytology, partial genotyping, visual inspection with acetic acid (VIA), or colposcopy.^1^ The preferred triage approach depends not only on test performance, but also on cost, feasibility, training and other available resources.

In the case of triage using colposcopy, the physician decides on the best clinical action based on the colposcopic impression, immediately providing feedback and starting appropriate treatment if needed. This is in contrast with disease confirmation using colposcopy, where the physician takes a biopsy and waits for the histology result before deciding on clinical action. Since one of the main factors for screening failure in low- and middle-income countries is poor follow-up, triage using colposcopy could bridge the gap between screening and further management and help achieve the WHO’s targets of 90% of women with a positive screening test or a cervical lesion receiving appropriate management.^1^

A rapid review to prepare WHO guidelines on the management of HPV-positive women, including only a limited number of studies on triage by colposcopy, published before 2011, was extended to cover information up to 2025.

To our knowledge there is no additional systematic review on this topic. Therefore, we have conducted this systematic review and meta-analysis with the primary objective of evaluating the diagnostic accuracy of colposcopy to triage women who tested positive for HPV during primary cervical cancer screening. The secondary objective was to compare the diagnostic accuracy of colposcopy with cytology in this context.

## Methods

### Study design and ethics

We conducted the systematic review and meta-analysis in accordance with the Preferred Reporting Items for Systematic Reviews and Meta-analyses (PRISMA) guidelines^4^ and the Cochrane Collaboration’s recommendations for systematic reviews of diagnostic test accuracy.^5^ We registered the protocol with PROSPERO (registry number CRD42023389772). Given this study design and the exclusive use of published material, no formal ethics approval or written informed consent was sought.

### Search strategy and selection criteria

We defined the selection criteria using the Population, Intervention, Comparator, Outcome and Study components (PICOS; Supplementary Materials, page 2). Studies were eligible if they met the following criteria: original diagnostic accuracy data on colposcopy triage for women with a positive HPV DNA screening test was reported or computable or provided by the authors upon request, the disease of interest was cervical (pre-)cancer, defined as cervical intraepithelial neoplasia of grade 2 or 3 or worse (CIN2+/CIN3+). Both observational and randomised studies with a cross-sectional design (i.e., colposcopy triage and biopsy were performed at the same time) or a longitudinal design (i.e., the reference standard was performed a certain time after an initial negative triage) were eligible for inclusion. We restricted the review to conventional colposcopy, excluding results from portable colposcopy devices and colposcopy using artificial intelligence, spectroscopy or other adjunctive tools, which will be the object of another systematic review.

We searched PubMed, Embase and the Cochrane library without restrictions on language or publication year until 20 October 2025. We used a combination of terms related to “cervical cancer”, “HPV” and “colposcopy” (Supplementary Materials, page 2). Titles and abstracts were screened by two independent reviewers (ND, RR, IJ, LD and/or ATR). Full texts were retrieved and re-examined for their eligibility (ND, RR, IJ, LD and/or ATR). We manually searched through the PubMed ‘similar articles’ and ‘cited by’ features of the included studies, as well as their Scopus (www.scopus.com) citations, and screened these records in the same way. Any discordances were resolved through discussion among reviewers or, if no consensus could be reached, through discussion with a senior reviewer (MAr).

### Data analysis

For each included study the number of true positives (TP), false negatives (FN), false positives (FP) and true negatives (TN) after triage with colposcopy, and after triage with cytology if available, were extracted by two independent reviewers (ND, RR, IJ and/or LD) using a standardised electronic data entry form. Colposcopy data was extracted using two cut-offs: low-grade impression (includes results reported as abnormal colposcopy, low-grade impression, minor or grade 1 impression, LSIL (low-grade squamous intraepithelial lesion), or worse) and high-grade impression (includes results reported as high-grade impression, positive major or grade 2 impression, HSIL (high-grade squamous intraepithelial lesion), glandular atypia, malignancy, cancer). Cytology data was extracted using three cut-offs: ASC-US (atypical squamous cells of undetermined significance), LSIL and HSIL. We used the following reference standard in order of preference: (1) histological assessment (worst result at screening or follow-up visit); (2) normal colposcopy and normal follow-up test; (3) normal colposcopy and no follow-up test. Note that (2) and (3) can only serve as a reference standard for those free of disease and studies with (3) were considered at high risk of bias due to incomplete follow-up. Women with abnormal colposcopy and no histological result (e.g., the histological result was unsatisfactory) were excluded from the meta-analysis. If we were not able to retrieve or compute diagnostic accuracy data from the published text, we systematically contacted authors to obtain the information. Study characteristics were retrieved and compiled in a comprehensive table. The quality of the included studies was assessed using version-2 of the Quality Assessment of Diagnostic Accuracy Studies (QUADAS-2) checklist (Supplementary Materials, page 12).^6^ Description of study characteristics and QUADAS-2 assessment was done by two independent reviewers (ND, RR). Certainty of evidence was assessed by three authors (TAR, LD, MAr) using the GRADE principles (https://book.gradepro.org/guideline/overview-of-the-grade-approach).^7^^,^^8^ Conflicts were resolved by discussion and if necessary submitted to a senior reviewer (MAr).

The pooled absolute sensitivity and specificity were estimated jointly by fitting a bivariate logistic random effects model using *metadta*,^9^ a statistical procedure in Stata for meta-analysis of diagnostic test accuracy data that takes the intrinsic correlation between sensitivity and specificity into account. A similar analysis was done to pool the relative sensitivity and specificity of colposcopy triage versus cytology triage. Separate meta-analyses were conducted for each cut-off and for each outcome (CIN2+/CIN3+). Analyses were stratified by completeness of follow-up (complete/incomplete). Heterogeneity among studies was measured using τ^2^ (variability due to heterogeneity) and I^2^ (percentage variability due to inter-study heterogeneity).^10^^,^^11^ A leave-one-out sensitivity analysis was performed to assess the influence of individual studies on the pooled estimates.

Sample size effects, including publication bias, were assessed by Deeks’ funnel plots and by Deeks’ regression test.^12^ Statistical significance was defined as p < 0.05 for two sided tests. The pooled estimates for sensitivity and specificity based on studies with complete follow-up were used to calculate predictive values at different levels of pre-triage risk of CIN2+ and CIN3+. Publications reporting accuracy estimates for multiple countries or sites were considered as separate reports in the analysis. All data were analysed using STATA/SE 16.1 (STATA Corp, College Station, TX, USA) software.

### Role of the funding source

The funders of the study had no role in study design, data collection, data analysis, data interpretation, or writing of the report.

## Results

**Fig. 1:**
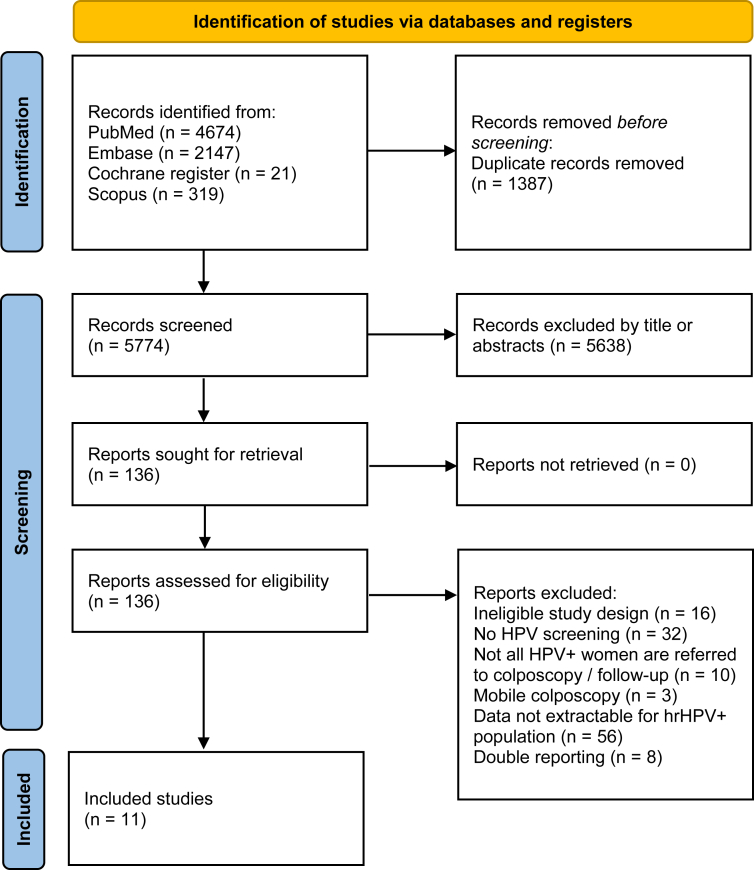
**PRISMA flow diagram of the study selection**.

The PRISMA flow diagram showing the selection of eligible studies can be found in Fig. 1. The search yielded 7161 records of which 5774 were unique. 5638 records were excluded after title and abstract screening, 136 records were screened in full text. The most common reason for exclusion when assessing eligibility in full text was that accuracy data for triage with colposcopy in women with a positive HPV screening test could not be extracted from the publication or by contacting the authors (56 studies). Eleven studies met the selection criteria and were included in the review after extracting data from the publication (4 studies^13^, ^14^, ^15^, ^16^) or after receiving data from the authors (7 studies^17^, ^18^, ^19^, ^20^, ^21^, ^22^, ^23^) (Table 1).

**Table 1:** Summary of study characteristics of the included studies.

Reference	Region	Year	N	Age range	Colposcopy thresholds	Cytology thresholds	Outcomes	Follow-up	Risk of bias
University of Zimbabwe/JHPIEGO, 1999^17^	Zimbabwe	1995–1997	917	25–55	Low-grade	ASC-US, LSIL, HSIL	CIN2+	Incomplete	High
Belinson, 2001^18^	China	1999	363	35–45	Low-grade	ASC-US, LSIL, HSIL	CIN2+, CIN3+	Complete	Low
Belinson, 2003^19^	China	2001	2074	27–56	Low-grade	No cytology	CIN2+, CIN3+	Complete	Low
Sankaranarayanan, 2004^20^	India	1999–2003	1300	25–65	Low-grade	ASC-US, LSIL, HSIL	CIN2+, CIN3+	Incomplete	High
Hovland, 2010^21^	Democratic Republic of Congo	2003	65	25–60	Low- and high-grade	ASC-US, LSIL, HSIL	CIN2+, CIN3+	Complete	Low
Poli, 2018^14^	India	2010–2013	273	30–49	Low-grade	ASC-US	CIN2+, CIN3+	Complete	Low
Luckett, 2019^13^	Botswana	2018	82	≥25	Low- and high-grade	ASC-US, HSIL	CIN2+	Complete	Low
Dang, 2022^22^	China	2015–2018	843	35–64	Low-grade	ASC-US	CIN2+, CIN3+	Complete	Low
Luckett, 2023^15^	Botswana	2021–2022	1264	25–77	Low- and high-grade	No cytology	CIN2+, CIN3+	Complete	Low
Valls, 2023^23^	Latin-America	2013–2022	5849	30–64	Low- and high-grade	ASC-US, LSIL, HSIL	CIN2+, CIN3+	Complete	Low
Tan, 2023^16^	Australia	2018–2020	462	50–74	Low- and high-grade	ASC-US, LSIL, HSIL	CIN2+	Incomplete	High

N = number of study participants with diagnostic accuracy data for colposcopy and/or cytology; ASC-US = atypical squamous cells of undetermined significance; LSIL = low-grade squamous intraepithelial lesion; HSIL = high-grade squamous intraepithelial lesion; CIN2+/CIN3+ = cervical intraepithelial neoplasia of grade 2 or 3 or worse.

Among the 11 included studies, the overall risk of bias was considered low to moderate for 8 studies (73%)^13^, ^14^, ^15^^,^^18^^,^^19^^,^^21^, ^22^, ^23^ and high for 3 studies (27%).^16^^,^^17^^,^^20^ The risk of bias due to participant selection was considered low in all studies. Enrolled participants were women who were invited to attend screening (n = 5)^14^^,^^18^, ^19^, ^20^^,^^23^ or attended primary care centres (n = 2)^15^^,^^19^ or secondary care centres (n = 2)^17^^,^^21^ or a combination of these methods (n = 1).^15^ One study was embedded in a local screening program.^22^ Studies generally included women who were ≥25 years old, although one study was limited to women older than 50.^16^ All studies excluded women who were pregnant at enrolment or who had a history of hysterectomy or cervical cancer. Three studies excluded women who were not sexually active or unmarried.^14^^,^^22^^,^^23^ Three studies excluded women who were suffering from (gynaecological) symptoms.^13^^,^^20^^,^^21^ One study only enrolled women living with HIV (WLHIV),^13^ one study enrolled a cohort enriched for WLHIV^15^ and one study reported high HIV prevalence.^21^ The other studies did not report on HIV prevalence.

Clinically validated HPV assays^24^ were used as screening test in nine studies: HC2 (Qiagen, Gaithersburg, MD, USA),^17^, ^18^, ^19^, ^20^^,^^23^ GP5+/9+ PCR-EIA,^21^ Xpert HPV (Cepheid, Sunnyvale, CA, USA),^13^ Cobas-4800 (Roche Molecular System, Pleasanton, CF, USA)^23^ or another not otherwise specified validated assay.^16^ The remaining three studies used non-clinically validated HPV assays: *care*HPV (Qiagen, Gaithersburg, MD, USA)^14^^,^^22^ or Ampfire (Atila BioSystems, Mountain View, CA, USA).^15^

Colposcopy (index test) was performed by various professionals: medical officer (n = 1),^14^ colposcopist (n = 1),^23^ physician (n = 2),^21^^,^^22^ gynaecologist (n = 4)^13^^,^^15^^,^^18^^,^^19^ or a combination of these (n = 2).^17^^,^^20^ One study did not specify the profession of the colposcopy performer.^16^ Three studies reported that colposcopists received training in preparation of the study.^1^^,^^16^^,^^20^^,^^23^

Nine out of eleven studies included cytology (comparator test). Five studies used conventional cytology,^13^^,^^14^^,^^17^^,^^20^^,^^23^ three studies used liquid-based cytology^16^^,^^18^^,^^22^ and one study used both on all participants.^21^ Cytology was performed by cytotechnicians (n = 2)^17^^,^^21^ or pathologists (n = 4).^13^^,^^14^^,^^18^^,^^22^ Three studies did not specify the profession of the cytologist.^16^^,^^20^^,^^23^ One study reported that cytologists received training in preparation of the study.^17^

Studies often did not report on test blinding or reported it inconsistently. We were not able to evaluate the risk of bias due to test blinding in five studies.^14^^,^^16^^,^^18^, ^19^, ^20^ Five studies reported that cytologists and colposcopists were blinded to other test results,^13^^,^^15^^,^^17^^,^^21^^,^^22^ while only three studies reported that histopathologists were blinded to other tests.^18^^,^^19^^,^^21^

In all studies an abnormal colposcopic impression was followed up with at least a colposcopy-guided biopsy or excisional treatment, and histology was available as a reference test. Additionally, in 2 studies^18^^,^^19^ a biopsy was taken from each quadrant regardless of whether that quadrant had visible lesions, and in 3 studies^16^^,^^21^^,^^23^ an endocervical curettage (ECC) could be performed. Nevertheless, disease verification always depended to some extent on the colposcopist’s ability to correctly identify and target potential lesions.

The follow-up and reference test after normal colposcopic impression varied between studies. Three studies had incomplete follow-up: women with normal colposcopy did not have a biopsy or a follow-up visit (n = 2)^17^^,^^20^ or some but not all women with normal colposcopy had a biopsy or a follow-up visit (n = 1).^16^ For the purpose of this review women were considered free of disease if no biopsy was taken because of normal colposcopy and if there was no clinical suspicion of cervical lesions. Therefore, these three studies were judged to have a high risk of bias due to differential verification (i.e., women have a different reference test depending on their colposcopy result) and incorporation bias (i.e., the colposcopy result influenced the judgement on absence of CIN2+/CIN3+). The remaining eight studies had complete follow-up and were judged to have low to moderate risk of bias: all HPV-positive women underwent a biopsy regardless of colposcopy result (n = 6)^13^, ^14^, ^15^^,^^18^^,^^19^^,^^21^ or women without disease (<CIN2) at screening had a follow-up visit within 18–24 months (n = 2).^22^^,^^23^ Follow-up visits included either screening with HPV, cytology and VIA or visual inspection with Lugol’s iodine (VILI)^22^ or screening with HPV alone.^23^ Women with a positive result at follow-up were referred to colposcopy.

Detailed study characteristics and risk of bias assessment are reported in the Supplementary Materials (pages 4–13).

**Fig. 2:**
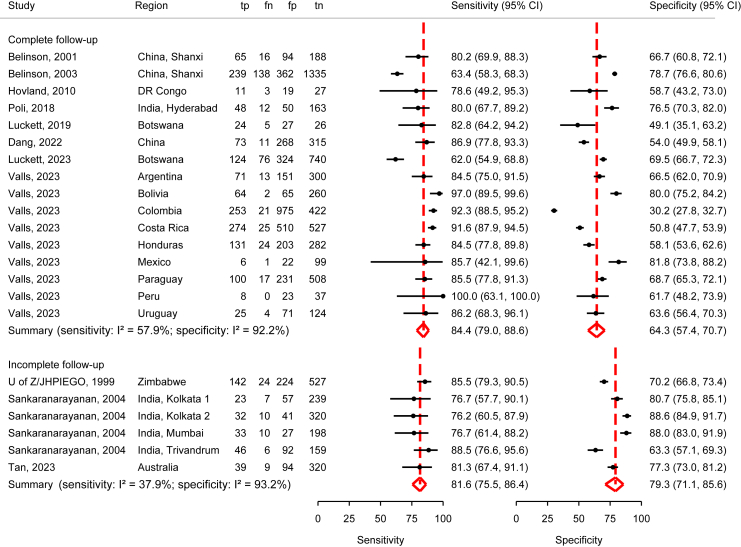
**Forest plot of the diagnostic accuracy of colposcopy triage.** Meta-analysis of the sensitivity (left) and specificity (right) for detection of CIN2+ in the triage of HPV-positive women using colposcopy with low-grade colposcopic impression as test cut-off. Top: studies with complete follow-up; Bottom: studies with incomplete follow-up. Abbreviations: tp = true positives; fn = false negatives; fp = false positives; tn = true negatives; CI = confidence interval; U of Z = University of Zimbabwe; CIN2+ = cervical intraepithelial neoplasia of grade 2 or worse.

The meta-analysis on diagnostic accuracy to detect CIN2+ using colposcopy with low-grade colposcopic impression as test cut-off included 22 reports from 11 studies (Fig. 2). The sensitivity ranged from 62 to 100% and the specificity from 30 to 89%. Using only the studies with complete follow-up (16 reports from 8 studies), the pooled sensitivity and specificity were 84.4% (95% CI: 79.0–88.6%) and 64.3% (95% CI: 57.4–70.7%), respectively. The observed heterogeneity was high: I^2^ was 57.9% for sensitivity and 92.2% for specificity. Studies with incomplete follow-up (6 reports from 3 studies) showed a similar pooled sensitivity (81.6%, 95% CI: 75.5–86.4%), but higher pooled specificity (79.3%, 95% CI: 71.2–85.6%) than studies with complete follow-up. More detailed results are available in Table 2 and the Supplementary Materials (page 14–15). In general, using high-grade instead of low-grade colposcopic impression as a test cut-off resulted in a notable loss of sensitivity, but gain in specificity. The detection of CIN3+ showed a higher sensitivity and a somewhat lower specificity compared to CIN2+.

**Table 2:** Pooled sensitivity and specificity for the detection of CIN2+ and CIN3+ in the triage HPV-positive women using colposcopy (at cut-off low-grade and high-grade colposcopic impression) and using cytology (at cut-off ASC-US, LSIL and HSIL).

	Test, cut-off	Outcome	N	P	Sensitivity		Specificity	
					Pooled estimate (95% CI)	I^2^	Pooled estimate (95% CI)	I^2^
Studies with complete follow-up	Colposcopy, low-grade impression	CIN2+	16	10,632	84.4 (79.0–88.6)	57.9	64.3 (57.4–70.7)	92.2
		CIN3+	15	10,550	86.9 (81.7–90.8)	43.5	62.2 (55.2–68.8)	93.7
	Colposcopy, high-grade impression	CIN2+	11	7195	35.5 (28.5–43.1)	54.5	95.3 (92.3–97.2)	80.3
		CIN3+	10	7113	44.5 (39.8–49.3)	15.7	95.0 (91.2–97.2)	87.1
	Cytology, ASC-US	CIN2+	14	6655	58.0 (44.7–70.2)	81.5	85.2 (79.8–89.3)	88.0
		CIN3+	13	6573	64.2 (48.5–77.4)	76.1	83.7 (77.7–88.4)	91.1
	Cytology, LSIL	CIN2+	11	6124	46.0 (33.0–59.6)	79.4	90.9 (87.1–93.6)	82.3
		CIN3+	11	6124	51.2 (37.2–65.0)	74.2	89.6 (85.3–92.8)	85.4
	Cytology, HSIL	CIN2+	12	6206	29.3 (19.5–41.4)	76.2	97.6 (96.1–98.5)	61.4
		CIN3+	11	6124	38.3 (24.7–54.0)	76.3	97.3 (95.3–98.5)	72.5
Studies with incomplete follow-up	Colposcopy, low-grade impression	CIN2+	6	2679	81.6 (75.5–86.4)	37.9	79.3 (71.2–85.6)	93.2
		CIN3+	4	1300	84.8 (75.0–91.2)	29.4	79.5 (68.9–87.2)	92.8
	Colposcopy, high-grade impression	CIN2+	1	462	58.3 (43.2–72.4)	–	96.1 (93.8–97.8)	–
		CIN3+	0	0	–	–	–	–
	Cytology, ASC-US	CIN2+	5	2200	76.0 (65.3–84.1)	65.2	79.6 (67.2–88.1)	95.5
		CIN3+	3	845	81.8 (69.1–90.0)	35.3	85.2 (82.2–87.8)	18.4
	Cytology, LSIL	CIN2+	5	2200	64.4 (49.4–77.0)	79.4	87.7 (81.6–92.0)	88.5
		CIN3+	3	845	72.6 (50.5–87.3)	70.1	89.9 (86.3–92.6)	50.8
	Cytology, HSIL	CIN2+	5	2200	46.6 (25.1–69.5)	90.5	96.0 (94.6–97.1)	37.6
		CIN3+	3	845	71.8 (49.4–86.9)	70.1	94.0 (91.8–95.6)	20.2

The meta-analysis is stratified by follow-up. Forest plots are available in the Supplemental results.

N = number of reports; P = number of participants; CI = confidence interval; I^2^ = heterogeneity index; ASC-US = atypical squamous cells of undetermined significance; LSIL = low-grade squamous intraepithelial lesion; HSIL = high-grade squamous intraepithelial lesion; CIN2+/CIN3+ = cervical intraepithelial neoplasia of grade 2 or 3 or worse.

**Fig. 3:**
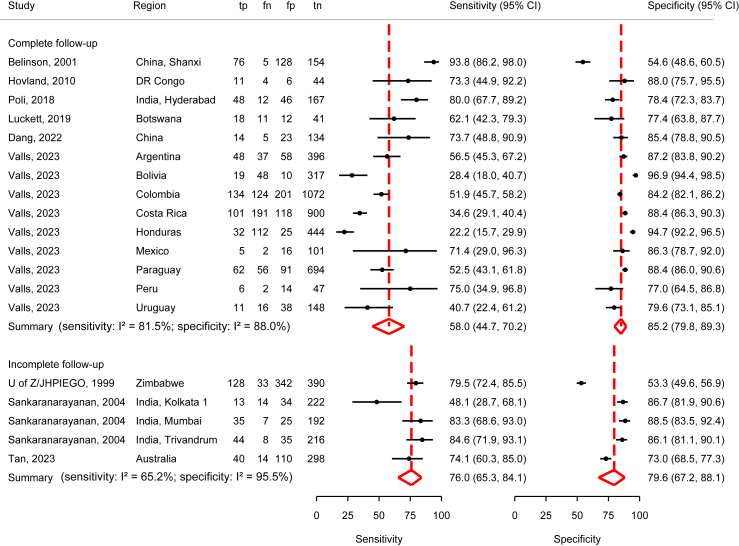
**Forest plot of the diagnostic accuracy of cytology triage.** Meta-analysis of the sensitivity (left) and specificity (right) for detection of CIN2+ in the triage of HPV-positive women using cytology with ASC-US as test cut-off. Top: studies with complete follow-up; Bottom: studies with incomplete follow-up. Abbreviations: tp = true positives; fn = false negatives; fp = false positives; tn = true negatives; CI = confidence interval; U of Z = University of Zimbabwe; ASC-US = atypical squamous cells of undetermined significance; CIN2+ = cervical intraepithelial neoplasia of grade 2 or worse.

The meta-analysis on the diagnostic accuracy for CIN2+ using cytology with ASC-US as test cut-off included 19 reports from 9 studies (Fig. 3). The sensitivity ranged from 22 to 94% and the specificity from 53 to 97%. Using only the studies with complete follow-up (14 reports from 6 studies), the pooled sensitivity and specificity were 58.0% (95% CI: 44.7–70.2%) and 85.2% (95% CI 79.8–89.3%). The observed heterogeneity was high: I^2^ was 81.5% for sensitivity and 88.0% for specificity. Studies with incomplete follow-up (6 reports from 3 studies) showed a higher pooled sensitivity (76.0%, 95% CI: 65.3–84.1%) and somewhat lower pooled specificity (79.6%, 95% CI: 67.2–88.1%) than studies with complete follow-up. More detailed results are available in Table 2 and the Supplementary Materials (page 16–18). In general, using a higher test cut-off resulted in a loss of sensitivity, but gain in specificity. The detection of CIN3+ showed a higher sensitivity and a lower specificity compared to CIN2+.

**Fig. 4:**
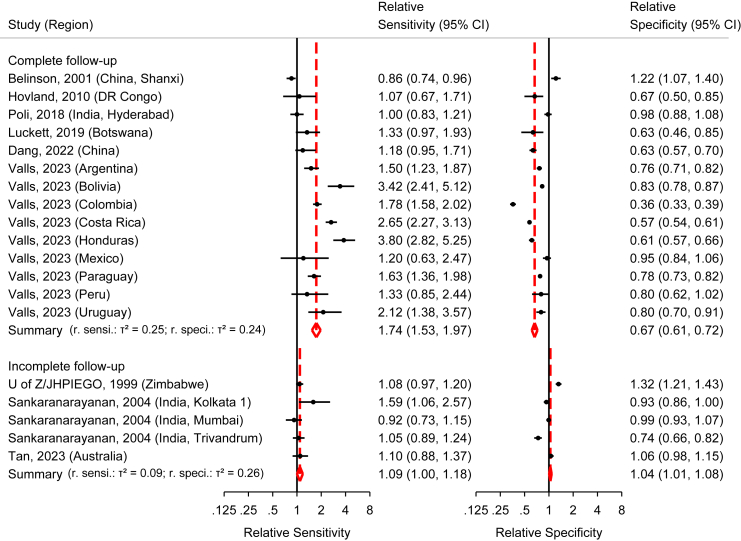
**Forest plot of the relative diagnostic accuracy of colposcopy triage compared to cytology triage.** Meta-analysis of the relative sensitivity (left) and specificity (right) for detection of CIN2+ in the triage of HPV-positive women using colposcopy with low-grade colposcopic impression as test cut-off compared to cytology with ASC-US as test cut-off. Top: studies with complete follow-up; bottom: studies with incomplete follow-up. Relative values > 1 indicate colposcopy had a higher value than cytology, while relative values < 1 indicate colposcopy had a lower value than cytology. Abbreviations: CI = confidence interval; r. sensi. = relative sensitivity; r. speci. = relative specificity; U of Z = University of Zimbabwe; ASC-US = atypical squamous cells of undetermined significance; CIN2+ = cervical intraepithelial neoplasia of grade 2 or worse.

The meta-analysis on the relative diagnostic accuracy of colposcopy (with cut-off low-grade colposcopic impression) compared to cytology (with cut-off ASC-US) for CIN2+ included 19 reports from 9 studies (Fig. 4). The relative sensitivity ranged from 0.86 to 3.80, with a pooled estimate based on studies with complete follow-up (14 reports from 6 studies) of 1.74 (95% CI: 1.53–1.97), indicating that on average colposcopy was more sensitive than cytology. In 17 out of 19 reports colposcopy was at least as sensitive as cytology. The relative sensitivity was significantly higher than one in 8 out of 19 reports, however 7 out of these 8 reports came from the ESTAMPA study.^23^ One report showed a relative sensitivity which was significantly lower than one, indicating that in this single report colposcopy was less sensitivity than cytology. The relative specificity ranged from 0.36 to 1.32, with a pooled estimate based on studies with complete follow-up of 0.67 (95% CI: 0.61–0.72), indicating that on average colposcopy is less specific than cytology. In 16 out of 19 records colposcopy was less specific than cytology. The relative specificity was significantly lower than one in 11 out of 19 reports, however 7 out of these came from the ESTAMPA study.^23^ Two reports showed a relative specificity which was significantly higher than one, indicating that in these two reports colposcopy was more specific than cytology. The pooled estimates from five reports with incomplete follow-up revealed slightly higher sensitivity and specificity of colposcopy compared to cytology. Relative diagnostic accuracy for CIN3+ and for different test cut-offs are available in Table 3 and corresponding forest plots are available in the Supplementary Materials (pages 19–24).

**Table 3:** Pooled relative sensitivity and relative specificity for the detection of CIN2+ and CIN3+ in the triage HPV-positive women using colposcopy (at cut-off low-grade and high-grade colposcopic impression) compared to using cytology (at cut-off ASC-US, LSIL and HSIL).

	Index test, cut-off	Comparator test, cut-off	Outcome	N	P	Pooled ratio (95% CI)	
						Sensitivity	Specificity
Studies with complete follow-up	Colposcopy, low-grade impression	Cytology, ASC-US	CIN2+	14	7470	1.74 (1.53–1.97)	0.67 (0.61–0.72)
			CIN3+	13	7388	1.58 (1.35–1.85)	0.65 (0.59–0.70)
		Cytology, LSIL	CIN2+	11	6272	2.25 (1.87–2.72)	0.63 (0.57–0.70)
			CIN3+	11	6272	2.06 (1.68–2.54)	0.60 (0.54–0.67)
		Cytology, HSIL	CIN2+	12	6354	3.42 (2.67–4.38)	0.61 (0.54–0.69)
			CIN3+	11	6272	2.70 (1.99–3.65)	0.59 (0.52–0.67)
	Colposcopy, high-grade impression	Cytology, ASC-US	CIN2+	11	5931	0.95 (0.86–1.06)	1.08 (1.05–1.11)
			CIN3+	9	5849	0.99 (0.89–1.11)	1.07 (1.05–1.09)
		Cytology, LSIL	CIN2+	9	5849	1.26 (1.12–1.42)	1.02 (1.01–1.03)
			CIN3+	9	5849	1.24 (1.10–1.40)	1.01 (1.00–1.02)
		Cytology, HSIL	CIN2+	10	5931	2.08 (1.78–2.43)	0.96 (0.95–0.98)
			CIN3+	9	5849	1.85 (1.58–2.17)	0.96 (0.94–0.97)

The meta-analysis only includes studies with complete follow-up. Forest plots are available in the Supplemental results.

N = number of reports; P = number of participants; CI = confidence interval; ASC-US = atypical squamous cells of undetermined significance; LSIL = low-grade squamous intraepithelial lesion; HSIL = high-grade squamous intraepithelial lesion; CIN2+/CIN3+ = cervical intraepithelial neoplasia of grade 2 or 3 or worse.

Leave-one-out meta-analysis of the diagnostic accuracy for detection of CIN2+ in the triage of HPV-positive women using colposcopy (low-grade colposcopic impression) resulted in similar sensitivity (range: 84.3% to 85.7%) and specificity (range: 63.1% to 65.1%) pooled estimates as the main analysis, except when the ESTAMPA study^23^ was left out (sensitivity: 76.7% (95% CI: 67.9–83.7%), specificity: 66.2% (95% CI: 58.3–73.2%)). Leave-one-out meta-analysis of the relative diagnostic accuracy of colposcopy (low-grade colposcopic impression) compared to cytology (ASC-US) showed a similar trend. Pooled relative sensitivity ranged from 1.75 to 1.88 and pooled relative specificity ranged from 0.65 to 0.67, except when the ESTAMPA study was left out (relative sensitivity: 1.00 (95% CI: 0.92; 1.09), relative specificity: 0.87 (95% CI: 0.82; 0.94)). The complete results of the leave-one-out meta-analysis are available in the Supplementary Materials (Page 27).

The positive predictive value (PPV) and the complement of the negative predictive value (cNPV) for CIN2+ and CIN3+ were calculated using the pooled sensitivity and specificity estimates from studies with complete follow-up and assuming a moderate disease prevalence (14.0% for CIN2+, 9.0% for CIN3+). Triage using colposcopy (low-grade colposcopic impression) had a CIN2+ PPV of 27.8% and cNPV of 3.8%, and a CIN3+ PPV of 18.5% and cNPV of 2.1%. Triage using cytology (ASC-US) had a CIN2+ PPV of 38.9% and cNPV of 7.4%, and a CIN3+ PPV of 28.2% and cNPV of 4.0%. If these estimates are applied to a population of 1000 HPV-positive women, triage using colposcopy would find 17 additional cases of CIN2 and 20 additional cases of CIN3+ compared to triage using cytology (Supplementary Materials, page 32–34).

All 1000 HPV-positive women would have colposcopy and 425 would receive treatment after a positive colposcopy result. Among the 425 women receiving treatment, there would be 307 with CIN1 or less severe disease (over-treatment), and 40 cases with CIN2 and 78 CIN3+ cases. This corresponds to 8.5 colposcopies and 3.6 treatments (of which 2.6 over-treatments) per CIN2+ detected. Finally, ten CIN2 and 12 CIN3+ cases would be missed by colposcopy triage.

Calculating the same numbers for triage using cytology (ASC-US), we find that 208 HPV-positive women would receive colposcopy and treatment after a positive cytology result. Among the 208 women receiving colposcopy and treatment, there would be 127 with only ≤ CIN1 (over-treatment), 58 cases with CIN2 and 23 with CIN3+. This corresponds to 2.6 colposcopies and treatments (of which 1.6 over-treatments) per CIN2+ found. Finally, 27 CIN2 and 32 CIN3+ cases would be missed by cytology triage.

There was some indication that sample size effects were present, in particular when evaluating colposcopy at cut-off low-grade colposcopic impression (CIN2+: p = 0.049; CIN3+: p = 0.019) and cytology at cut-off ASC-US (CIN2+: p = 0.024; CIN3+: p = 0.042). The results of Deeks’ regression test and the Deeks’ funnel plots are available in the Supplementary Materials (page 28–31).

Certainty of evidence regarding triage of HPV-positive women was judged as low for the eight studies with complete follow-up and very low considering all the eleven studies (Supplementary Materials, page 35–38). The most important factors that decreased certainty in evidence were subjectivity in the assessment of colposcopy, cytology and histopathology, the high heterogeneity in absolute and relative diagnostic accuracy between studies and the risk of reference standard misclassification because of lack of blinding with the evaluated tests.

## Discussion

We conducted the first systematic review and meta-analysis on the diagnostic accuracy of colposcopy to triage women with an HPV-positive result at cervical cancer screening. We reported both absolute diagnostic accuracy and relative diagnostic accuracy compared to cytology triage at multiple cut-offs.

The included studies were conducted in different geographical regions and enrolled target populations who differed in age, pretest risk and HIV prevalence. The diversity in study characteristics reflects the wide variety of settings in which cervical cancer screening occurs. As a consequence the diagnostic accuracy reported by the individual studies was heterogenous which resulted in a low certainty of evidence. Therefore the pooled estimates should be interpreted with caution. Nevertheless a clear trend was observed wherein the majority of studies reported similar or higher sensitivity for triage with colposcopy compared to triage with cytology, including three studies that reported high HIV prevalence or specifically targeted women living with HIV. A leave-one-out meta-analysis that excluded a large study with high sensitivity for triage using colposcopy (the ESTAMPA study)^23^ still resulted in a lower pooled sensitivity estimate, yet one that remained comparable to that of cytology. Thus, despite substantial variability in absolute sensitivity across studies, evidence consistently indicates that colposcopy triage demonstrates at least similar sensitivity to cytology triage despite the difference in background settings. This indicates a high level of consistency despite the variety in background settings. As a trade-off, most studies reported equal or lower specificity of triage with colposcopy compared to triage with cytology. In other words, triage with colposcopy is likely to detect at least as many cases of cervical (pre-)cancer than triage with cytology, but might also lead to over-diagnosis. This finding is consistent with the performance of colposcopy in other contexts and also aligns with previous findings on cytology as a triage test, which have shown limited and highly variable sensitivity across study centres.^25^ A previous systematic review evaluating colposcopy as a primary screening strategy also reported heterogeneous results, with high sensitivity but low specificity for colposcopy screening.^26^

A limitation of this systematic review is the lack of available data. While many studies use colposcopy followed by histological assessment to diagnose cervical (pre-)cancer, the accuracy of colposcopy is often not reported as it is generally not the primary focus of the study. Certain authors who were contacted to share their results did not respond, leaving a substantial amount of data unavailable. Moreover, colposcopy and colposcopy-target biopsies often are part of the disease verification, complicating the accuracy assessment of colposcopy as a test. Funnel plots indicated a small sample size effect which could be caused by publication bias.^23^ Given that colposcopy is one of the recommended triage strategies in the WHO guidelines for cervical cancer screening, it is critical for authors to report or share their findings. The influence of colposcopy on disease verification could be mitigated by taking one or more blind biopsies.^18^ In the ESTAMPA study, the risk of missing cervical disease was mitigated by adding a disease verification step, 18 months after primary screening, for women with persisting HPV-positivity and initially negative colposcopy.^27^

As part of the systematic review we assessed the study quality using the QUADAS-2 criteria. A potential source of bias in all studies is the inherent link between colposcopy and the reference standard. The decision on which reference standard to use will depend in some part on the colposcopist’s ability to correctly identify lesions, the number of biopsies that can be collected, etc. Most notably, in three out of eleven studies.^16^^,^^17^^,^^20^ HPV-positive women with negative colposcopy were assumed to be disease-free and no biopsy was taken. However, the remaining eight studies^13^, ^14^, ^15^^,^^18^^,^^19^^,^^21^, ^22^, ^23^ demonstrated that cervical (pre-)cancer was occasionally still found among women with negative colposcopy, demonstrating that an imperfect reference standard might introduce verification bias and lead to inflated sensitivity and specificity estimates. The eight studies with complete follow-up reduced the risk of verification bias by either collecting one or more random biopsies irrespective of the colposcopy findings,^13^, ^14^, ^15^^,^^18^^,^^19^^,^^21^ which may make histology less dependent on colposcopy and consequently result in reduced gold-standard misclassification associated with colposcopic findings, or by recalling all HPV-positive women with negative colposcopy, negative histology or no disease definition for any other reason to a follow-up HPV test and referring all HPV-positive women at follow-up to colposcopy.^22^^,^^23^ In case of the latter, the initial colposcopy is used as the triage result, while the second follow-up colposcopy contributes to the diagnosis of the outcome. We addressed this issue by stratifying the meta-analysis by completeness of follow-up and found that studies with complete follow-up consistently reported similar or better sensitivity for triage with colposcopy compared to triage with cytology. While this finding strengthens our conclusions, the risk of verification bias in colposcopy studies can never be fully mitigated, ultimately contributing to a low certainty of evidence.

We provided an overview of study characteristics, which identified several factors that could contribute to the observed heterogeneity in diagnostic test accuracy. However, as is common in meta-analyses using aggregated data, inconsistent reporting prevents the quantitative assessment of influence of possible sources of heterogeneity in subgroup analysis or meta-regression. The information provided by studies depends on the type and focus of the study. For example, a retrospective study using hospital and registry data might provide only minimal details on the conduct of colposcopy, while a prospective cohort study focusing on colposcopy as a triage strategy might report on many aspects of colposcopy.

An additional complication was the large influence of the ESTAMPA study,^23^ which was reflected in the sensitivity analysis. Among studies with complete follow-up, the ESTAMPA study accounted for 5849/10,632 participants (>50%) in the meta-analysis of diagnostic test accuracy of colposcopy to detect CIN2+ and for 5849/7470 participants (>70%) in the meta-analysis of the relative accuracy of colposcopy compared to cytology to detect CIN2+. The ESTAMPA study reported good diagnostic test accuracy for colposcopy, which the authors attributed to their high standard quality-assured protocol leading to high participant compliance and ensuring colposcopies were done by sufficiently equipped, trained, and supervised colposcopists. In a simple subgroup analysis any factor associated with the ESTAMPA study would seem to have a positive effect on the accuracy of colposcopy due to the study’s large influence, regardless of its true underlying effect (i.e., confounding).

While we were unable to perform meta-regression and subgroup analysis, we still provide a discussion of several factors that could contribute to heterogeneity.

Few studies reported on the competency or training of cytologists, colposcopists, or histopathologists. While competency and training are generally considered to have an impact on the diagnostic test accuracy of subjective assessments, previous research evaluating this topic have yielded inconclusive results.^28^, ^29^, ^30^ Another interesting factor is the variation between studies in the number and type of biopsies taken, ranging from studies where a single colposcopy-guided biopsy was taken to studies where multiple lesions were biopsied and random biopsies were taken in case no lesions were observed. Additionally, some studies included or required endocervical curettage. Previous research has demonstrated that colposcopy-guided biopsy alone is not a perfect reference standard, and that increasing the number of biopsies improves the detection rate of cervical (pre-)cancer.^28^^,^^29^ Improved reporting would allow future systematic reviews to address the impact of these factors.

The inconsistent reporting on test blinding complicated the systematic review and lowered the certainty of evidence. When studies evaluate multiple tests, blinding is often not fully reported for all tests and only a few studies reported on blinding of the reference test. Lack of blinding could lead to outcome misclassification. A previous study showed reduced sensitivity after adjusting for misclassification bias,^31^ while other studies showed that the performance of colposcopy improved with cytological grade when the colposcopic assessment was unblinded.^23^ If colposcopy, cytology and histological assessment are not performed independently of each other, the test assessor might be more likely to interpret a test as positive when other test results were also positive. In addition, for cytology, it has been reported that unblinding cytologists to HPV positivity increased cytology sensitivity and decreased the specificity, but the results remained highly variable.^25^ The potential impact of outcome misclassification depends on which tests were blinded to each other, emphasizing the importance of reporting on blinding for all tests including comparator and reference tests.

Other factors that could explain heterogeneity which were reported inconsistently and therefore were not addressed by this systematic review are the type of transformation zone, colposcopy scoring (e.g., Swede Score), HPV vaccination status, HPV type and persistence of HPV infection. Additionally, age may play a role in heterogeneity; the ESTAMPA study^23^ found higher sensitivity in women aged 30–49 compared to those aged 50–65, highlighting the difficulties of visualizing the squamocolumnar junction in older women due to biological factors such as menopause and hormone-related changes and emphasizing the need of sampling the endocervical canal.

Both colposcopy and cytology are inherently subjective tests and prone to interobserver variability. In this review we found that the sensitivity of colposcopy triage was less heterogeneous across studies than the sensitivity of cytology triage, with smaller I^2^ values and narrower confidence intervals around the pooled estimate. While the lowest reported sensitivity for colposcopy (low-grade colposcopic impression) to detect CIN2+ was 62%, the lowest reported sensitivity for cytology (ASC-US) was only 22%. This suggests that colposcopy triage can detect CIN2+ and CIN3+ cases more reliably than cytology triage. While this systematic review did not compare triage using colposcopy to triage using VIA, it is accepted that the performance of triage using VIA is highly variable and prone to challenges with quality assurance.^1^^,^^30^ While VIA with the naked eye can identify obvious lesions, a well-trained colposcopist is able to identify less obvious lesions resulting in a more robust assessment.

Access to colposcopy and interpretation of colposcopy results will be facilitated by further research on the use of portable colposcopy devices supported by artificial intelligence, spectroscopy and other adjunctive tools. The overall screening process can be further optimised by using point-of-care HPV test and HPV partial genotyping. The information on HPV types and the colposcopic impression could be used to make swift informed clinical decisions, e.g., providing immediate treatment to all women who tested HPV16 or HPV16/18 positive and had an abnormal colposcopy without waiting for a histology result. Future systematic reviews should evaluate the innovative colposcopy devices as an alternative point-of-care triage method to manage HPV positive women in low-resource settings.

Our meta-analysis was restricted to women attending screening with an HPV DNA assay. However, screening with a validated HPV mRNA assay may also be used according to the WHO guidelines.^32^ Referring HPV mRNA positive women to colposcopy may yield a slightly higher CIN3+ risk among women with suspicious colposcopy findings compared to HPV DNA positive women and a similarly low CIN3+ risk if colposcopy does not reveal abnormalities.^33^ We acknowledge that the HPV DNA assays used as screening tests in the included studies belong to the first generation comparator tests (HC2 and GP5+/6+ PCR). However, newer *second generation* HPV assays show similar accuracy compared to these older assays.^34^

In conclusion, despite heterogeneous results, studies consistently reported similar or higher sensitivity but lower specificity for CIN2+/CIN3+ when using colposcopy at low-grade colposcopic impression compared to cytology at ASC-US to triage HPV-positive women. While the choice of triage tests will ultimately depend on multiple factors, these results indicate that colposcopy may be one of several acceptable triage tests, particularly in settings where recall of women after a positive screening test is difficult and colposcopy is available. While these findings are consistent with WHO guidelines on management of screen-positive women, only low certainty of evidence can be attributed to recommend colposcopy to triage women with HPV because of the variable diagnostic accuracy results.

Since conventional colposcopy often not is available in resource-poor settings, more research is necessary to evaluate the performance of portable colposcopy devices and of artificial intelligence-assisted interpretation of colposcopic images. These novel techniques could lead to new screen, triage and treat approaches, especially when combined with point-of-care HPV tests.

## Contributors

MAr and MAl conceived the scope of the review. MAr and IJ formulated the clinical question, identified PICOS components and developed the protocol. ND, RR, IJ, LD and ATR identified studies and extracted the data. ND did the statistical analyses IJ wrote the protocol and ND wrote the first draft of the manuscript. All authors critically reviewed the manuscript, contributed to the interpretation of the results and manuscript writing, and approved the final version. ND, MAl and MAr had final responsibility for the decision to submit for publication. All authors had full access and ND, IJ, RR and MAr verified the underlying study data.

## Data sharing statement

Researchers wishing to undertake additional analyses of the data are invited to contact the corresponding author. The study protocol and analysis plan are available on PROSPERO at https://www.crd.york.ac.uk/PROSPERO/view/CRD42023389772.

## Declaration of interests

The authors declare no competing interests.

## Appendix A Supplementary data

Supplementary materials

Translated Abstracts
