# Supplementary material for: Accuracy of colposcopy to triage HPV-positive women in cervical cancer screening: a systematic review and meta-analysis
Source: eClinicalMedicine. 2026 Jun 17;96:104009. doi: 10.1016/j.eclinm.2026.104009 (PMC13311182; doi:10.1016/j.eclinm.2026.104009)
Supplement: Supplementary materials [file mmc1.docx]

Supplementary materials

1. Study protocol 2

1.1. Objectives 2

1.2. Clinical questions 2

1.3. PICOS 2

1.4. Search strings 2

2. Study characteristics of the included studies 4

2.1. Study design, population and screening algorithm of the included studies 4

2.2. Index, comparator and reference test characteristics of the included studies 8

3. Risk of bias of the included studies 12

4. Diagnostic accuracy of colposcopy 14

5. Diagnostic accuracy of cytology 16

6. Relative diagnostic accuracy of colposcopy compared to cytology 19

7. SROC curves 25

8. Sensitivity analysis 27

9. Sample size effects 28

10. Positive and negative predictive values 32

11. Certainty of evidence 35

12. References 39

2. Study protocol

### Objectives

Our primary objectives include evaluating the absolute accuracy to detect cervical pre-cancer or cancer of colposcopy as triage of hrHPV-positive women as well as the relative accuracy to detect cervical pre-cancer or cancer of colposcopy in comparison to cytology as a triage test of hrHPV-positive women. Secondary objectives include assessing the variation in the accuracy of colposcope by patients’ characteristics, and technical characteristics of the colposcopy. Other secondary objectives include assessment of unsatisfactory colposcopy results, test-positivity rate, positive predictive value, and detection rate of cervical pre-cancer and cancer. These findings will help optimise the application of colposcopy in the triage of hrHPV+ women which comes in line with the 2020 WHO cervical cancer global elimination strategy.

### Clinical questions

The study questions were: 1) What is the diagnostic performance of colposcopy to detect cervical precancerous lesions or cancer among women with a positive hrHPV test result at cervical screening? and 2) Is colposcopy more accurate than cervical cytology (comparator test) in triage of HPV positive women?

### PICOS

Population: women participating in cervical cancer screening who had a positive hrHPV-DNA test result

Index test: colposcopic impression (cut-off is low-grade or high-grade lesions)

Comparator test: cytology (cut-off is ASC-US, LSIL or HSIL)

Outcome: sensitivity and specificity to detect CIN2+ and CIN3+

Study: randomised trials

complete diagnostic studies (all women were tested with the index test, comparator test and were verified with reference standard)

incomplete diagnostic studies (all women were tested with index test or comparator test and were verified with reference standard of by comprehensive follow-up or through cancer registries)

### Search strings

**supplemental Table 1.** Literature retrieval strings in PubMed-Medline

| #1: Cervix OR cervico* OR cervica* |
| --- |
| #2: Cancer OR carcinoma OR neoplas* OR dysplas* OR CIN[tw] OR CINII*[tw] OR CIN2*[tw] OR CINIII*[tw] OR CIN3[tw] OR SIL[tw] OR SIL OR HSIL[tw] OR H-SIL OR LSIL[tw] OR L-SIL OR ‘‘low grade’’ OR low-grade OR mild OR equivocal OR borderline. |
| #3: #1 AND #2. |
| #4: HPV OR "Human Papillomavirus DNA Tests"[Mesh] OR ‘‘human papillomavirus’’ OR papillomavir* OR viral OR virus |
| #5: colposcopy OR “colposcopes” OR " colposcopy "[Mesh] |
| #6: #4 AND #5 |
| #7: #3 AND #6 |
| #8: Publication Date to 20 October, 2025 |
| #9: #7 AND #8 |

**supplemental Table 2.** Literature retrieval strings in Embase.

| #1: 'cervix'/exp OR cervix OR cervico* OR cervica* |
| --- |
| #2: 'cancer'/exp OR cancer OR 'carcinoma'/exp OR carcinoma OR neoplas* OR dysplas* OR cin OR 'cin2' OR 'cin3' OR sil OR hsil OR h+sil OR lsil OR l+sil OR 'low grade' OR low+grade OR mild OR equivocal OR 'borderline'/exp OR borderline |
| #3: 'hpv'/exp OR hpv OR 'human papillomavirus'/exp OR 'human papillomavirus' OR papillomavir* OR viral OR 'virus'/exp OR virus |
| #4 'colposcopy'/exp OR 'colposcopes* |
| #5: #1 AND #2 AND #3 AND #4 |
| With the following limits:  - Map to preferred terminology (with spell check)  - Also search as free text  - Include sub-terms/derivatives (explosion search)  - Search publications: all years |

**SUPPLEMENTAL TABLE 3.** Literature retrieval strings in the Cochrane Library.

| #1: Cervix or cervico* or cervica* |
| --- |
| #2: Cancer or carcinoma or neoplas* or dysplas* or CIN or CIN2 or CIN3 or SIL or SIL or HSIL or H-SIL or LSIL or L-SIL or "low grade" or low-grade or mild or equivocal or borderline. |
| #3: HPV or ‘‘human papillomavirus’’ or papillomavir* or viral or virus |
| #4: ’colposcop* |
| With the following limits:   - Cochrane reviews (reviews + protocols) - Other reviews |

Note:

- PubMed was searched until 20^th^ October 2025.
- Embase and the Cochrane library were searched until 28^th^ February, 2024. This search did not find any additional eligible studies beyond those found in PubMed. Therefore it was decided to search only PubMed from this point onward.

1. Study characteristics of the included studies

### Study design, population and screening algorithm of the included studies

**supplemental Table 4.** Study design, population and screening algorithm of the included studies.

| **Author, year**  *Study*  Region, study period | **N** | **Study design and population** | **Screening tests** | **Follow-up algorithm & reference standard** |
| --- | --- | --- | --- | --- |
| **University of Zimbabwe /JHPIEGO, 1999^1-3^**  Zimbabwe, 1995-1997 | 917 | Cross-sectional.  Population: women attending primary care clinics. High prevalence of HIV.  Age: 25-55.  Exclusion: currently pregnant, hysterectomy, history of cervical cancer. | All women were screened with HPV, cyto­logy, VIA and colpo­scopy.  HPV test: HC2 on a cervical sample. | Biopsies were taken if colposcopy was abnormal.  Reference standard: negative colposcopy or histological assessment. |
| **Belinson, 2001^4^**  *SPOCCS I*  China, 1999 | 363 | Cross-sectional.  Population: women invited to attend screening.  Age: 35-45. *  Exclusion: currently pregnant, hysterectomy, history of pelvic radiation, history of cervical cancer screening. | All women were screened with HPV, cyto­logy, VIA and colpo­scopy and biopsy.  HPV test: HC2 on a cervical sample taken by a gynaecologic oncologist or their fellow. | All women had biopsy and ECC. No follow-up.  Reference standard: histological assessment. |
| **Belinson, 2003^5^**  *SPOCCS II*  China, 2001 | 2074 | Mixture of cross-sectional and prospective.  Population: women invited to attend screening.  Age: 27-56. *  Exclusion: currently pregnant, hysterectomy, history of pelvic radiation. | All women were screened (1) at home with HPV (self-sample) and (2) 3-18 months later with HPV (physician-sample) and cytology.  HPV test: HC2 on a physician obtained sample. | Women who were HPV+ on self-test received colposcopy together with HPV (physician-sample) and cytology. Women who were HPV+ on physician-sample or cytology+ were recalled for colposcopy. Biopsies and ECC were taken at every colposcopy.  Reference standard: histological assessment. |
| **Sankaranarayanan, 2004^6^**  India, 1999-2003 | 1300 | Cross-sectional.  Population: women invited to attend screening. None of the participating women had previously been screened for cervical neoplasia.  Age: 25-65. *  Exclusion: non-intact uterus, history of cervical neoplasia, appearing unhealthy or symptomatic. | All women were screened with HPV, VIA, VILI and colposcopy. In the Kolkata-1, Mumbai and Trivandrum study all women were also screened with cytology.  HPV test: HC2 on a sample taken by a trained health worker. | Biopsies were taken if colposcopy was abnormal.  Reference standard: negative colposcopy or histological assessment. |
| **Hovland, 2010^7^**  Democratic Republic of Congo, 2003 | 65 | Cross-sectional.  Population: women attending gynaecological clinics.  Age: 25-60. *  Exclusion: currently pregnant, hysterectomy, severe gynaecological bleeding. | All women were screened with HPV, cytology, colposcopy and biopsy.  HPV test: HPV DNA GP5+/6+ PCR on a clinician sample. | All women had biopsy. No follow-up.  Reference standard: histological assessment. |
| **Poli, 2018^8^**  *START-UP*  India, 2010-2013 | 273 | Mixture of cross-sectional and prospective.  Population: women residing in urban slums were invited to attend screening.  Age: 30-49.  Exclusion: currently pregnant, non-intact uterus, history of cervical (pre-)cancer, not married. | All women were screened with HPV, cytology and VIA.  HPV test: *care*HPV on a self-collected vaginal sample. | VIA+ women received colposcopy immediately. VIA- but HPV+ or cytology+ women were recalled within 1-4 weeks for colposcopy. Biopsies were taken at every colposcopy.  Reference standard: histological assessment. |
| **Luckett, 2019^9^**  Botswana, 2018 | 82 | Prospective cohort.  Population: WLHIV attending an infectious disease care clinic.  Age: ≥ 25.  Exclusion: currently pregnant, hysterectomy, history of cervical cancer, currently menstruating heavily or with persistent vaginal discharge. | All women were screened with HPV test and cytology.  HPV test: Xpert® HPV assay on clinician taken sample. | All HPV+ women, HSIL+ women and ASC-US+ women who had a prior abnormal cytology result were recalled for VIA and colposcopy. Biopsies were taken at every colposcopy.  Reference standard: histological assessment. |
| **Dang, 2022^12,13^**  China, 2015-2018 | 843 | Prospective cohort. Only including the study arm of rural women assigned to HPV screening. 24 month follow-up visit.  Population: women attending screening.  Age: 35-64.  Exclusion: currently pregnant, hysterectomy, history of cervical cancer, not sexually active. | All women were screened with HPV.  HPV test: *care*HPV on a cervical sample. | HPV+ women were randomised VIA/VILI triage or cytology triage or direct colposcopy. A biopsy was taken in case of high-grade cytology abnormalities or abnormal colposcopy. All women except those detected with CIN2+ were called back at 24 months for follow-up screening. At follow-up screening, women were simultaneously screened with HPV, cytology and VIA/VILI and those with any positive results were referred to colposcopy directly.  Reference standard: negative screening at baseline and 24 month follow-up, histological assessment. |
| **Luckett, 2023^14^**  Botswana, 2021-2022 | 1264 | Prospective cohort.  Population: women seeking care at a health facility, accompanying someone seeking care at a health facility or working at or near the health facility. Cohort enriched to include 50% WLHIV.  Age: 25-77. *  Exclusion: currently pregnant, hysterectomy, history of cervical cancer. | All women were screened with HPV.  HPV test: AmpFire® HPV Assay on vaginal self-sample. | HPV+ women were recalled to VIA, colposcopy and biopsy.  Reference standard: histological assessment. |
| **Valls, 2023^10,11^**  *ESTAMPA*  Latin America, 2013-2022 | 5849 | Mixture of cross-sectional and prospective. 18 month follow-up visit.  Population: women were recruited from primary and secondary care centres, hospitals, laboratories and universities.  Age: 30-64.  Exclusion: hysterectomy, history of cervical (pre-)cancer, not sexually active, planning to move outside of the study area. | All women were screened with HPV and cytology.  HPV test: HC2 or cobas® 4800 on a cervical sample. | Screen+ women were referred to colposcopy or to VIA followed by colposcopy. 2-3 biopsies were taken in case of abnormal colposcopy (or endocervical cytobrush or LLETZ). Women with normal colposcopy or with negative histology (< CIN2) were recalled at 18 months.  At the 18 month visit, women were screened with HPV and those with a positive result were referred to colposcopy.  Reference standard: negative colposcopy at baseline and negative screening at the 18 month follow-up visit, histological assessment. |
| **Tan, 2023^15^**  Australia, 2018-2020 | 462 | Retrospective observational study. 24 month follow-up.  Population: women seen at a colposcopy clinic after a positive HPV screening test.  Age: 50-74.  Exclusion: hysterectomy, history of vaginal intraepithelial neoplasia. | All women had a positive HPV screening test.  HPV test: clinically validated HPV assays. | All women had colposcopy at the initial visit. Most women had (1) biopsy at the initial visit or (2) a follow-up HPV, cytology or colposcopy within 24 months.  Reference standard: negative colposcopy at the initial visit and no follow-up results, negative colposcopy at initial visit and negative screening and/or colposcopy during 24 month follow-up, histological assessment. |
| Abbreviations: N = number of HPV+ participants included in the meta-analysis; WLHIV = women living with HIV; * = age range reported for entire study population, i.e. for both HPV+ and HPV- participants; LLETZ = large loop excision of the transformation zone. | | | | |

### Index, comparator and reference test characteristics of the included studies

**supplemental Table 5.** Index, comparator and reference test characteristics of the included studies

| **Author, year**  *Study*  Region, study period | **N** | **Colposcopy (index test)** | **Cytology (comparator test)** | **Histological assessment (reference standard)** |
| --- | --- | --- | --- | --- |
| **University of Zimbabwe /JHPIEGO, 1999^1-3^**  Zimbabwe, 1995-1997 | 917 | Colposcopist: investigators at central clinic.  Blinding: blinded to cytology and VIA; not reported if blinded to HPV.  Threshold: low threshold (low-grade squamous intraepithelial lesion or worse) | Conventional cytology.  Cytologist: cytotechnicians who followed additional training in preparation of the study.  Second assessment: a cytopathologist reviewed all positive and 10% of negative smears.  Blinding: blinded to VIA; not reported if blinded to HPV.  Threshold: negative, ASC-US, LSIL, HSIL. | Biopsies & ECC: targeted biopsies.  Histopathologist: not reported.  Blinding: blinded to cytology, VIA; unblinded to colposcopy; not reported if blinded to HPV.  Outcome: <CIN2, CIN2+. |
| **Belinson, 2001^4^**  *SPOCCS I*  China, 1999 | 363 | Colposcopist: gynaecologic oncologists.  Blinding: not reported.  Threshold: negative, any abnormal. | Liquid-based cytology (ThinPrep).  Cytologist: cyto­patho­logist.  Second assessment: a cytopathologist reviewed all positive and 5% of negative smears.  Blinding: not reported.  Threshold: negative, ASC-US, LSIL, HSIL. | Biopsies & ECC: biopsy per quadrant: targeted biopsy in case of visible lesions, 4-quadrant random biopsy in case of no visible lesions. ECC was also taken.  Histopathologist: pathologist.  Blinding: blinded to screening test results.  Outcome: <CIN2, CIN2+, CIN3+. |
| **Belinson, 2003^5^**  *SPOCCS II, 2001*  China | 2074 | Colposcopist: gynaecologic oncologists.  Blinding: not reported.  Threshold: negative, any abnormal. | / | Biopsies & ECC: biopsy per quadrant: targeted biopsy in case of visible lesions, 4-quadrant random biopsy in case of no visible lesions. ECC was also taken.  Histopathologist: pathologist.  Blinding: blinded to screening test results.  Outcome: <CIN2, CIN2+, CIN3+. |
| **Sankaranarayanan, 2004^6^**  India, 1999-2003 | 1300 | Colposcopist: doctors (12 gynaecologists and 2 non-gynaecologists) who followed additional training in preparation of the study.  Blinding: blinded to other screening test results.  Threshold: negative, any abnormal. | Conventional cytology.  Cytologist: not reported.  Second assessment: not reported.  Blinding: not reported.  Threshold: negative, ASC-US, LSIL, HSIL | Biopsies & ECC: targeted punch biopsies.  Histopathologist: pathologist. refresher course.  Blinding: unblinded to colposcopy; blinded to HPV, cytology, VIA, VILI.  Outcome: <CIN2, CIN2+ CIN3+. |
| **Hovland, 2010^7^**  Democratic Republic of Congo, 2003 | 65 | Colposcopist: joint examination by two physicians (gynaecologists or GPs).  Blinding: blinded to other screening test results.  Threshold: negative, any abnormal, high-grade. | Conventional cytology and liquid-based cytology (ThinPrep).  Cytologist: conventional pap smears were evaluated by a well-experienced cytotechnician.  Second assessment: a cytopathologist reviewed all positive smears.  Blinding: blinded to other screening tests result.  Threshold: negative, ASC-US, LSIL, HSIL | Biopsies & ECC: targeted biopsies in case of visible lesions, biopsy at 12 o’clock if no visible lesions. ECC was possible.  Histopathologist: pathologist.  Blinding: blinded to screening test results.  Outcome: <CIN2, CIN2+, CIN3+. |
| **Poli, 2018^8^**  *START-UP*  India, 2010-2013 | 273 | Colposcopist: trained medical officer.  Blinding: not reported.  Threshold: negative, minor-grade. | Conventional cytology.  Cytologist: pathologist.  Second assessment: not reported.  Blinding: not reported.  Threshold: negative, ASC-US. | Biopsies & ECC: targeted biopsies in case of visible lesions, 12 o’clock in case of no visible lesions.  Histopathologist: not reported.  Blinding: not reported.  Outcome: <CIN2, CIN2+, CIN3+. |
| **Luckett, 2019^9^**  Botswana, 2018 | 82 | Colposcopist: gynaecologist.  Blinding: blinded to HPV, cytology and VIA test results.  Threshold: negative, low-grade impression, high-grade impression. | Conventional cytology  Cytologist: pathologist.  Second assessment: not reported.  Blinding: blinded to VIA and colposcopy result, not reported if blinded to HPV.  Threshold: negative, ASC-US, HSIL | Biopsies & ECC: targeted punch biopsy or LEEP in case of visible lesions, small endocervical excision or ECC in case of no visible lesions.  Histopathologist: not reported.  Blinding: not reported.  Outcome: <CIN2, CIN2+. |
| **Dang, 2022^12,13^**  China, 2015-2018 | 843 | Colposcopist: physician.  Blinding: not reported.  Threshold: negative, positive | Liquid-based cytology.  Cytologist: pathologist.  Second assessment: a pathologist all positive and 10% of negative smears.  Blinding: not reported.  Threshold: negative, ASC-US | Biopsies & ECC: targeted biopsy in case of visible lesions, 4-quadrant random biopsy and ECC in case of no visible lesions.  Histopathologist: pathologist.  Blinding: not reported.  Outcome: <CIN2, CIN2+, CIN3+. |
| **Luckett, 2023^14^**  Botswana, 2021-2022 | 1264 | Colposcopist: gynaecologist.  Blinding: unblinded to HPV, blinded to VIA.  Threshold: normal, low-grade impression, high grade impression. | / | Biopsies & ECC: targeted punch biopsy or LEEP in case of visible lesions, small endocervical excision or ECC in case of no visible lesions.  Histopathologist: not reported.  Blinding: not reported.  Outcome: <CIN2, CIN2+, CIN3+. |
| **Valls, 2023^10,11^**  *ESTAMPA*  Latin America, 2013-2022 | 5849 | Colposcopist: experienced colposcopists who followed additional training in preparation of the study.  Blinding: not reported if blinded to HPV; unblinded to cytology results; in some centres VIA was performed and in case of suspected cancer the colposcopist was informed of the VIA result.  Threshold: negative, positive minor or grade 1, positive major or grade 2, suspected invasive cancer. | Conventional cytology.  Cytologist: not reported.  Second assessment: not reported.  Blinding: blinded to HPV.  Threshold: negative, ASC-US, LSIL, HSIL | Biopsies & ECC: targeted biopsy in case of visible lesions and TZ1 or TZ2, ECC was recommended in case of TZ3. LLETZ was possible.  Histopathologist: pathologist.  Blinding: not reported.  Outcome: <CIN2, CIN2+, CIN3+. |
| **Tan, 2023^15^**  Australia, 2018-2020 | 462 | Colposcopist: not reported.  Blinding: not reported.  Threshold: no visible lesion, negative, LSIL, HSIL, glandular atypia, malignancy. | Liquid-based cytology.  Cytologist: not reported.  Second assessment: not reported.  Blinding: not reported.  Threshold: negative, ASC-US, LSIL, HSIL. | Biopsies & ECC: biopsy, ECC, excisional treatment.  Histopathologist: not reported.  Blinding: not reported.  Outcome: <CIN2, CIN2+. |
| Threshold of colposcopy results for meta-analysis: negative = negative, no visible lesions; low-grade lesions = any abnormal, low-grade, positive minor or grade 1, LSIL; high-grade lesions = high-grade, positive major or grade 2, HSIL, glandular atypia, malignancy; cancer = suspected invasive cancer.  Abbreviations: N = number of HPV+ participants included in the meta-analysis; LEEP = loop electrosurgical excision procedure; LLETZ = large loop excision of the transformation zone. | | | | |

1. Risk of bias of the included studies

**supplemental Table 6.** Adapted QUADAS-2 checklist for risk of bias assessment of the included studies.

| **Domain** | **Label** | **Signalling Question** |
| --- | --- | --- |
| Participant selection | Enrolment | Was a consecutive or random sample of participants enrolled? |
| Participant selection | Exclusions | Did the study avoid inappropriate selection criteria? |
| Index test | Test cut-off | If a threshold was used, was it pre‐specified? |
| Index test | Tests blinded | Were the index and comparator test results interpreted without knowledge of the results of the reference standard? |
| Reference test | Reference test | Is the reference standard likely to correctly classify the target condition? |
| Reference test | Reference test blinded | Were the reference standard results interpreted without knowledge of the results of the index and comparator test? |
| Reference test | Incorporation bias | Did the study avoid incorporation bias? |
| Flow and timing | Delay before reference test | Was there an appropriate interval between the index and comparator test and the reference standard? |
| Flow and timing | Partial verification bias | Did all participants receive a reference standard? |
| Flow and timing | Differential verification bias | Did all participants receive the same reference standard? |
| Flow and timing | Withdrawal explained | Were all participants included in the analysis? |
| Flow and timing | Uninterpretable test results reported | Were uninterpretable index and comparator test results reported? |
| Flow and timing | Uninterpretable reference test results reported | Were uninterpretable reference test results reported? |

**supplemental Table 7.** Risk of bias of the included studies. Risk of bias was assessed using an adapted QUADAS-2 checklist.

|  | Participant selection | | Index and comparator test | | Reference test | | | Flow and timing | | | | | |
| --- | --- | --- | --- | --- | --- | --- | --- | --- | --- | --- | --- | --- | --- |
| **Author, year (study name)** | Enrolment | Exclusions | Test cut-off | Tests blinded | Reference test correct | Reference test blinded | Incorporation bias | Delay before reference test | Partial verification | Differential verification | Withdrawal explained | Uninterpretable test results reported | Uninterpretable reference test results reported |
| U of Z/JHPIEGO, 1999 | Yes | Yes | Yes | Yes | No | No | No | Yes | Yes | No | Yes | Yes | Yes |
| Belinson, 2001 (SPOCCS I) | Yes | Yes | Yes | Unclear | Yes | Yes | Yes | Yes | Yes | Yes | Yes | Yes | Yes |
| Belinson, 2003 (SPOCCS II) | Yes | Yes | Yes | Unclear | Yes | Yes | Yes | Yes | Yes | Yes | Yes | Yes | Yes |
| Sankaranarayanan, 2004 | Yes | Yes | Yes | Unclear | No | No | No | Yes | Yes | No | Yes | Yes | Yes |
| Hovland, 2010 | Yes | Yes | Yes | Yes | Yes | Yes | Yes | Yes | Yes | Yes | Yes | Yes | Yes |
| Poli, 2018 (START-UP) | Yes | No | Yes | Unclear | Yes | Unclear | Yes | Yes | Yes | Yes | Yes | Yes | Yes |
| Luckett, 2019 | Yes | Yes | Yes | Yes | Yes | Unclear | Yes | Yes | Yes | Yes | Yes | Yes | Yes |
| Dang, 2022 | Yes | Yes | Yes | Yes | Yes | No | Yes* | Yes | Yes | Yes* | Yes | No | No |
| Luckett, 2023 | Yes | Yes | Yes | Yes | Yes | Unclear | Yes | Yes | Yes | Yes | Yes | Yes | Yes |
| Valls, 2023 (ESTAMPA) | Yes | Yes | Yes | No | Yes | No | Yes* | Yes | Yes | Yes* | Yes | Yes | Yes |
| Tan, 2023 | Yes | Yes | Yes | Unclear | Yes | Unclear | No | Yes | Yes | No | Yes | No | Yes |

‘Yes’ indicates low risk of bias; ‘No’ indicates moderate to high risk of bias; ‘Unclear’ indicates that the article did not report sufficient details to make a judgement.

* In Dang, 2022 and Valls, 2023 (ESTAMPA) women with abnormal colposcopic impression at screening had biopsy and histopathology and women with normal colposcopic impression at screening had a follow-up visit within 18 to 24 months. While these studies used the colposcopic impression to decide on the follow-up algorithm, which impacted the available reference standard (i.e. differential verification), the follow-up algorithm had a high likelihood of finding false negatives and the risk of bias due to incorporation bias and differential verification was therefore considered small. Consequently, these studies received a ‘Yes’ score in these domains.

Abbreviations: U of Z = University of Zimbabwe.

1. Diagnostic accuracy of colposcopy

Colposcopy at cut-off minor-grade lesions

Outcome: CIN2+


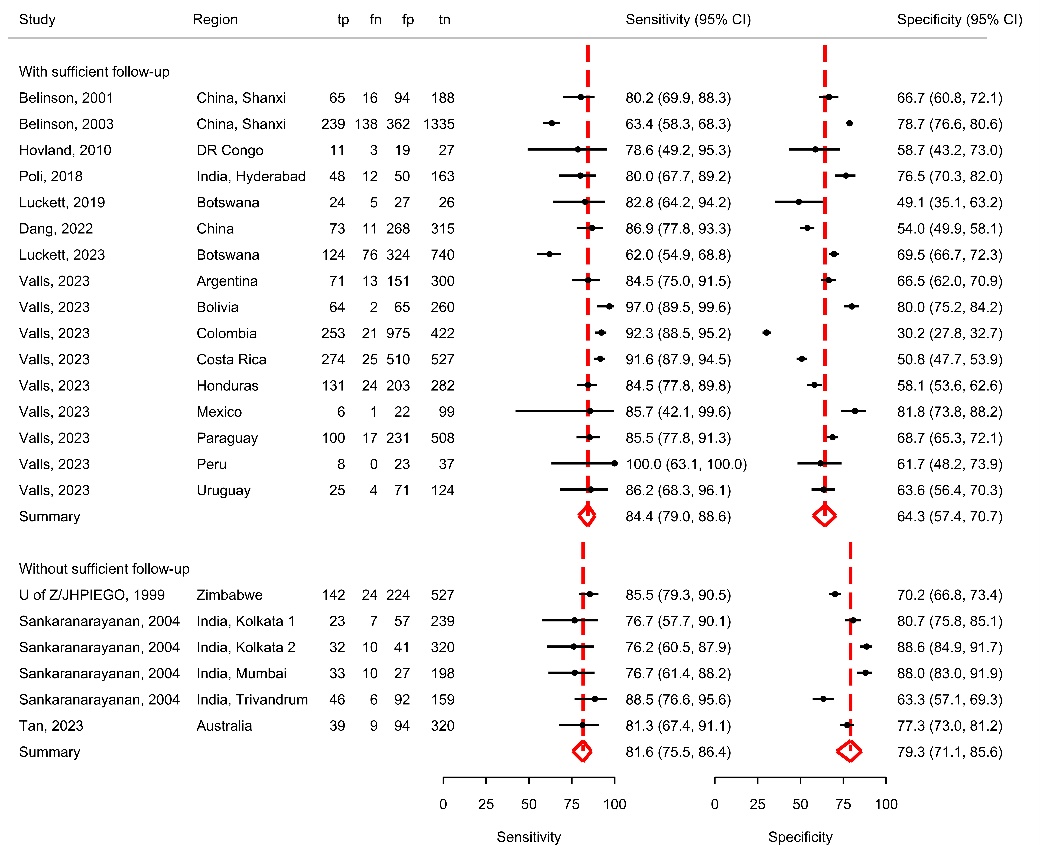


**supplemen****tal Figure 1.** Meta-analysis of the sensitivity and specificity for detection of CIN2+ in the triage of HPV-positive women using colposcopy with low-grade colposcopic impression as test cut-off. Abbreviations: tp = true positives; fn = false negatives; fp = false positives; tn = true negatives; CI = confidence interval; U of Z = University of Zimbabwe.

Complete follow-up: sensitivity: τ^2^ = 0.38; I^2^ = 57.9%

specificity: τ^2^ = 0.33; I^2^ = 92.2%

Incomplete follow-up: sensitivity: τ^2^ = 0.09; I^2^ = 37.9%

specificity: τ^2^ = 0.28; I^2^ = 93.2%

Outcome: CIN3+


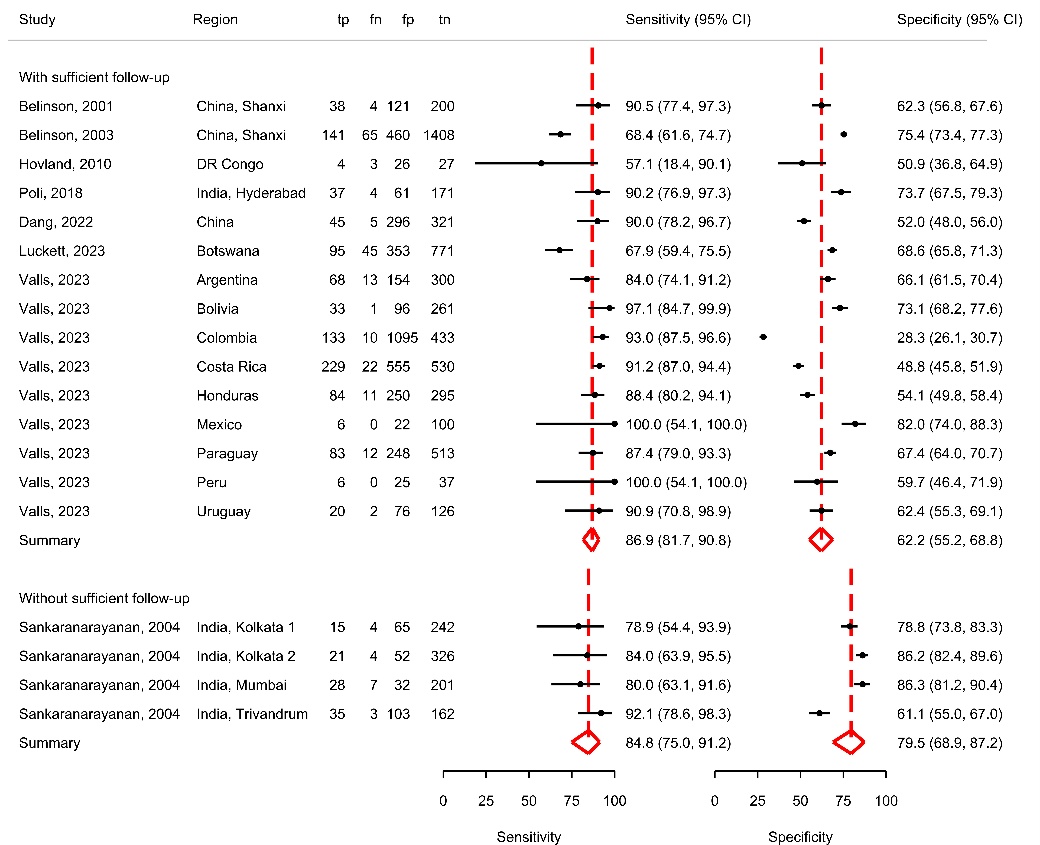


**supplemental Figure 2.** Meta-analysis of the sensitivity and specificity for detection of CIN3+ in the triage of HPV-positive women using colposcopy with low-grade colposcopic impression as test cut-off. Abbreviations: tp = true positives; fn = false negatives; fp = false positives; tn = true negatives; CI = confidence interval.

Complete follow-up: sensitivity: τ^2^ = 0.35; I^2^ = 43.5%

specificity: τ^2^ = 0.32; I^2^ = 93.7%

Incomplete follow-up: sensitivity: τ^2^ = 0.12; I^2^ = 29.4%

specificity: τ^2^ = 0.31; I^2^ = 92.8%

Colposcopy at cut-off major-grade lesions

Outcome: CIN2+


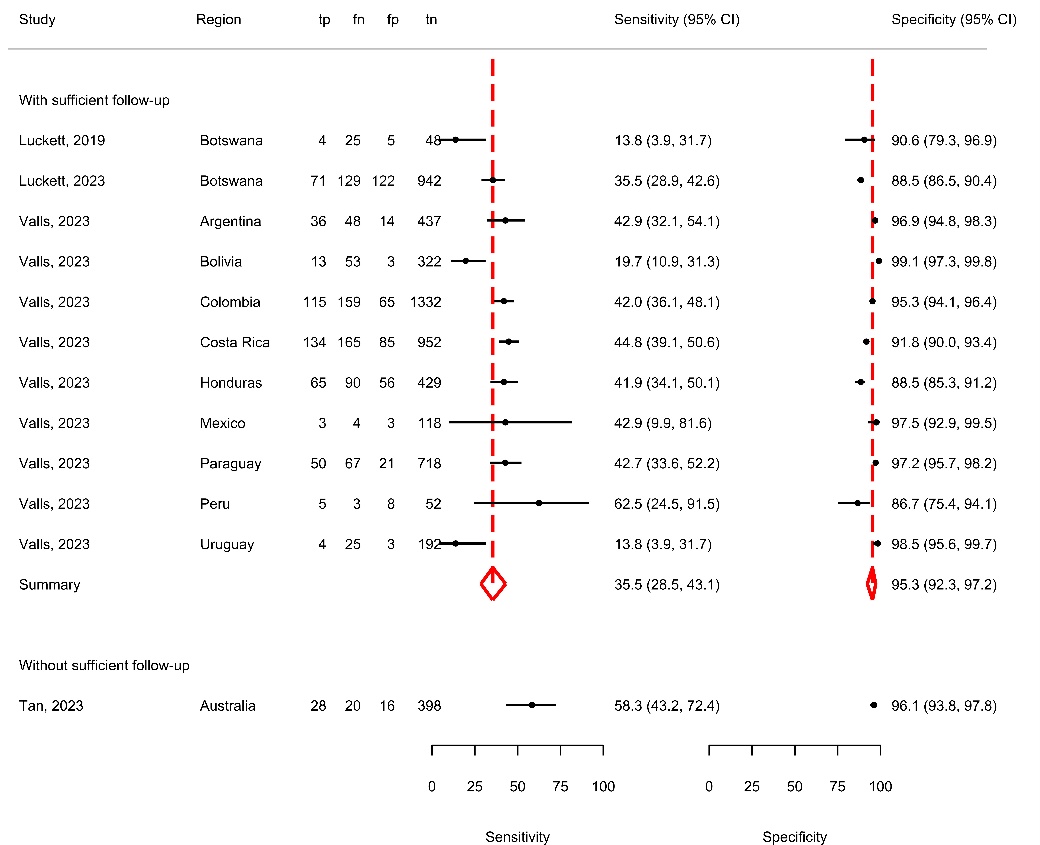


**Supplemental figure 3.** Meta-analysis of the sensitivity and specificity for detection of CIN2+ in the triage of HPV-positive women using colposcopy with high-grade colposcopic impression as test cut-off. Abbreviations: tp = true positives; fn = false negatives; fp = false positives; tn = true negatives; CI = confidence interval.

Complete follow-up: sensitivity: τ^2^ = 0.20; I^2^ = 54.5%

specificity: τ^2^ = 0.69; I^2^ = 80.3%

Outcome: CIN3+


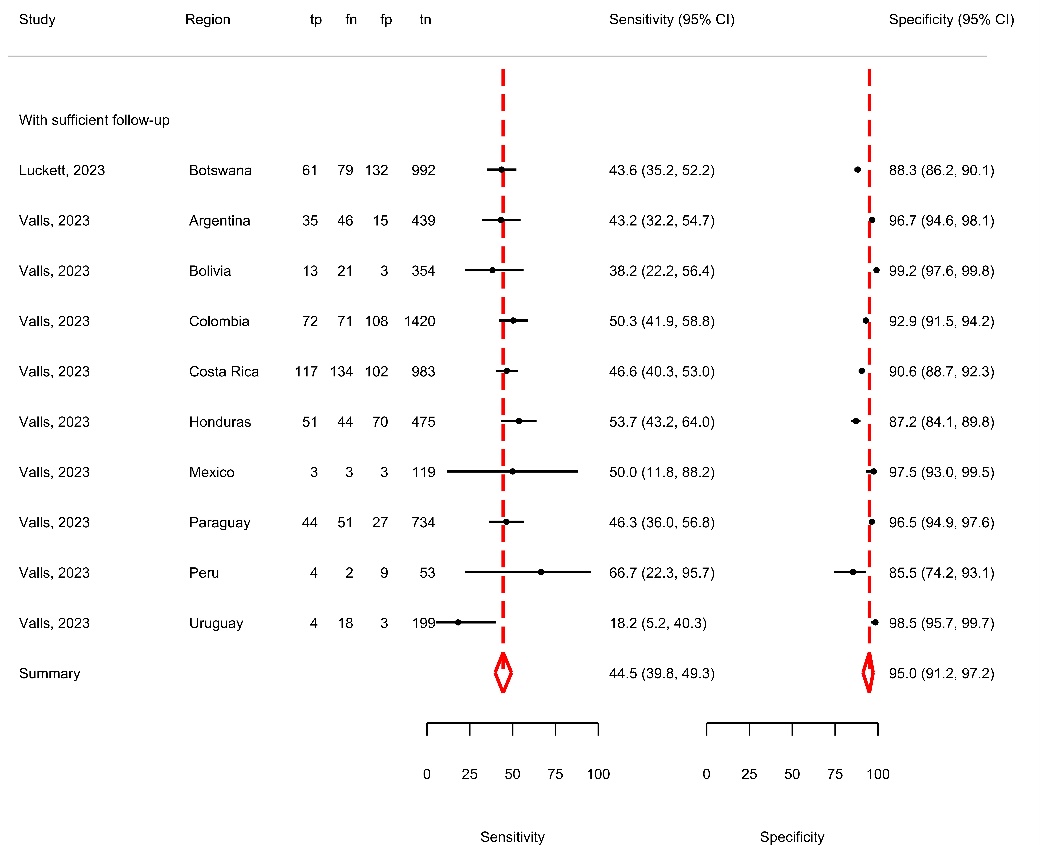


**Supplemental figure 4.** Meta-analysis of the sensitivity and specificity for detection of CIN3+ in the triage of HPV-positive women using colposcopy with high-grade colposcopic impression as test cut-off. Abbreviations: tp = true positives; fn = false negatives; fp = false positives; tn = true negatives; CI = confidence interval.

Complete follow-up: sensitivity: τ^2^ = 0.03; I^2^ = 15.7%

specificity: τ^2^ = 0.84; I^2^ = 87.1%

1. Diagnostic accuracy of cytology

Cytology at cut-off ASC-US

Outcome: CIN2+


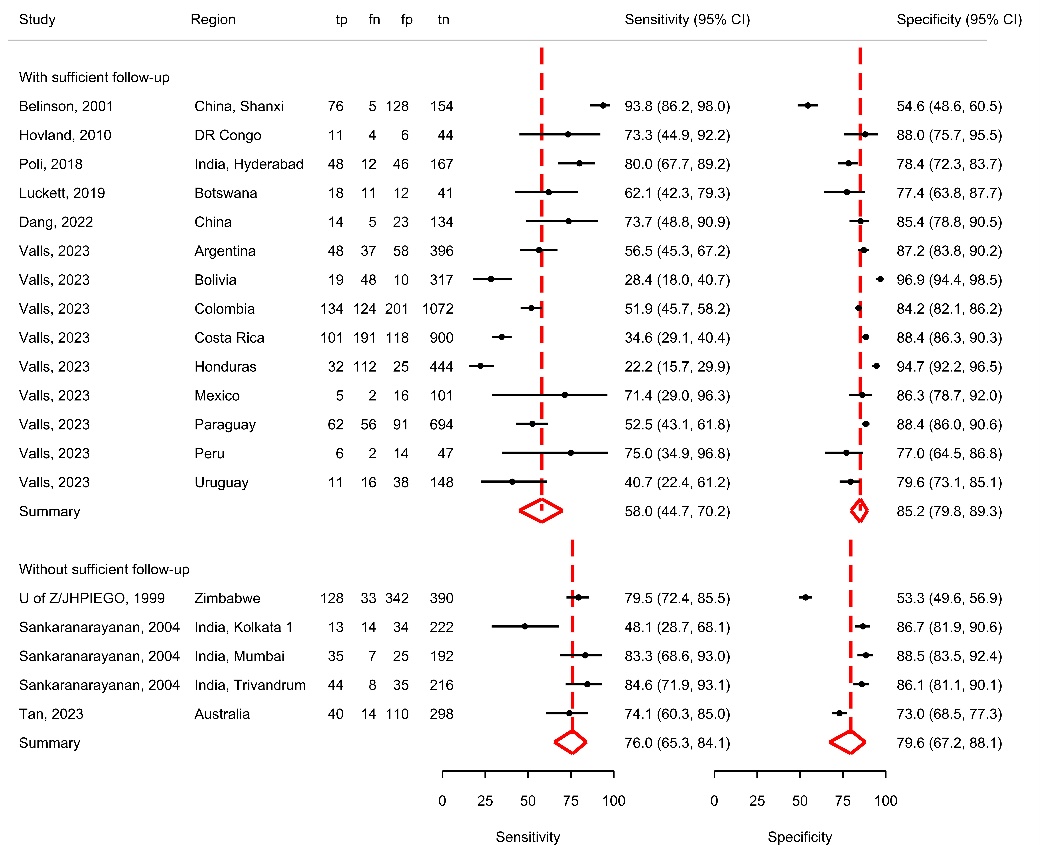


**Supplemental figure 5.** Meta-analysis of the sensitivity and specificity for detection of CIN2+ in the triage of HPV-positive women using cytology at cut-off ASC-US. Abbreviations: tp = true positives; fn = false negatives; fp = false positives; tn = true negatives; CI = confidence interval; U of Z = University of Zimbabwe.

Complete follow-up: sensitivity: τ^2^ = 0.90; I^2^ = 81.5%

specificity: τ^2^ = 0.47; I^2^ = 88.0%

Incomplete follow-up: sensitivity: τ^2^ = 0.23; I^2^ = 65.2%

specificity: τ^2^ = 0.51; I^2^ = 95.5%

Outcome: CIN3+


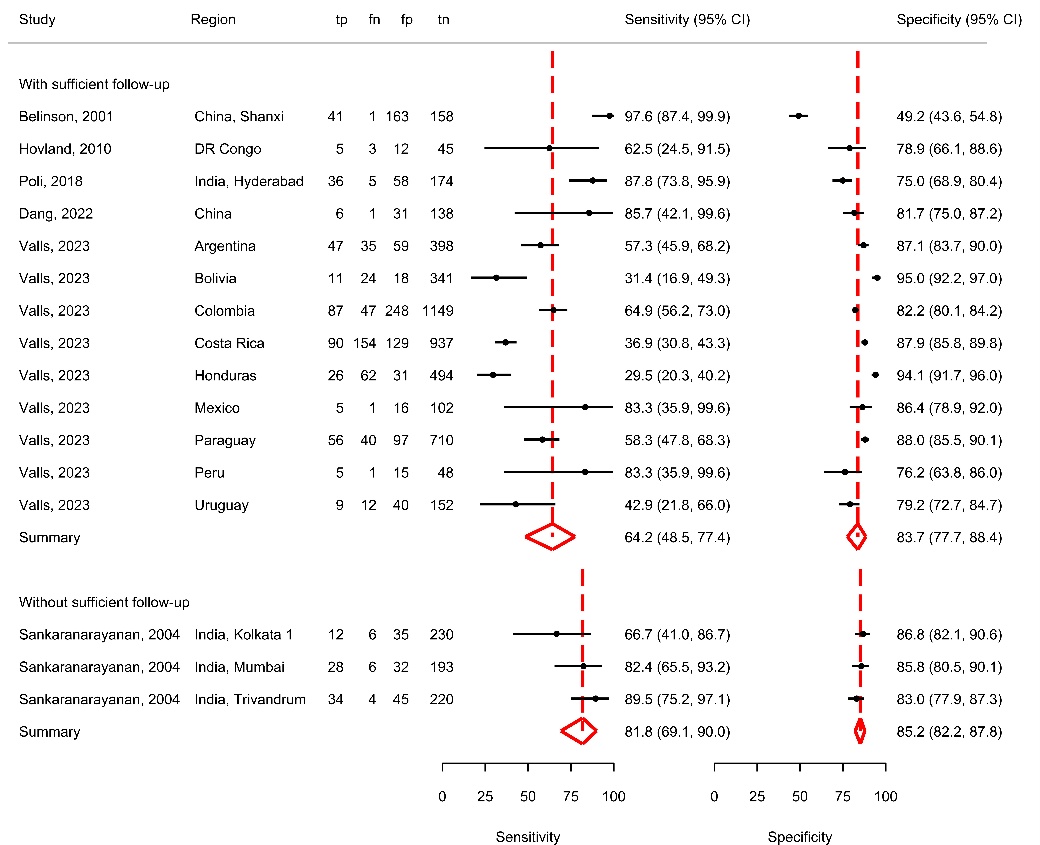


**Supplemental figure 6.** Meta-analysis of the sensitivity and specificity for detection of CIN3+ in the triage of HPV-positive women using cytology at cut-off ASC-US. Abbreviations: tp = true positives; fn = false negatives; fp = false positives; tn = true negatives; CI = confidence interval.

Complete follow-up: sensitivity: τ^2^ = 1.18; I^2^ = 76.1%

specificity: τ^2^ = 0.49; I^2^ = 91.1%

Incomplete follow-up: sensitivity: τ^2^ = 0.14; I^2^ = 35.3%

specificity: τ^2^ = 0.01; I^2^ = 18.4%

Cytology at cut-off LSIL

Outcome: CIN2+


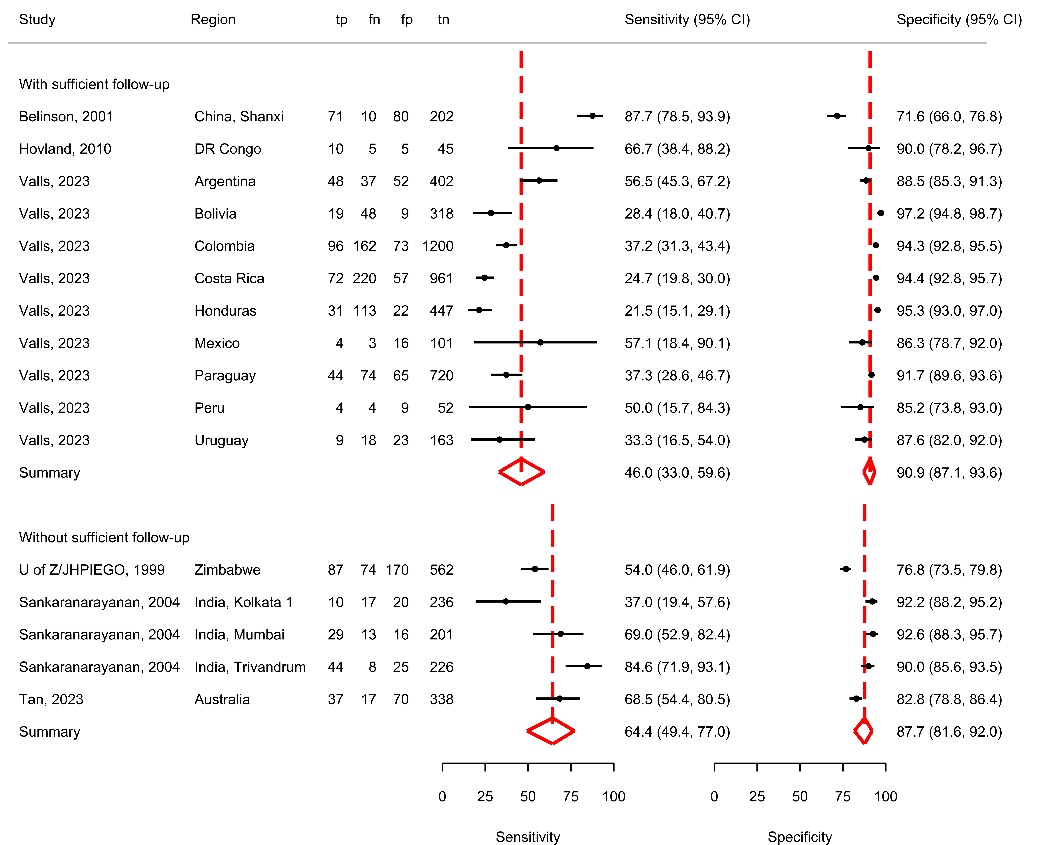


**Supplemental figure 7.** Meta-analysis of the sensitivity and specificity for detection of CIN2+ in the triage of HPV-positive women using cytology at cut-off LSIL. Abbreviations: tp = true positives; fn = false negatives; fp = false positives; tn = true negatives; CI = confidence interval; U of Z = University of Zimbabwe.

Complete follow-up: sensitivity: τ^2^ = 0.75; I^2^ = 79.4%

specificity: τ^2^ = 0.39; I^2^ = 82.3%

Incomplete follow-up: sensitivity: τ^2^ = 0.39; I^2^ = 79.4%

specificity: τ^2^ = 0.26; I^2^ = 88.5%

Outcome: CIN3+


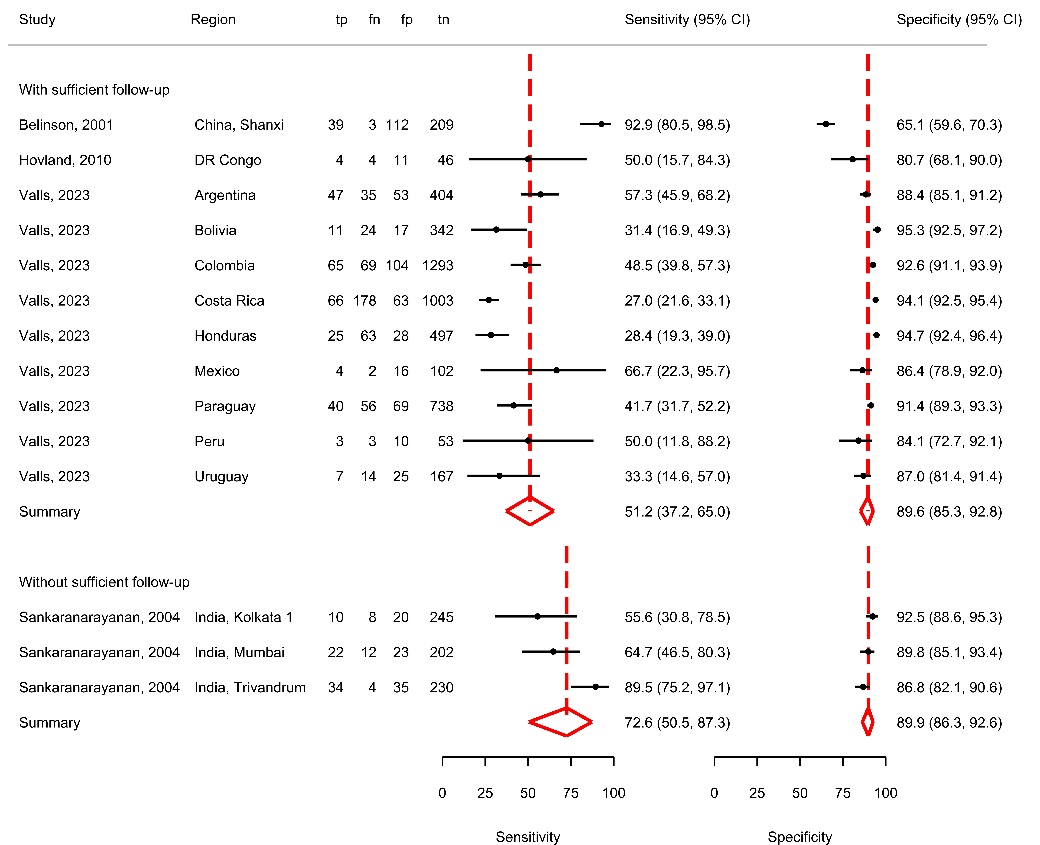


**Supplemental figure 8.** Meta-analysis of the sensitivity and specificity for detection of CIN3+ in the triage of HPV-positive women using cytology at cut-off LSIL. Abbreviations: tp = true positives; fn = false negatives; fp = false positives; tn = true negatives; CI = confidence interval.

Complete follow-up: sensitivity: τ^2^ = 0.78; I^2^ = 74.2%

specificity: τ^2^ = 0.41; I^2^ = 85.4%

Incomplete follow-up: sensitivity: τ^2^ = 0.52; I^2^ = 70.1%

specificity: τ^2^ = 0.05; I^2^ = 50.8%

Cytology at cut-off HSIL

Outcome: CIN2+


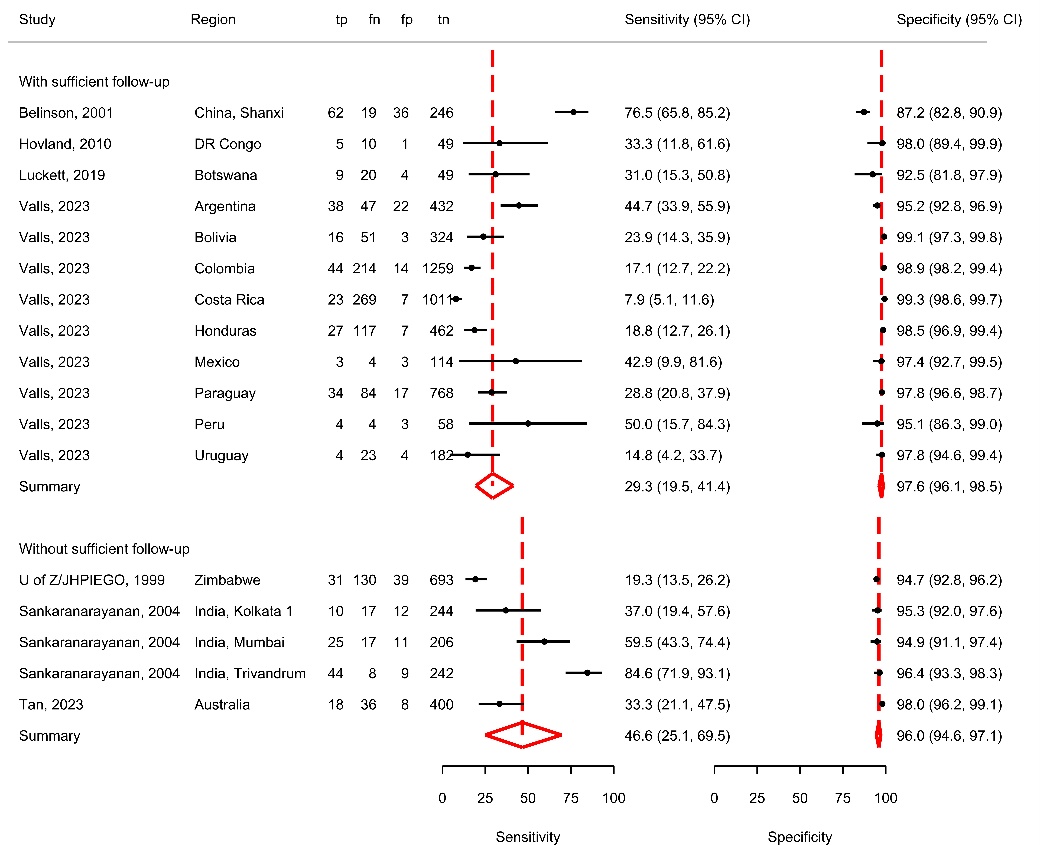


**Supplemental figure 9.** Meta-analysis of the sensitivity and specificity for detection of CIN2+ in the triage of HPV-positive women using cytology at cut-off HSIL. Abbreviations: tp = true positives; fn = false negatives; fp = false positives; tn = true negatives; CI = confidence interval; U of Z = University of Zimbabwe.

Complete follow-up: sensitivity: τ^2^ = 0.77; I^2^ = 76.2%

specificity: τ^2^ = 0.64; I^2^ = 61.4%

Incomplete follow-up: sensitivity: τ^2^ = 1.09; I^2^ = 90.5%

specificity: τ^2^ = 0.05; I^2^ = 37.6%

Outcome: CIN3+


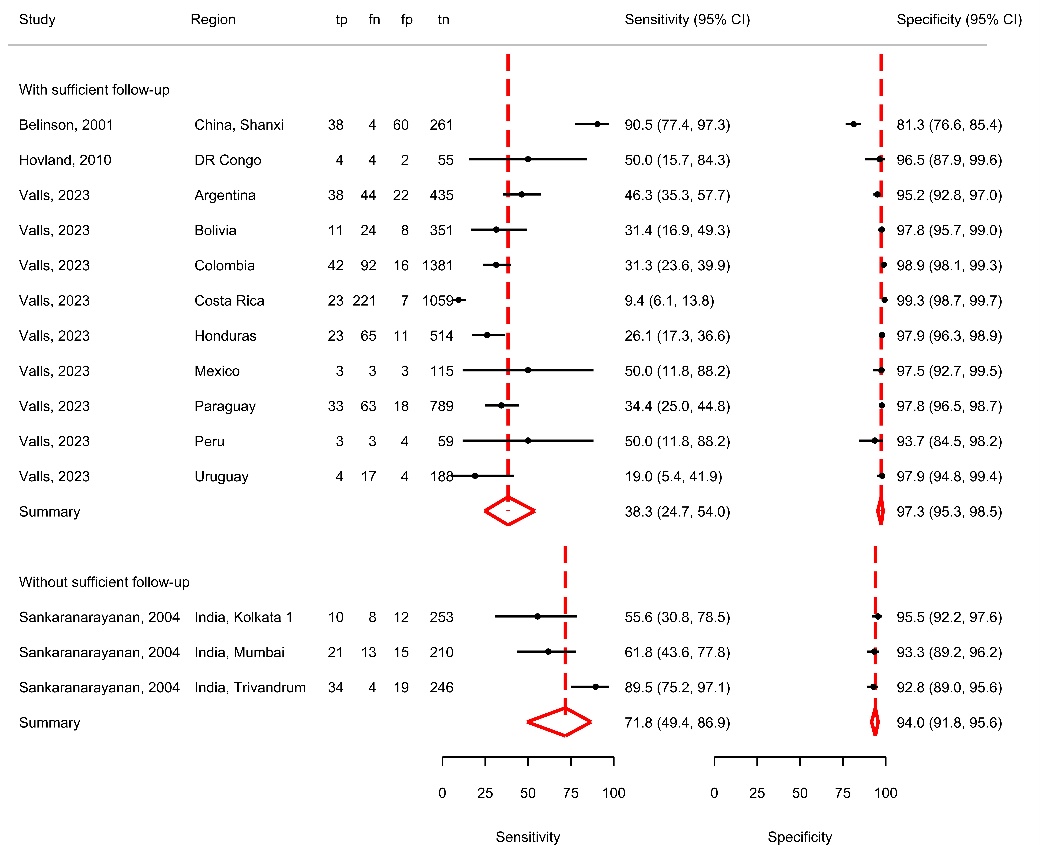


**Supplemental figure 10.** Meta-analysis of the sensitivity and specificity for detection of CIN3+ in the triage of HPV-positive women using cytology at cut-off HSIL. Abbreviations: tp = true positives; fn = false negatives; fp = false positives; tn = true negatives; CI = confidence interval.

Complete follow-up: sensitivity: τ^2^ = 1.00; I^2^ = 76.3%

specificity: τ^2^ = 0.82; I^2^ = 72.5%

Incomplete follow-up: sensitivity: τ^2^ = 0.52; I^2^ = 70.1%

specificity: τ^2^ = 0.02; I^2^ = 20.2%

1. Relative diagnostic accuracy of colposcopy compared to cytology

Index: colposcopy at cut-off low-grade colposcopic impression

Comparator: cytology at cut-off ASC-US

Outcome: CIN2+


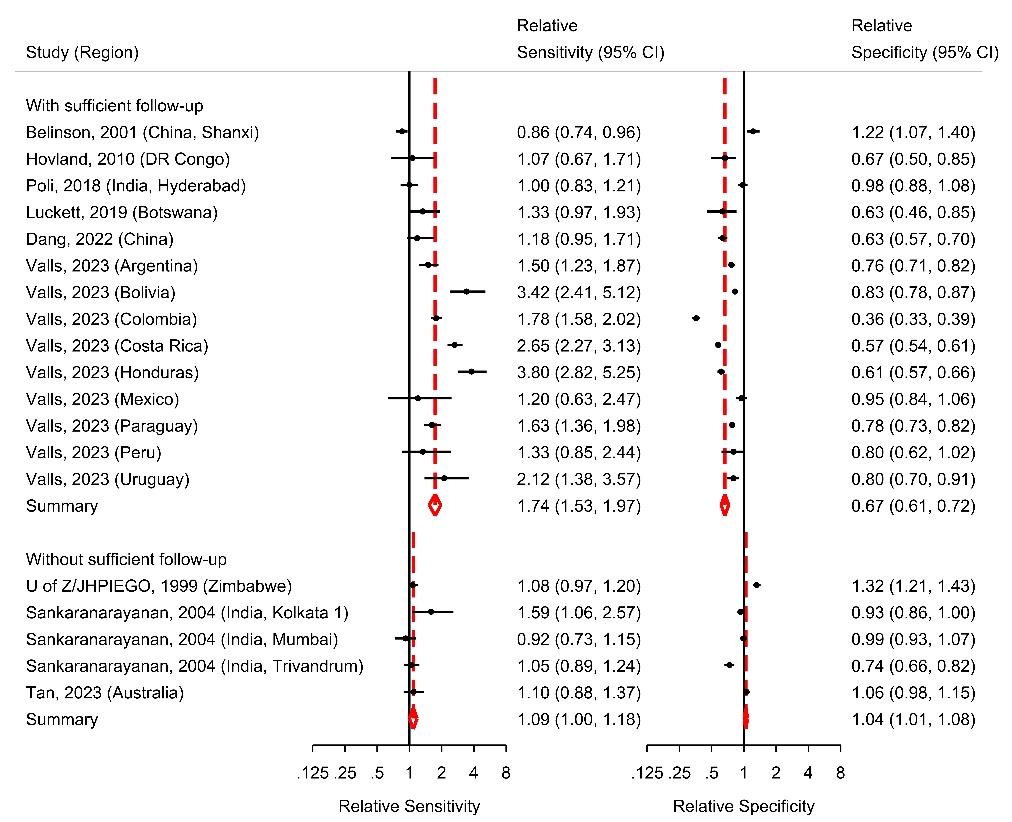


**Supplemental figure 11.** Meta-analysis of the relative sensitivity and specificity for detection of CIN2+ in the triage of HPV-positive women using colposcopy with low-grade colposcopic impression as test cut-off compared to cytology with ASC-US as test cut-off. Abbreviations: CI = confidence interval; U of Z = University of Zimbabwe.

Complete follow-up: sensitivity: τ^2^ = 0.25; specificity: τ^2^ = 0.24.

Incomplete follow-up: sensitivity: τ^2^ = 0.09; specificity: τ^2^ = 0.26.

Outcome: CIN3+


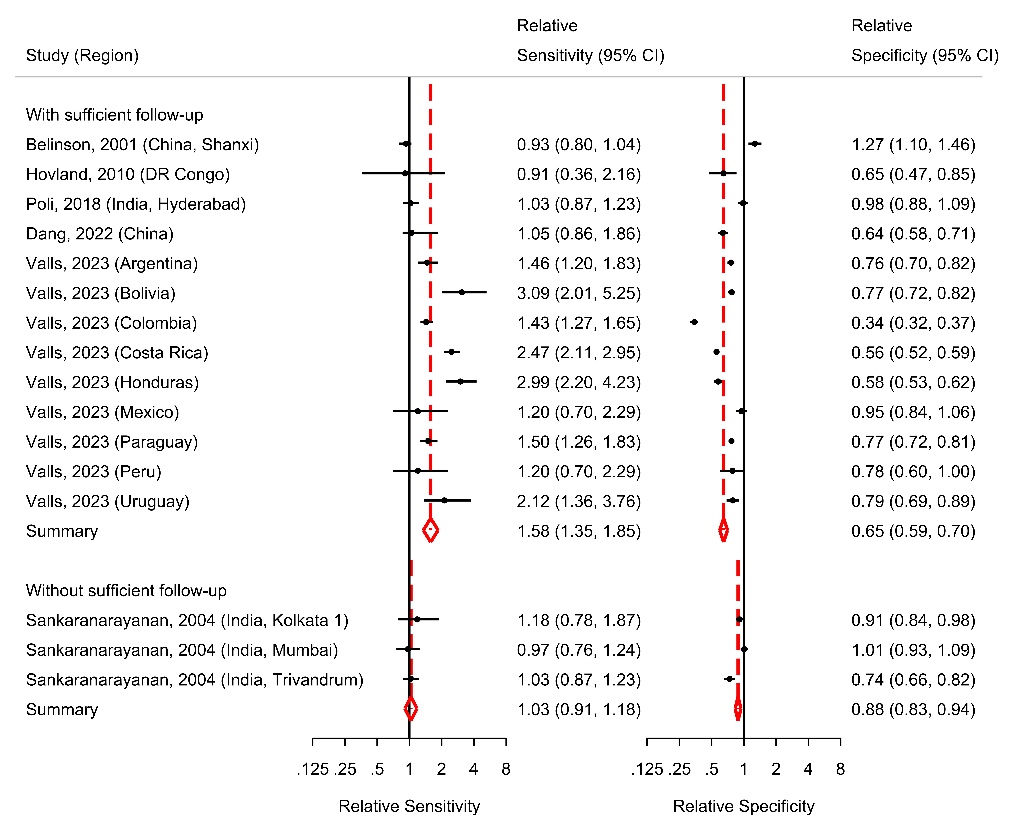


**Supplemental figure 12.** Meta-analysis of the relative sensitivity and specificity for detection of CIN3+ in the triage of HPV-positive women using colposcopy with low-grade colposcopic impression as test cut-off compared to cytology with ASC-US as test cut-off. Abbreviations: CI = confidence interval.

Complete follow-up: sensitivity: τ^2^ = 0.51; specificity: τ^2^ = 0.23.

Incomplete follow-up: sensitivity: τ^2^ = 0.16; specificity: τ^2^ = 0.13.

Index: colposcopy at cut-off low-grade colposcopic impression

Comparator: cytology at cut-off LSIL

Outcome: CIN2+


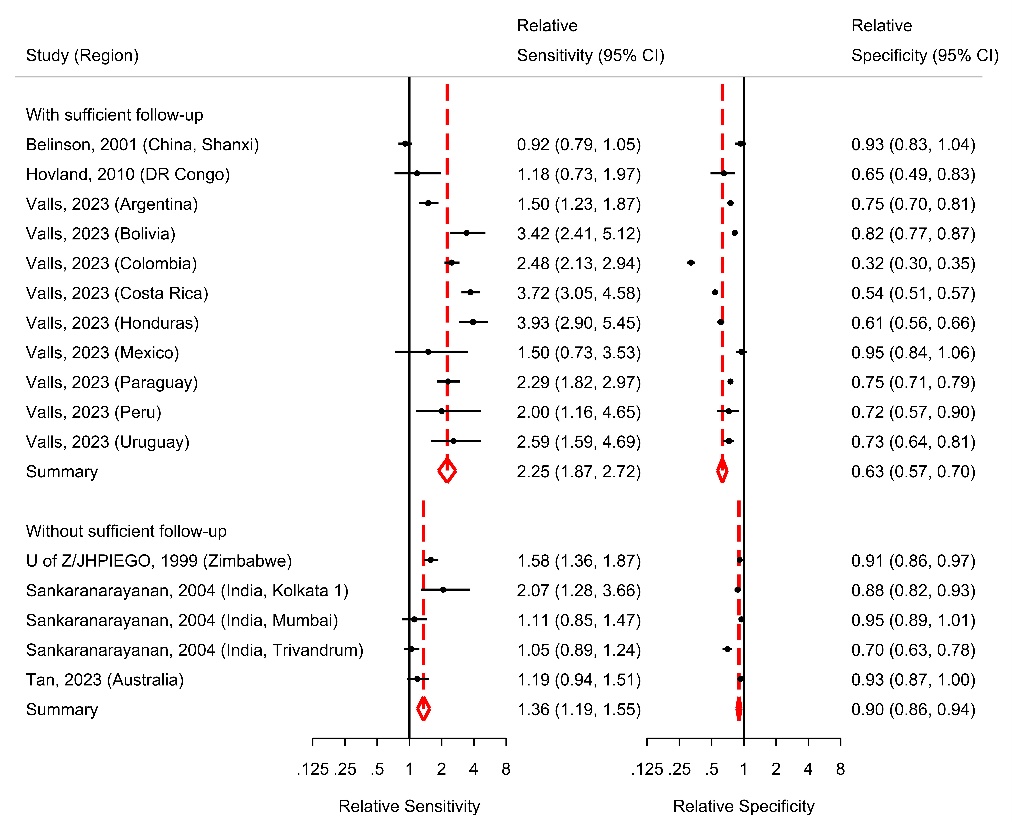


**Supplemental figure 13.** Meta-analysis of the relative sensitivity and specificity for detection of CIN2+ in the triage of HPV-positive women using colposcopy with low-grade colposcopic impression as test cut-off compared to cytology with LSIL as test cut-off. Abbreviations: CI = confidence interval; U of Z = University of Zimbabwe.

Complete follow-up: sensitivity: τ^2^ = 0.27; specificity: τ^2^ = 0.22.

Incomplete follow-up: sensitivity: τ^2^ = 0.20; specificity: τ^2^ = 0.18.

Outcome: CIN3+


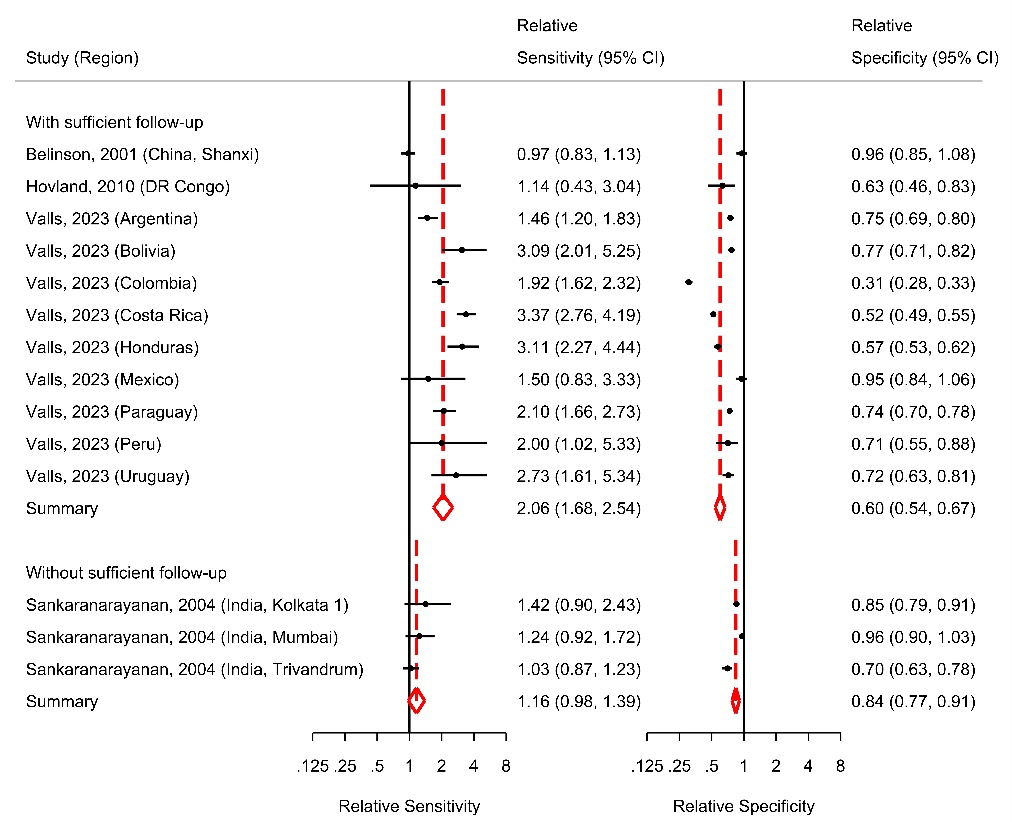


**Supplemental figure 14.** Meta-analysis of the relative sensitivity and specificity for detection of CIN3+ in the triage of HPV-positive women using colposcopy with low-grade colposcopic impression as test cut-off compared to cytology with LSIL as test cut-off. Abbreviations: CI = confidence interval.

Complete follow-up: sensitivity: τ^2^ = 0.37; specificity: τ^2^ = 0.22.

Incomplete follow-up: sensitivity: τ^2^ = 0.38; specificity: τ^2^ = 0.17.

Index: colposcopy at cut-off low-grade colposcopic impression

Comparator: cytology at cut-off HSIL

Outcome: CIN2+


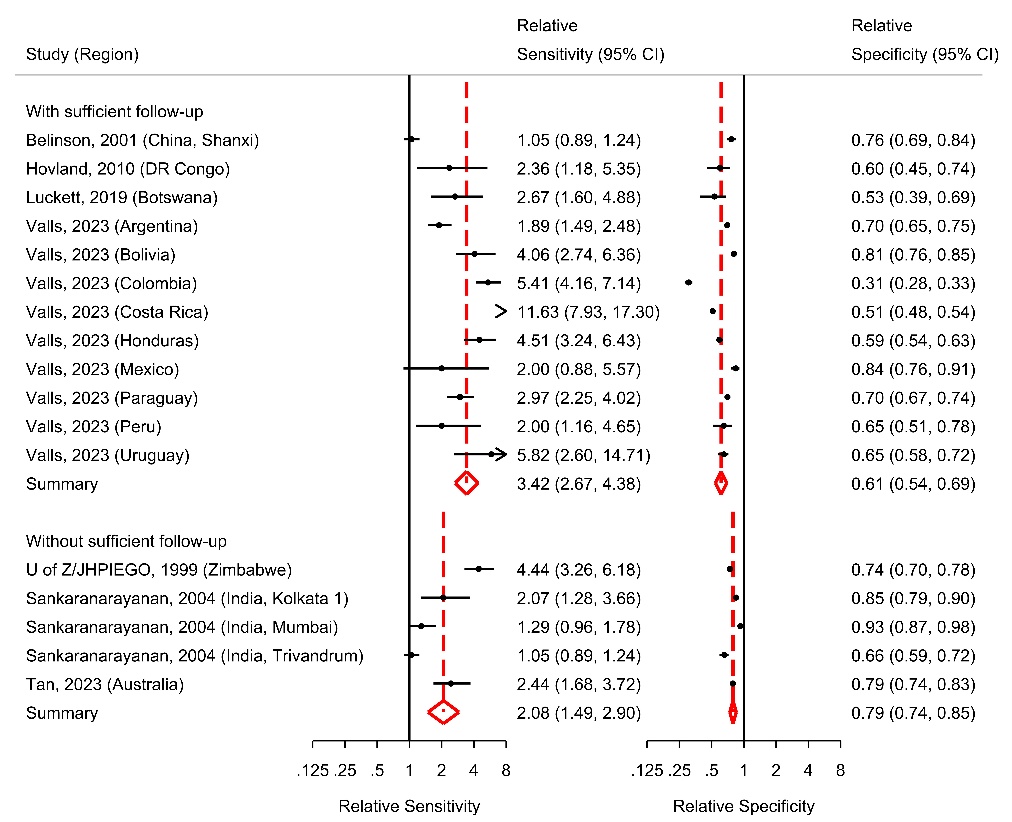


**Supplemental figure 15.** Meta-analysis of the relative sensitivity and specificity for detection of CIN2+ in the triage of HPV-positive women using colposcopy with low-grade colposcopic impression as test cut-off compared to cytology with HSIL as test cut-off. Abbreviations: CI = confidence interval; U of Z = University of Zimbabwe.

Complete follow-up: sensitivity: τ^2^ = 0.32; specificity: τ^2^ = 0.29.

Incomplete follow-up: sensitivity: τ^2^ = 0.56; specificity: τ^2^ = 0.13.

Outcome: CIN3+


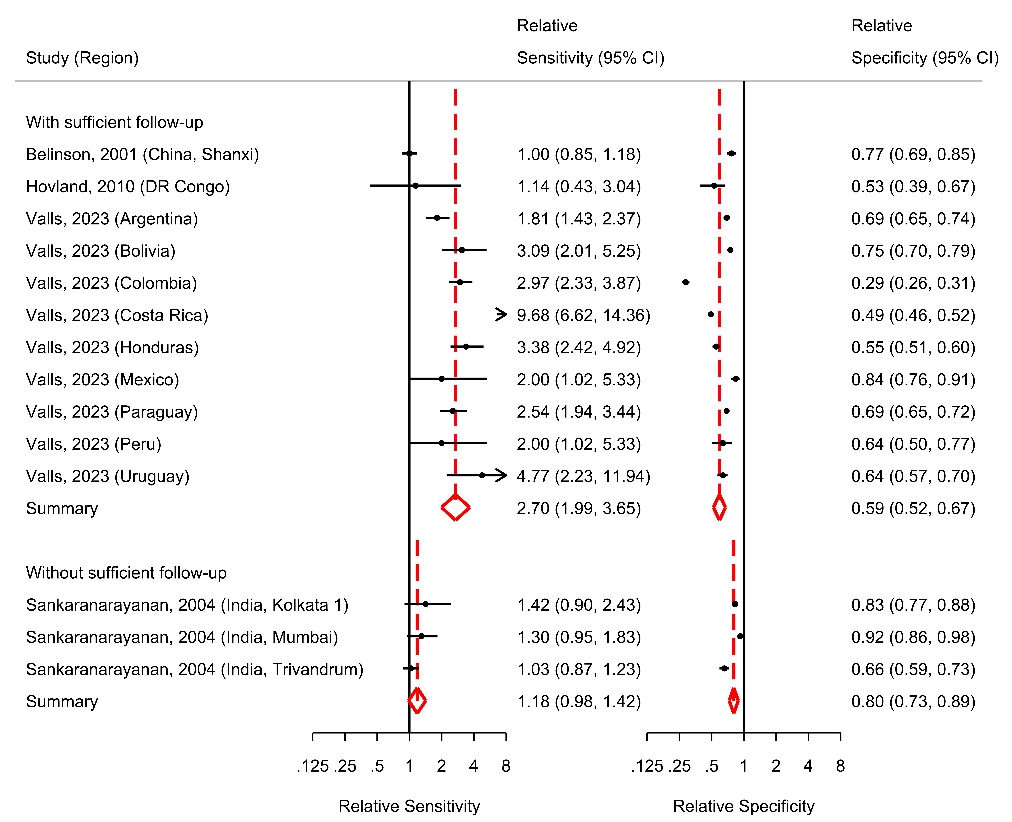


**Supplemental figure 16.** Meta-analysis of the relative sensitivity and specificity for detection of CIN3+ in the triage of HPV-positive women using colposcopy with low-grade colposcopic impression as test cut-off compared to cytology with HSIL as test cut-off. Abbreviations: CI = confidence interval.

Complete follow-up: sensitivity: τ^2^ = 0.56; specificity: τ^2^ = 0.28.

Incomplete follow-up: sensitivity: τ^2^ = 0.40; specificity: τ^2^ = 0.18.

Index: colposcopy at cut-off high-grade colposcopic impression

Comparator: cytology at cut-off ASC-US

Outcome: CIN2+


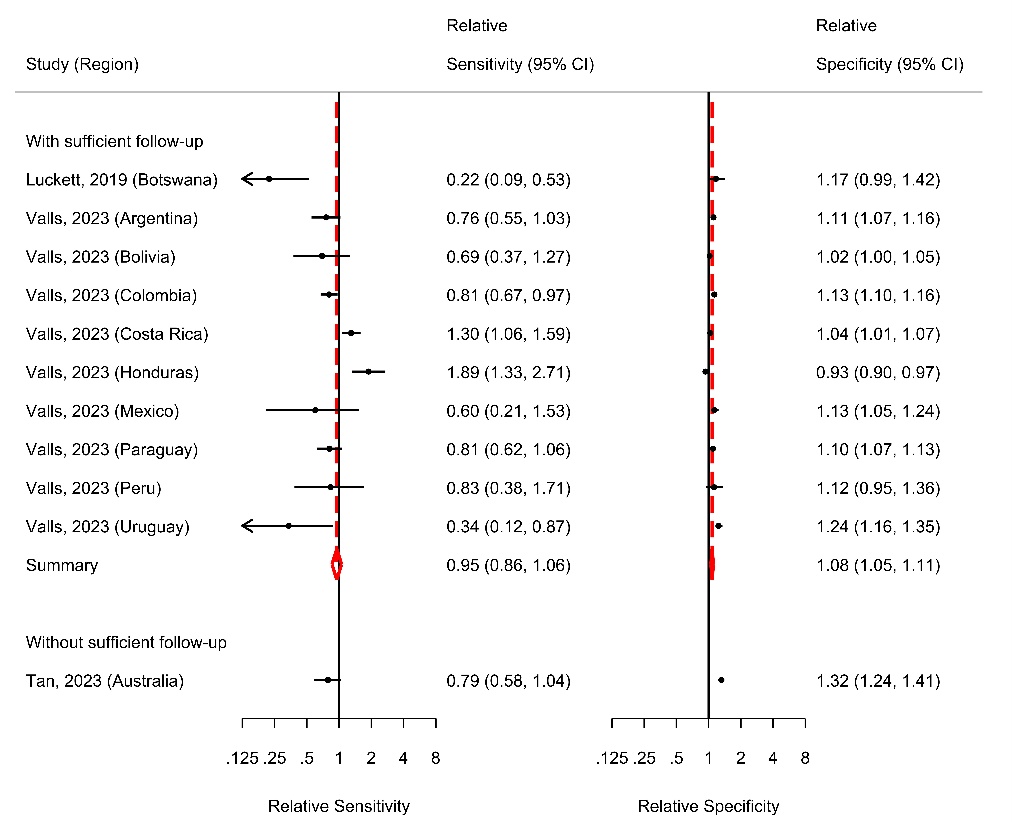


**Supplemental figure 17.** Meta-analysis of the relative sensitivity and specificity for detection of CIN2+ in the triage of HPV-positive women using colposcopy with high-grade colposcopic impression as test cut-off compared to cytology with ASC-US as test cut-off. Abbreviations: CI = confidence interval.

Complete follow-up: sensitivity: τ^2^ = 0.16; specificity: τ^2^ = 0.29.

Outcome: CIN3+


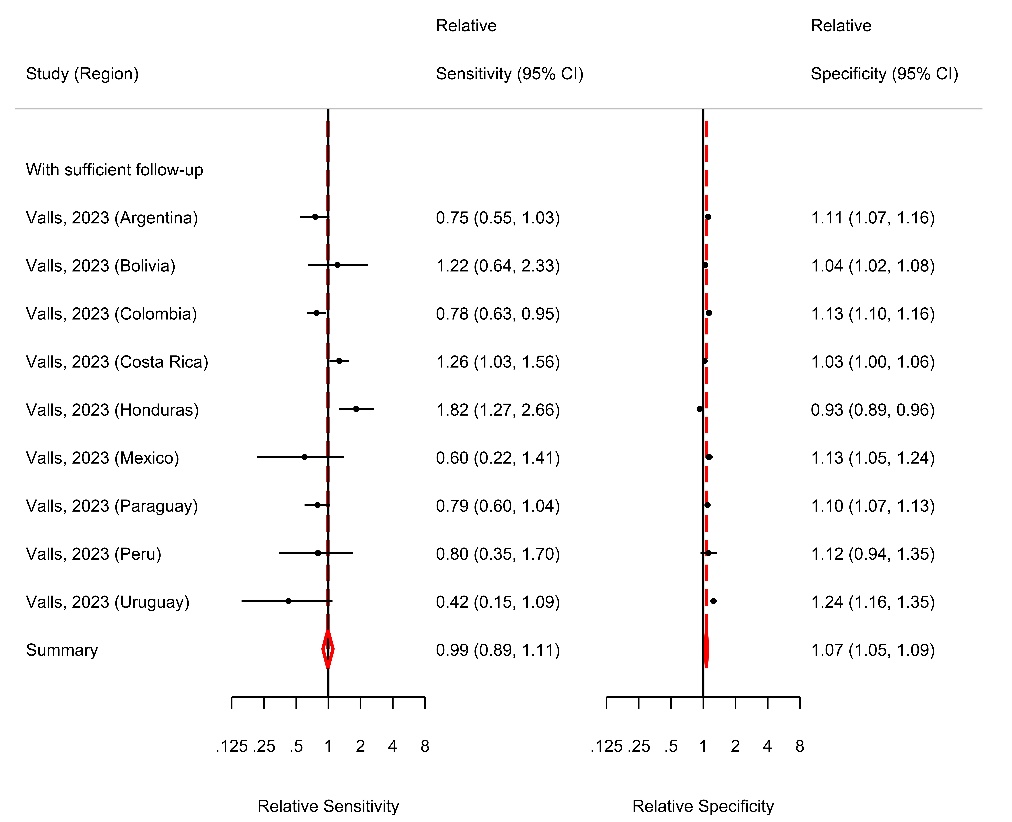


**Supplemental figure 18.** Meta-analysis of the relative sensitivity and specificity for detection of CIN3+ in the triage of HPV-positive women using colposcopy with high-grade colposcopic impression as test cut-off compared to cytology with ASC-US as test cut-off. Abbreviations: CI = confidence interval.

Complete follow-up: sensitivity: τ^2^ = 0.12; specificity: τ^2^ = 0.22.

Index: colposcopy at cut-off high-grade colposcopic impression

Comparator: cytology at cut-off LSIL

Outcome: CIN2+


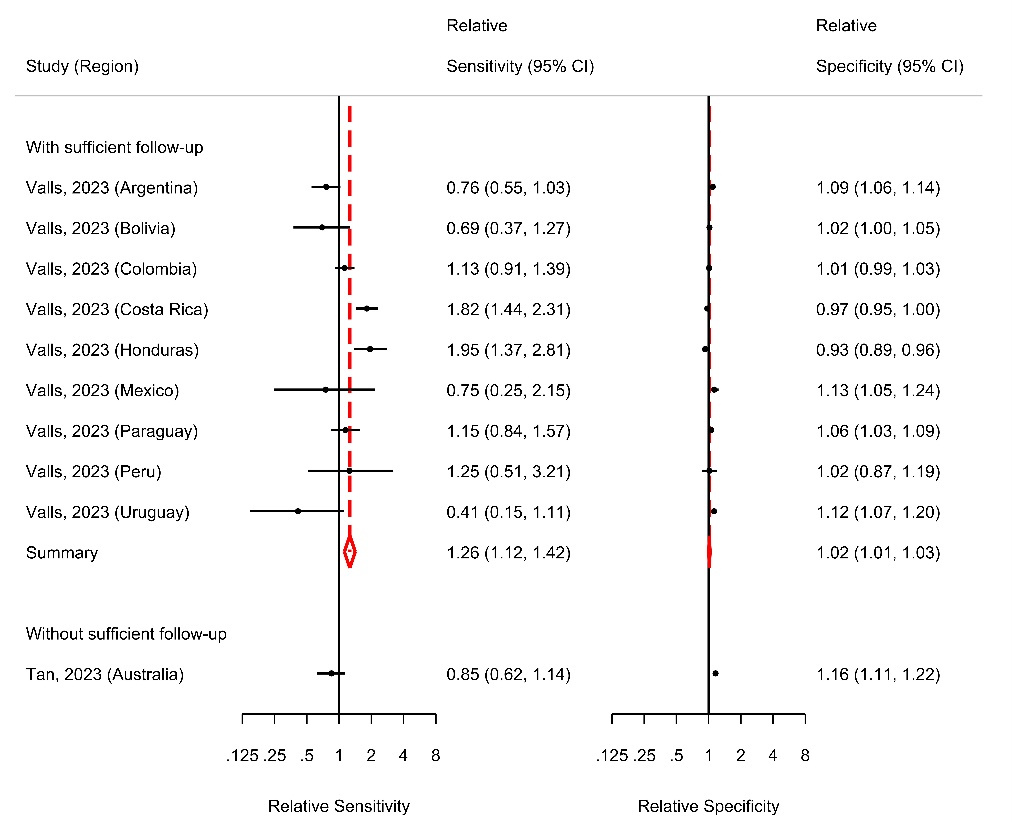


**Supplemental figure 19.** Meta-analysis of the relative sensitivity and specificity for detection of CIN2+ in the triage of HPV-positive women using colposcopy with high-grade colposcopic impression as test cut-off compared to cytology with LSIL as test cut-off. Abbreviations: CI = confidence interval.

Complete follow-up: sensitivity: τ^2^ = 0.13; specificity: τ^2^ = 0.20.

Outcome: CIN3+


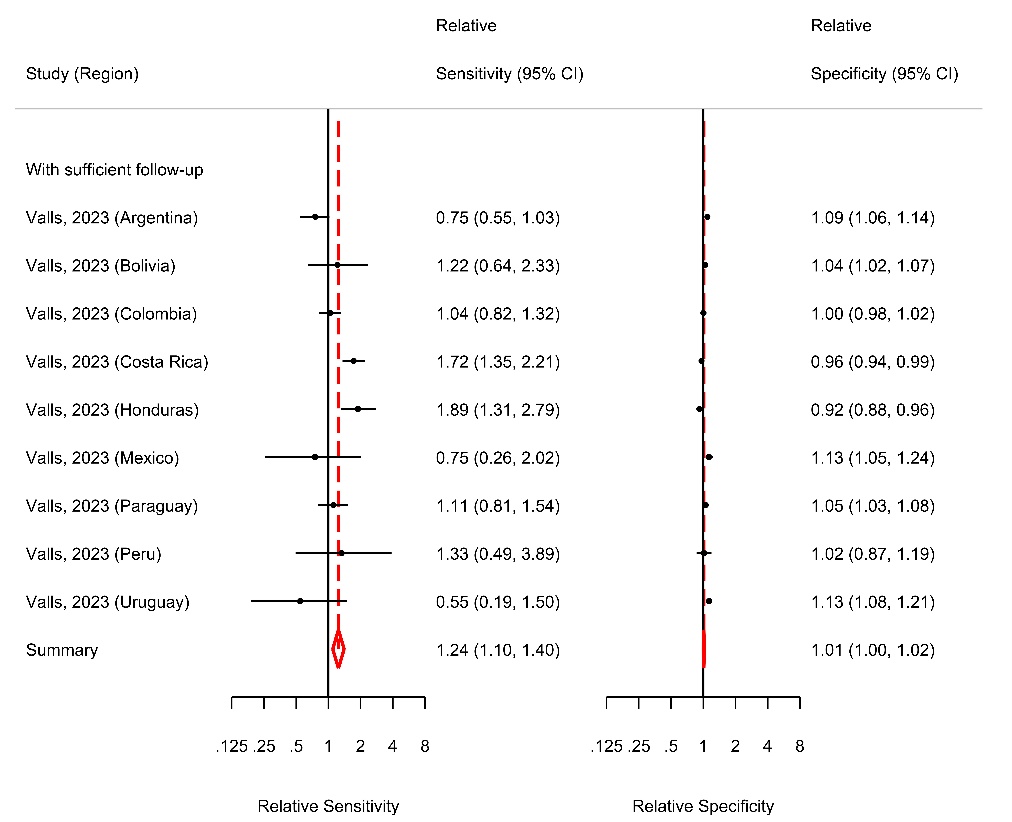


**Supplemental figure 20.** Meta-analysis of the relative sensitivity and specificity for detection of CIN3+ in the triage of HPV-positive women using colposcopy with high-grade colposcopic impression as test cut-off compared to cytology with LSIL as test cut-off. Abbreviations: CI = confidence interval.

Complete follow-up: sensitivity: τ^2^ = 0.07; specificity: τ^2^ = 0.13.

Index: colposcopy at cut-off high-grade colposcopic impression

Comparator: cytology at cut-off HSIL

Outcome: CIN2+


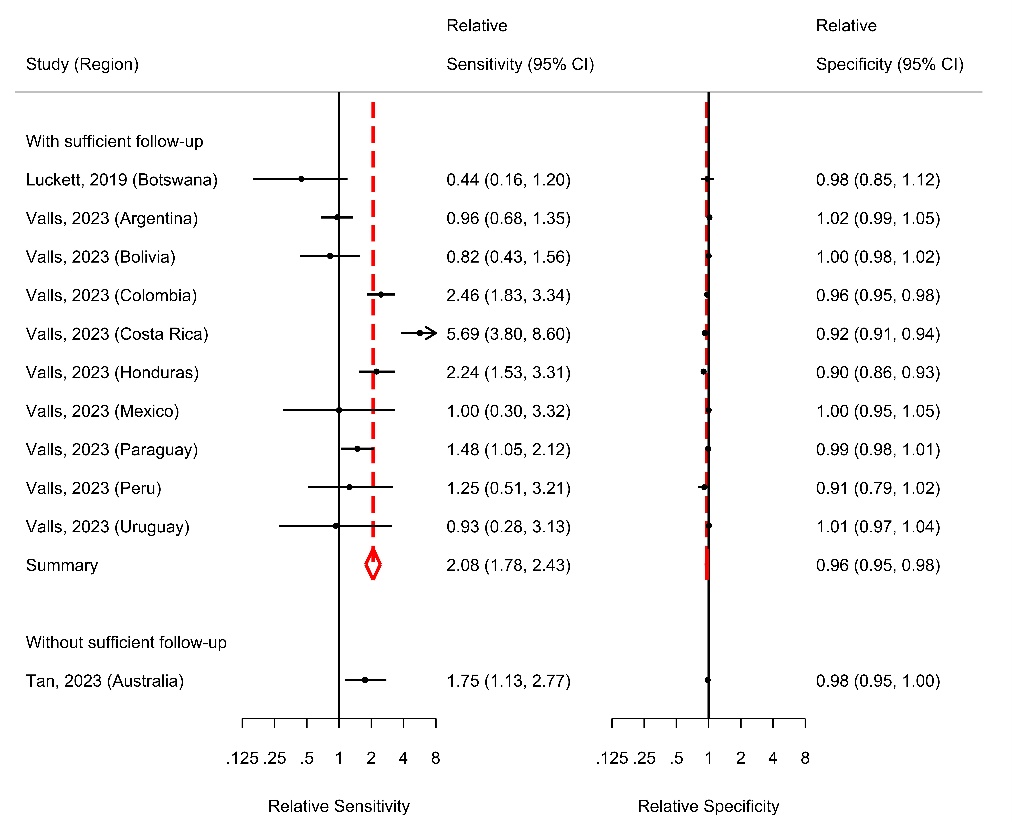


**Supplemental figure 21.** Meta-analysis of the relative sensitivity and specificity for detection of CIN2+ in the triage of HPV-positive women using colposcopy with high-grade colposcopic impression as test cut-off compared to cytology with HSIL as test cut-off. Abbreviations: CI = confidence interval.

Complete follow-up: sensitivity: τ^2^ = 0.17; specificity: τ^2^ = 0.37.

Outcome: CIN3+


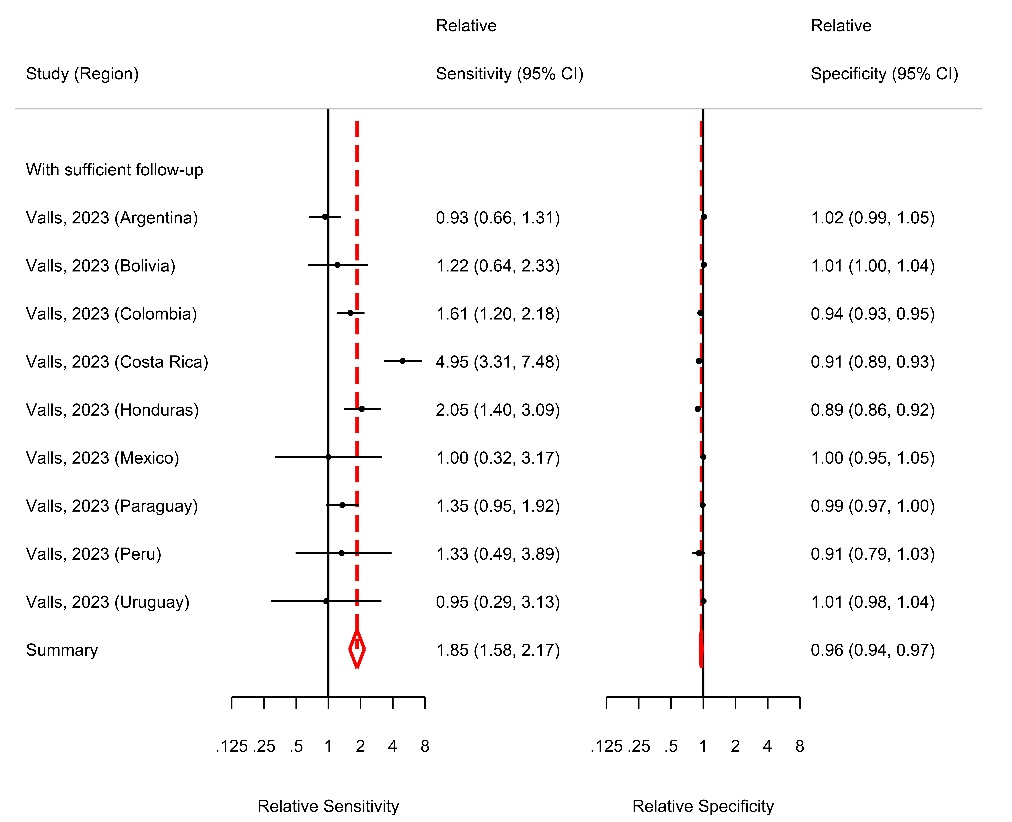


**Supplemental figure 22.** Meta-analysis of the relative sensitivity and specificity for detection of CIN3+ in the triage of HPV-positive women using colposcopy with high-grade colposcopic impression as test cut-off compared to cytology with HSIL as test cut-off. Abbreviations: CI = confidence interval.

Complete follow-up: sensitivity: τ^2^ = 0.11; specificity: τ^2^ = 0.32.

1. SROC curves


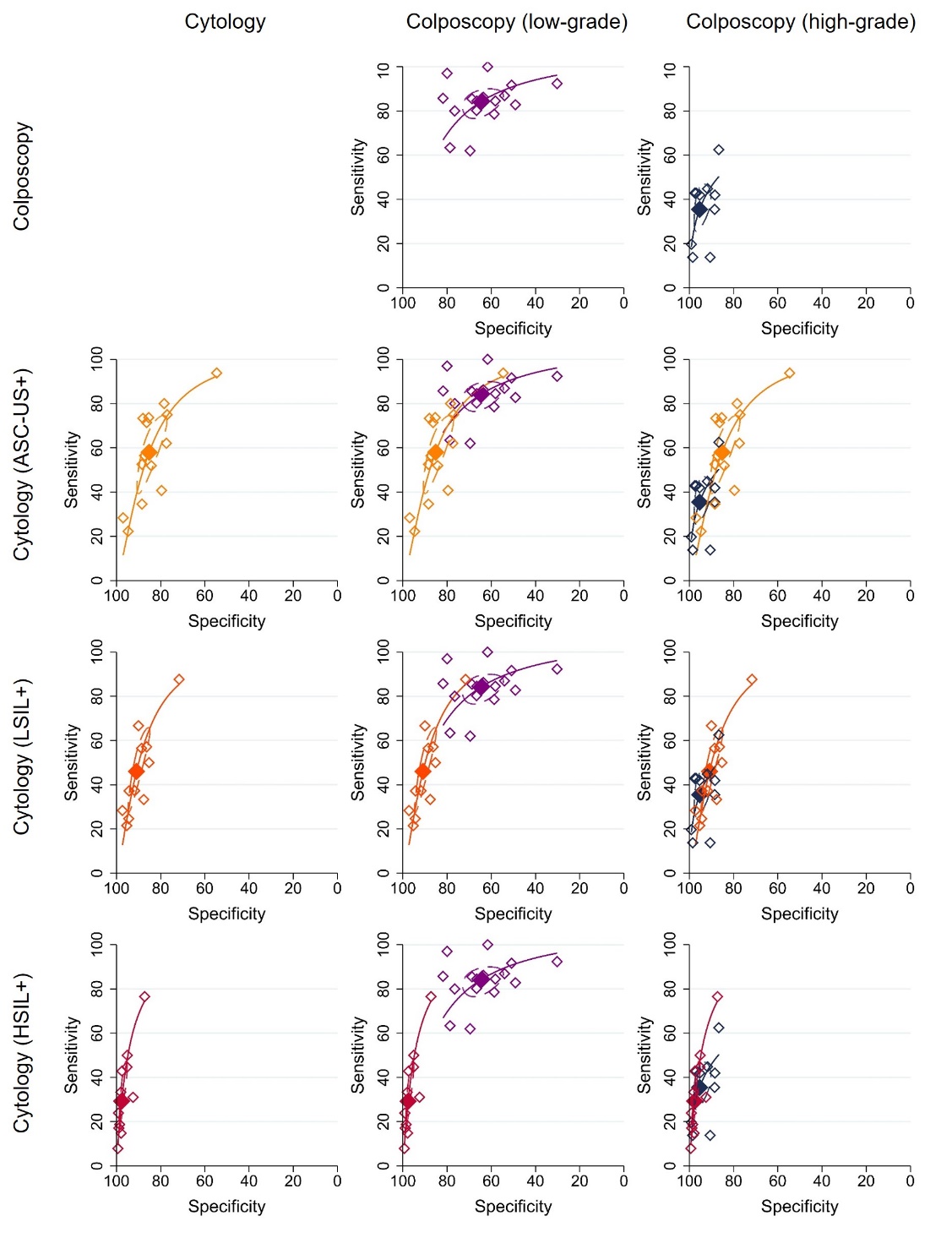


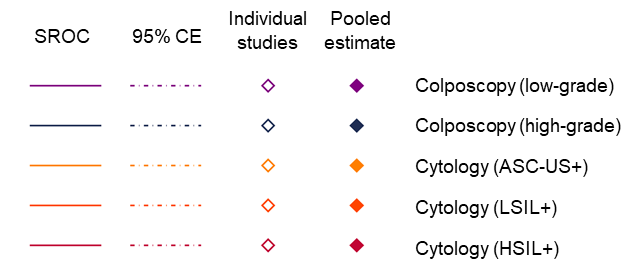


**Supplemental figure 23.** SROC curve plotting sensitivity against specificity for detection of CIN2+ in the triage of HPV-positive women using colposcopy with low- or high-grade colposcopic impression as test cut-off or using cytology with ASC-US, LSIL or HSIL as test cut-off. Studies with incomplete follow-up were excluded. Abbreviations: SROC = Summary Receiver Operating Characteristic Curve; CE = confidence ellipse.


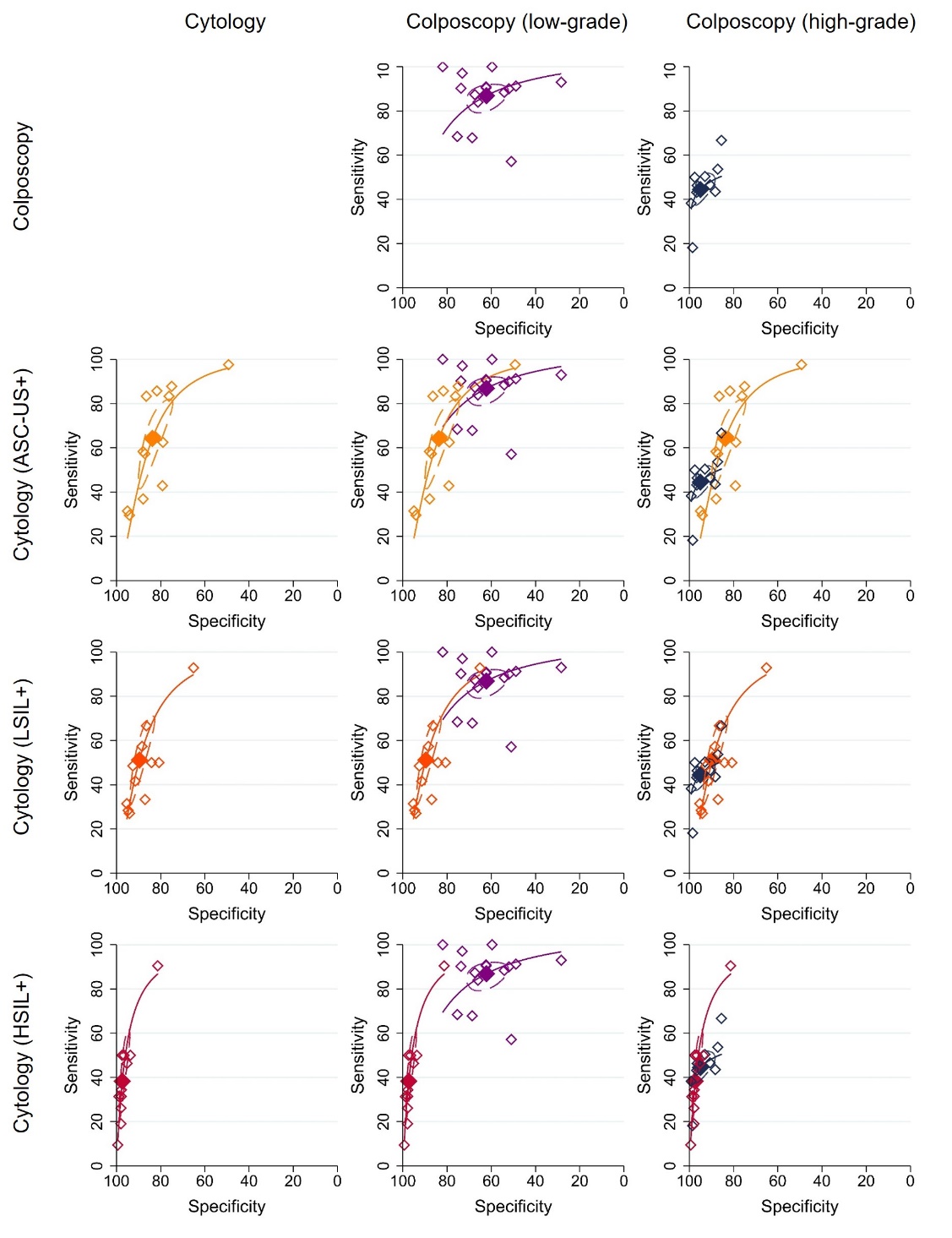


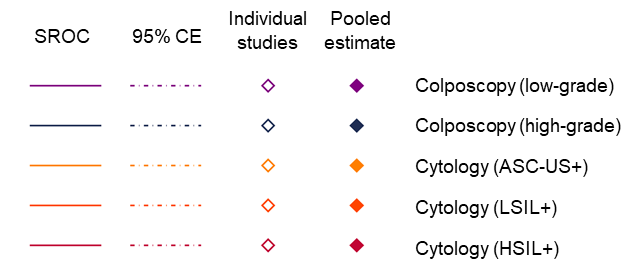


**Supplemental figure 24.** SROC curve plotting sensitivity against specificity for detection of CIN3+ in the triage of HPV-positive women using colposcopy with low- or high-grade colposcopic impression as test cut-off or using cytology with ASC-US, LSIL or HSIL as test cut-off. Studies with incomplete follow-up were excluded. Abbreviations: SROC = Summary Receiver Operating Characteristic Curve; CE = confidence ellipse.

1. Sensitivity analysis

**supplemental table 8.** Leave-one-out sensitivity analysis for the meta-analysis of sensitivity and specificity for detection of CIN2+ in the triage of HPV-positive women using colposcopy with low-grade colposcopic impression as test cut-off, based on studies with complete follow-up.

|  | **Estimate (95% CI)** | | **Relative to main analysis*** | |
| --- | --- | --- | --- | --- |
| **Omitted study** | **Sensitivity** | **Specificity** | **Sensitivity** | **Specificity** |
| None  (main analysis) | 84·4 (79.0 – 88.6) | 64·3 (57.4 – 70.7) | - | - |
| Belinson, 2001 | 84·8 (79·0 – 89·1) | 64·1 (56·7 – 70·9) | 1·00 | 1·00 |
| Belinson, 2003 | 85·7 (80·9 – 89·5) | 63·1 (56·0 – 69·7) | 1·02 | 0·98 |
| Hoveland, 2010 | 84·6 (78·9 – 88·9) | 64·6 (57·3 – 71·2) | 1·00 | 1·00 |
| Poli, 2018 | 84·7 (79·0 – 89·1) | 63·4 (56·2 – 70·0) | 1·00 | 0·99 |
| Luckett, 2019 | 84·4 (78·7 – 88·8) | 65·1 (58·0 – 71·6) | 1·00 | 1·01 |
| Dang, 2022 | 84·3 (78·4 – 88·8) | 65·0 (57·8 – 71·6) | 1·00 | 1·01 |
| Luckett, 2023 | 85·5 (80·9 – 89·2) | 63·9 (56·6 – 70·7) | 1·01 | 0·99 |
| Valls, 2023 | 76·7 (67·9 – 83·7) | 66·2 (58·3 – 73·2) | 0·91 | 1·03 |

* calculated as (sensitivity/specificity) from leave-one-out meta-analysis divided by (sensitivity/specificity) from primary analysis (ratio). Values = 1 indicate a similar value compared to the main analysis, > 1 a higher value, < 1 a lower value.

Abbreviations: CI = confidence interval.

**supplemental table 9.** Leave-one-out sensitivity analysis for the meta-analysis of the relative sensitivity and specificity for detection of CIN2+ in the triage of HPV-positive women using colposcopy with low-grade colposcopic impression as test cut-off compared to cytology with ASC-US as test cut-off, based on studies with complete follow-up.

|  | **Estimate (95% CI)** | | **Relative to main analysis*** | |
| --- | --- | --- | --- | --- |
| **Omitted study** | **Relative Sensitivity** | **Relative Specificity** | **Relative Sensitivity** | **Relative Specificity** |
| None  (main analysis) | 1·74 (1·53 – 1·97) | 0·67 (0·61 – 0·72) | - | - |
| Belinson, 2001 | 1·88 (1·67 – 2·13) | 0·65 (0·60 – 0·71) | 1·08 | 0·98 |
| Hoveland, 2010 | 1·76 (1·54 – 2·00) | 0·67 (0·61 – 0·73) | 1·01 | 1·00 |
| Poli, 2018 | 1·82 (1·59 – 2·08) | 0·65 (0·60 – 0·71) | 1·05 | 0·98 |
| Luckett, 2019 | 1·75 (1·53 – 2·00) | 0·67 (0·62 – 0·73) | 1·01 | 1·01 |
| Dang, 2022 | 1·75 (1·53 – 2·01) | 0·67 (0·61 – 0·73) | 1·01 | 1·00 |
| Valls, 2023 | 1·00 (0·92 – 1·09) | 0·87 (0·82 – 0·94) | 0·58 | 1·31 |

* calculated as relative (sensitivity/specificity) from leave-one-out meta-analysis divided by relative (sensitivity/specificity) from primary analysis (ratio). Values = 1 indicate a similar value compared to the main analysis, > 1 a higher value, < 1 a lower value.

Abbreviations: CI = confidence interval.

1. Sample size effects

Sample size effects were assessed by test, test cut-off and outcome using Deeks’ regression test (supplemental table 10). Deeks’ funnel plots were created when the p-value from Deeks’ test was < 0·10 (supplemental figure 25 to supplemental figure 28). Deeks’ evaluation of sample size effects is based on the log of the diagnostic odds ratio (lnDOR) and the inverse of the root of the effective sample size (1/ESS^1/2^), and provides robust funnel plots and regression tests for detecting sample size effects in systematic reviews of diagnostic test accuracy.^16^

**Calculation of the effective sample size and diagnostic odds ratio**

Effective sample size = ( 4 n_1_ n_2_ ) / ( n_1_ n_2_ )

where n_1_ = the number of diseased

n_2_ = the number of non-diseased

The effect sample size (ESS) increases when the total number of participants increases and when disease prevalence is closer to 0·5 (equal distribution of diseased and non-diseased).

Diagnostic odds ratio = ( tp / fn ) / ( fp / tn )

where tp = the number of true positives

fn = the number of false negatives

fp = the number of false positives

tn = the number of true negatives

The diagnostic odds ratio (DOR) increases when the sensitivity or specificity increases (i.e. when the number of fn or fp is relatively small).

When the number of false negatives or false positives was equal to 0, it was set to 0·5 to calculate the diagnostic odds ratio and effective sample size (continuity correction).

**supplemental table 10.** p-values from Deeks’ regression test (* when p < 0·05). We did not adjust for multiplicity.

| Triage test (cut-off) | CIN2+ | CIN3+ |
| --- | --- | --- |
| Colposcopy (low-grade) | 0·049 * | 0·019 * |
| Colposcopy (high-grade) | 0·64 | 0·16 |
| Cytology (ASC-US) | 0·024 * | 0·042 * |
| Cytology (LSIL) | 0·19 | 0·38 |
| Cytology (HSIL) | 0·37 | 0·51 |

The Deeks’ funnel plots show a trend where on average records with a low ESS have a higher DOR. We observed that records with a small sample size sometimes have few to no false negatives (Almonte, 2020 sites in Mexico and Peru). In contrast Hovland, 2010 and Luckett, 2019 also have a small sample size and report a higher number of false negatives. This is likely the consequence of the higher CIN2+ / CIN3+ prevalence reported in Hovland, 2010 and Luckett, 2019. Both of these studies included a high number of women living with HIV who are at higher risk of HPV infection and CIN2+ / CIN3+.

It is possible that studies and study sites with a small sample size and / or taking place in areas with low to moderate CIN2+ / CIN3+ prevalence are more likely to underestimate the number of false negatives, leading to higher DORs. While we cannot exclude the presence of publication bias, there are other possible explanations for the observed sample size effects.


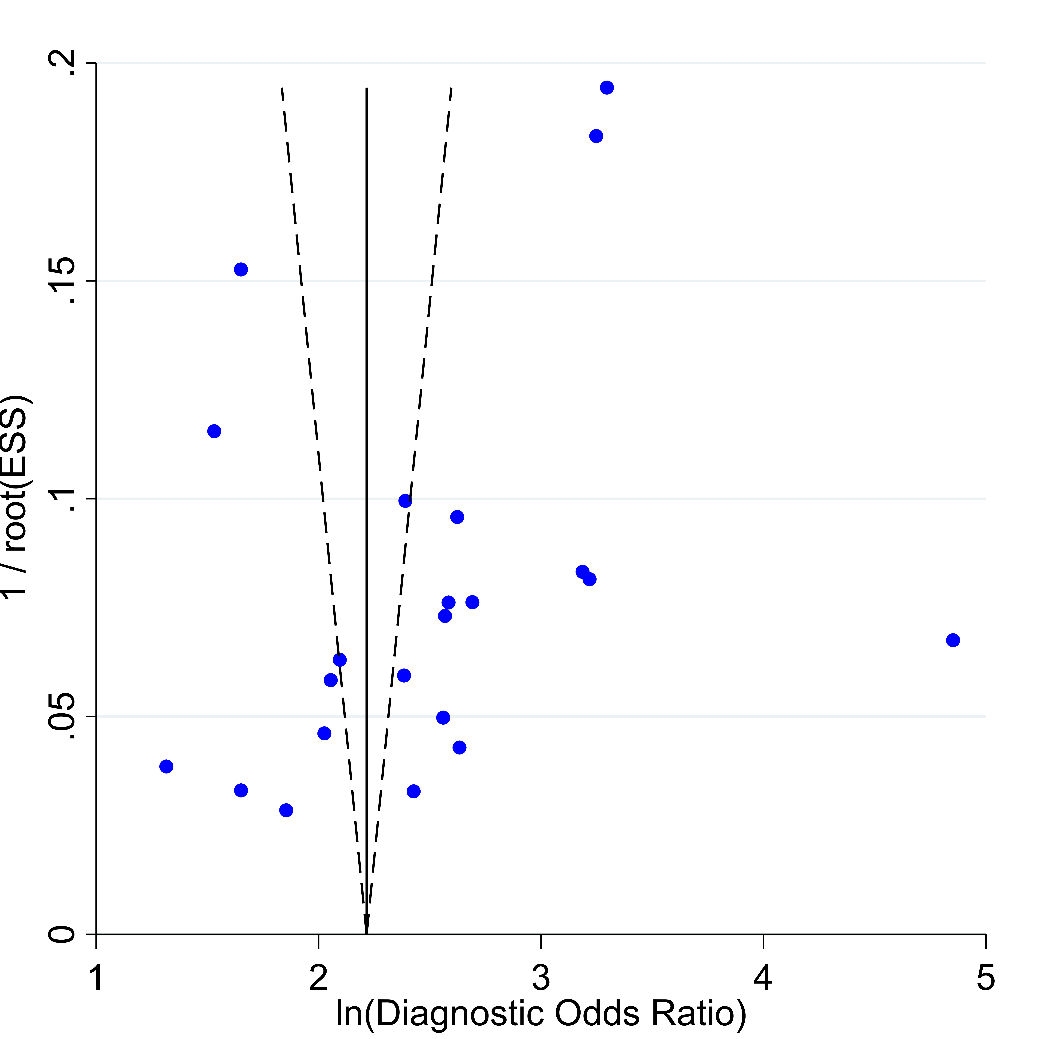


**Supplemental figure 25.** Deeks’ funnel plot for colposcopy (low-grade) to detect CIN2+. Abbreviations: ESS = effective sample size.


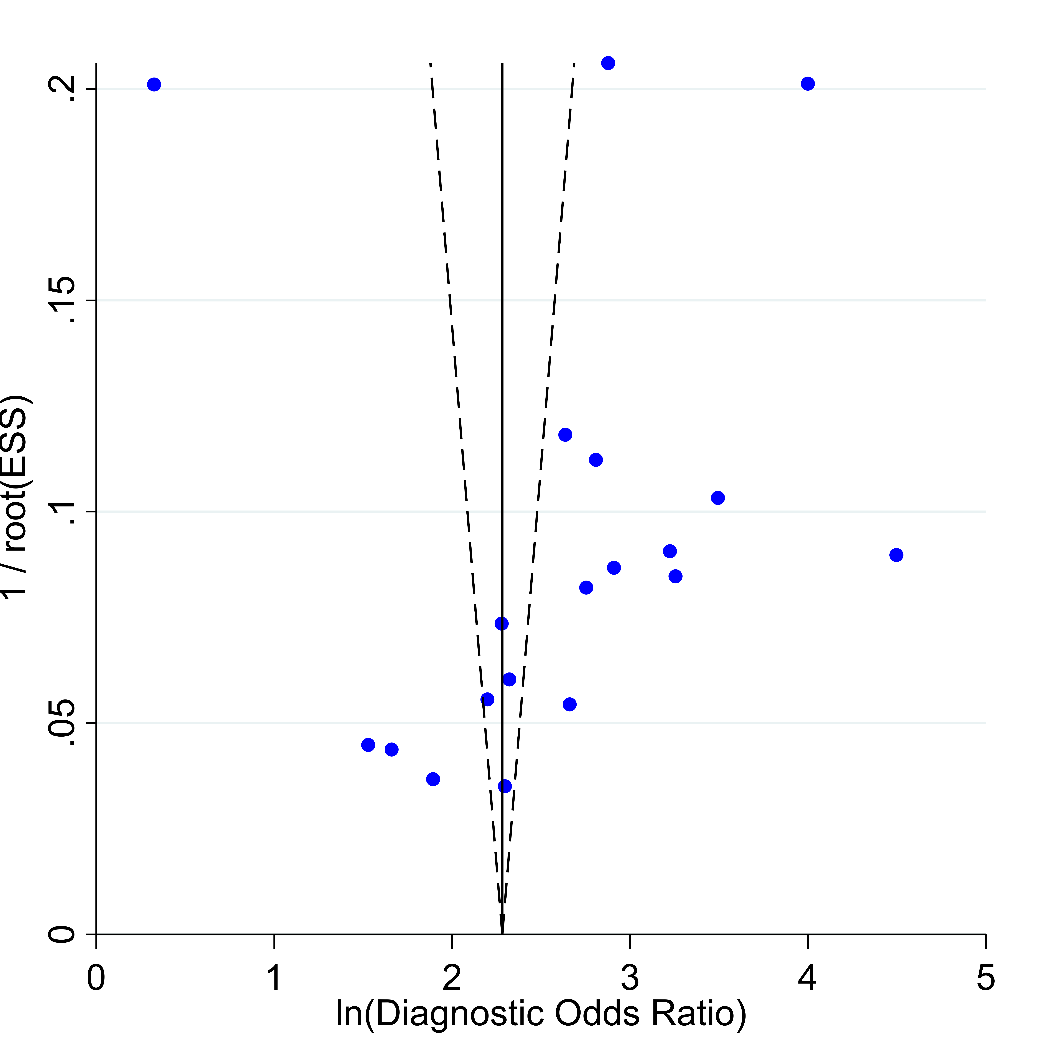


**Supplemental figure 26.** Deeks’ funnel plot for colposcopy (low-grade) to detect CIN3+. Abbreviations: ESS = effective sample size.


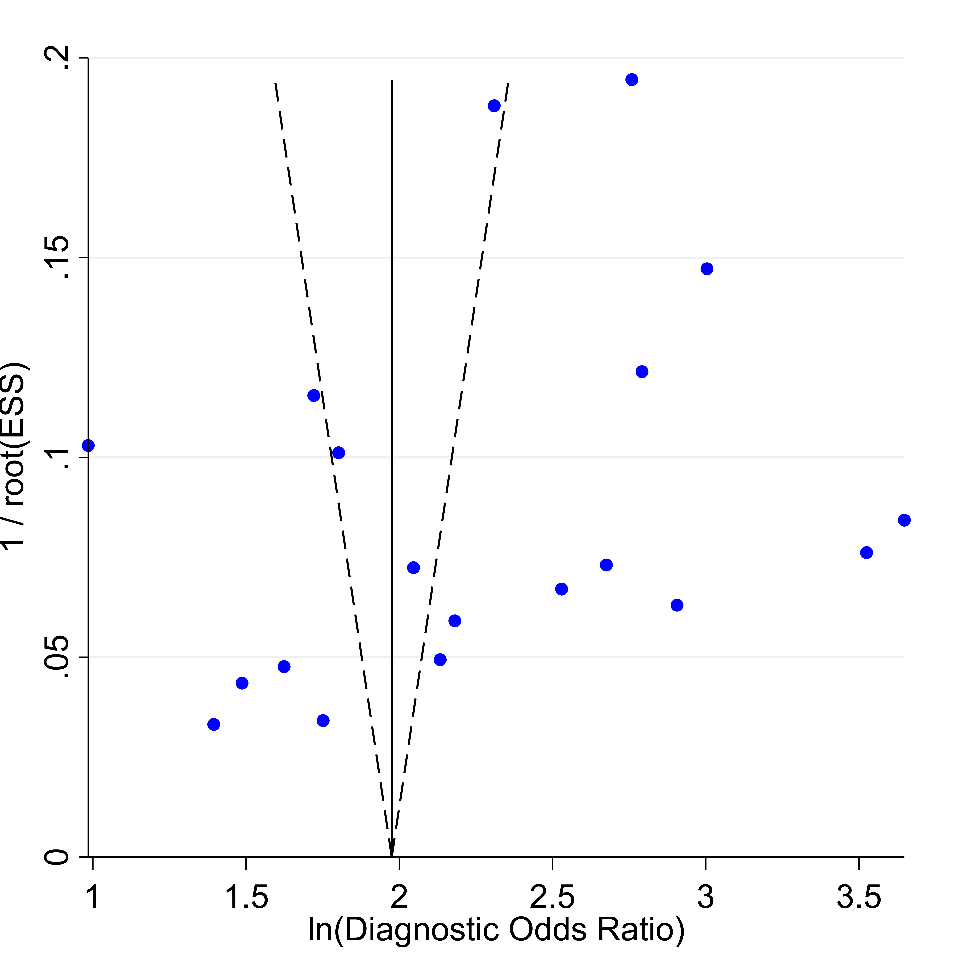


**Supplemental figure 27.** Deeks’ funnel plot for cytology (ASC-US) to detect CIN2+. Abbreviations: ESS = effective sample size.


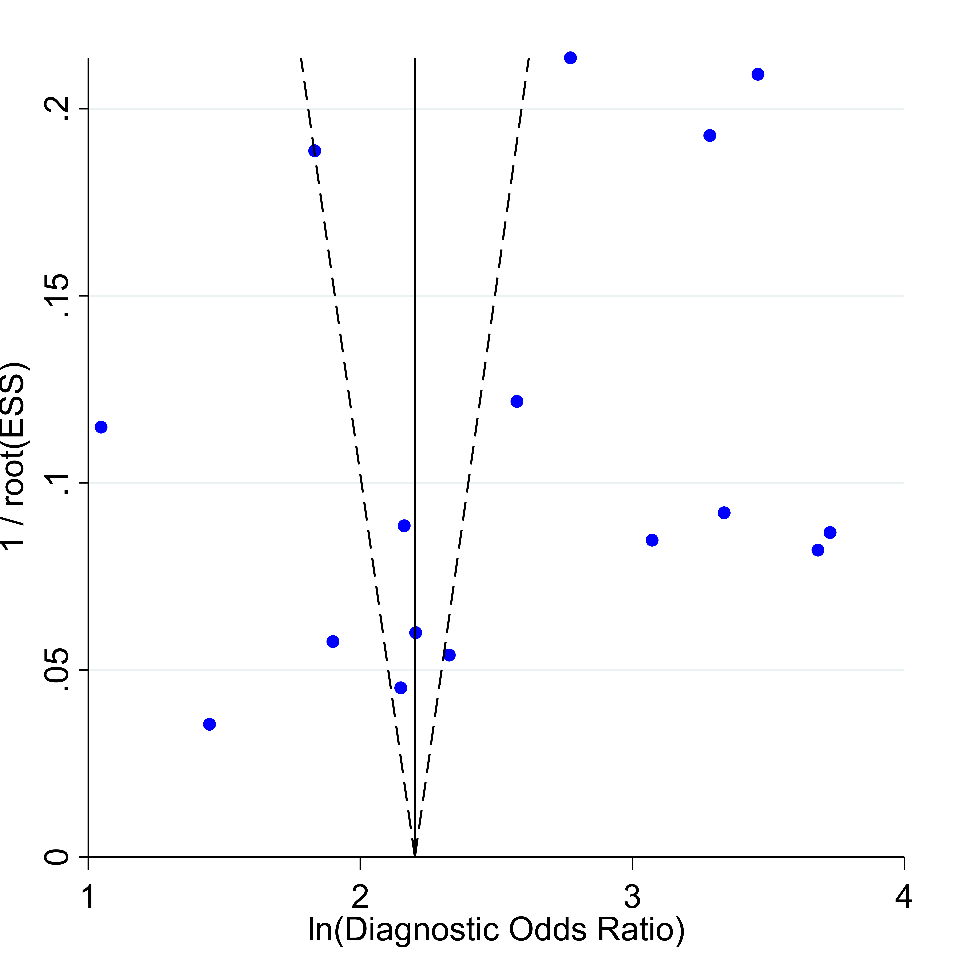


**Supplemental figure 28.** Deeks’ funnel plot for cytology (ASC-US) to detect CIN3+. Abbreviations: ESS = effective sample size.

1. Positive and negative predictive values

The pooled estimates for sensitivity (Se) and specificity (Sp) based on studies with complete follow-up (N) were used to calculate predictive values at different prevalence levels of CIN2+ (supplemental table 11) and CIN3+ (supplemental table 12).

The following parameters were calculated:

- The number of true positives (TP), false negatives (FN), false positives (FP) and true negatives (TN) in a population of 1000 women who tested HPV-positive at cervical cancer screening;
  - The number of TP corresponds to the number of CIN2+ / CIN3+ detected by the triage strategy,
  - The number of FP corresponds to the number of CIN2+ / CIN3+ missed by the triage strategy;
- Positive predictive value (PPV): number of CIN2+ / CIN3+ cases among 100 triage positive women;
- 1 / PPV: number of women who have to be treated to find one CIN2+ / CIN3+ case;
- Negative predictive value (NPV): number of disease free women among 100 triage negative women;
- Complement of NPV (cNPV): number of CIN2+ / CIN3+ cases among 100 triage negative women;
- Test positivity rate (TPR): number of positive triage tests among 100 women who tested HPV-positive at cervical cancer screening.

**supplemental Table 11.** Predictive values for CIN2+ in the triage of HPV-positive women using colposcopy with low-grade colposcopic impression as test cut-off. Predictive values were calculated using the pooled estimates for sensitivity (Se) and specificity (Sp) based on studies with complete follow-up and using different prevalence levels of CIN2+. Abbreviations are explained on page 32.

| **CIN2+ prevalence** | **Triage test (cut-off)** | **N** | **Se** | **Sp** | **TP** | **FN** | **FP** | **TN** | **PPV** | **1/PPV** | **NPV** | **cNPV** | **TPR** |
| --- | --- | --- | --- | --- | --- | --- | --- | --- | --- | --- | --- | --- | --- |
| 0·11 | Colposcopy (low-grade) | 16 | 0·84 | 0·64 | 93 | 17 | 318 | 572 | 22·6 | 4·4 | 97·1 | 2·9 | 41·1 |
|  | Colposcopy (high-grade) | 11 | 0·36 | 0·95 | 39 | 71 | 42 | 848 | 48·1 | 2·1 | 92·3 | 7·7 | 8·1 |
|  | Cytology (ASC-US) | 14 | 0·58 | 0·85 | 64 | 46 | 132 | 758 | 32·7 | 3·1 | 94·3 | 5·7 | 19·6 |
|  | Cytology (LSIL) | 11 | 0·46 | 0·91 | 51 | 59 | 81 | 809 | 38·6 | 2·6 | 93·2 | 6·8 | 13·2 |
|  | Cytology (HSIL) | 12 | 0·29 | 0·98 | 32 | 78 | 21 | 869 | 60·4 | 1·7 | 91·8 | 8·2 | 5·3 |
| 0·14 | Colposcopy (low-grade) | 16 | 0·84 | 0·64 | 118 | 22 | 307 | 553 | 27·8 | 3·6 | 96·2 | 3·8 | 42·5 |
|  | Colposcopy (high-grade) | 11 | 0·36 | 0·95 | 50 | 90 | 40 | 820 | 55·6 | 1·8 | 90·1 | 9·9 | 9·0 |
|  | Cytology (ASC-US) | 14 | 0·58 | 0·85 | 81 | 59 | 127 | 733 | 38·9 | 2·6 | 92·6 | 7·4 | 20·8 |
|  | Cytology (LSIL) | 11 | 0·46 | 0·91 | 64 | 76 | 78 | 782 | 45·1 | 2·2 | 91·1 | 8·9 | 14·2 |
|  | Cytology (HSIL) | 12 | 0·29 | 0·98 | 41 | 99 | 21 | 839 | 66·1 | 1·5 | 89·4 | 10·6 | 6·2 |
| 0·20 | Colposcopy (low-grade) | 16 | 0·84 | 0·64 | 169 | 31 | 286 | 514 | 37·1 | 2·7 | 94·3 | 5·7 | 45·5 |
|  | Colposcopy (high-grade) | 11 | 0·36 | 0·95 | 71 | 129 | 38 | 762 | 65·1 | 1·5 | 85·5 | 14·5 | 10·9 |
|  | Cytology (ASC-US) | 14 | 0·58 | 0·85 | 116 | 84 | 118 | 682 | 49·6 | 2·0 | 89·0 | 11·0 | 23·4 |
|  | Cytology (LSIL) | 11 | 0·46 | 0·91 | 92 | 108 | 73 | 727 | 55·8 | 1·8 | 87·1 | 12·9 | 16·5 |
|  | Cytology (HSIL) | 12 | 0·29 | 0·98 | 59 | 141 | 19 | 781 | 75·6 | 1·3 | 84·7 | 15·3 | 7·8 |

**supplemental Table 12.** Predictive values for CIN3+ in the triage of HPV-positive women using colposcopy with low-grade colposcopic impression as test cut-off. Predictive values were calculated using the pooled estimates for sensitivity (Se) and specificity (Sp) based on studies with complete follow-up and using different prevalence levels of CIN3+. Abbreviations are explained on page 32.

| **CIN3+ prevalence** | **Triage test (cut-off)** | **N** | **Se** | **Sp** | **TP** | **FN** | **FP** | **TN** | **PPV** | **1/PPV** | **NPV** | **cNPV** | **TPR** |
| --- | --- | --- | --- | --- | --- | --- | --- | --- | --- | --- | --- | --- | --- |
| 0·05 | Colposcopy (low-grade) | 15 | 0·87 | 0·62 | 43 | 7 | 359 | 591 | 10·7 | 9·3 | 98·8 | 1·2 | 40·2 |
|  | Colposcopy (high-grade) | 10 | 0·45 | 0·95 | 22 | 28 | 48 | 903 | 31·4 | 3·2 | 97·0 | 3·0 | 7·0 |
|  | Cytology (ASC-US) | 13 | 0·64 | 0·84 | 32 | 18 | 155 | 795 | 17·1 | 5·8 | 97·8 | 2·2 | 18·7 |
|  | Cytology (LSIL) | 11 | 0·51 | 0·90 | 26 | 24 | 99 | 852 | 20·8 | 4·8 | 97·3 | 2·7 | 12·5 |
|  | Cytology (HSIL) | 11 | 0·38 | 0·97 | 19 | 31 | 26 | 924 | 42·2 | 2·4 | 96·8 | 3·2 | 4·5 |
| 0·09 | Colposcopy (low-grade) | 15 | 0·87 | 0·62 | 78 | 12 | 344 | 566 | 18·5 | 5·4 | 97·9 | 2·1 | 42·2 |
|  | Colposcopy (high-grade) | 10 | 0·45 | 0·95 | 40 | 50 | 46 | 865 | 46·5 | 2·2 | 94·5 | 5·5 | 8·6 |
|  | Cytology (ASC-US) | 13 | 0·64 | 0·84 | 58 | 32 | 148 | 762 | 28·2 | 3·6 | 96·0 | 4·0 | 20·6 |
|  | Cytology (LSIL) | 11 | 0·51 | 0·90 | 46 | 44 | 95 | 816 | 32·6 | 3·1 | 94·9 | 5·1 | 14·1 |
|  | Cytology (HSIL) | 11 | 0·38 | 0·97 | 34 | 56 | 25 | 885 | 57·6 | 1·7 | 94·0 | 6·0 | 5·9 |
| 0·17 | Colposcopy (low-grade) | 15 | 0·87 | 0·62 | 148 | 22 | 314 | 516 | 32·0 | 3·1 | 95·9 | 4·1 | 46·2 |
|  | Colposcopy (high-grade) | 10 | 0·45 | 0·95 | 76 | 94 | 42 | 789 | 64·4 | 1·6 | 89·4 | 10·6 | 11·8 |
|  | Cytology (ASC-US) | 13 | 0·64 | 0·84 | 109 | 61 | 135 | 695 | 44·7 | 2·2 | 91·9 | 8·1 | 24·4 |
|  | Cytology (LSIL) | 11 | 0·51 | 0·90 | 87 | 83 | 86 | 744 | 50·3 | 2·0 | 89·9 | 10·1 | 17·3 |
|  | Cytology (HSIL) | 11 | 0·38 | 0·97 | 65 | 105 | 22 | 808 | 74·7 | 1·3 | 88·5 | 11·5 | 8·7 |

1. Certainty of evidence

The Risk Of Bias In Non-randomised Studies of Interventions (ROBINS-I) tool was adjusted to create a framework for the assessment of certainty of evidence for diagnostic accuracy studies.^17^ Based on all included studies, the impact on certainty of evidence was expressed for each triage strategy and outcome as not serious (NS), serious (S) or very serious (VS) for different factors as explained below (supplemental table 14). The assessment was repeated for studies with complete follow-up (supplemental table 15).

- The number of studies.
- The number of patients.
- Risk of bias, based on the QUADAS assessment:
  - NS if < 10% of items scored ‘No’;
  - S if ≥ 10% to < 20% of items scored ‘No’;
  - VS if ≥ 20% of items scored ‘No’.
- Indirectness, based on whether outcome CIN2+ or CIN3+ was evaluated:
  - S if CIN3+ was evaluated;
  - VS if CIN2+ was evaluated;
- Heterogeneity, based on I^2^:
  - NS if I^2^ < 70.0 for sensitivity and specificity;
  - S if I^2^ ≥ 70.0 for sensitivity or specificity;
  - VS if I^2^ ≥ 70.0 for sensitivity and specificity.
- Inconsistency, by comparing results at different thresholds:
  - NS if sensitivity at lower thresholds > sensitivity at higher thresholds and if specificity at lower thresholds < specificity at higher thresholds.
- Imprecision, based on the width of the 95% confidence interval (CI) around the pooled estimate of the relative sensitivity / specificity:
  - NS if width < 0.2 for relative sensitivity and relative specificity;
  - S if width ≥ 0.2 for relative sensitivity or relative specificity;
  - VS if width ≥ 0.2 for relative sensitivity and relative specificity.
- Reproducibility, based on expert opinion:
  - (V)S: colposcopy (index test), cytology (comparator test) and histopathology (reference test) are all subjective tests that rely on interpretation of the test assessor.

The assessment results for each triage strategy and outcome were summarized per outcome (supplemental table 13). Factors which scored mostly NS are shown as Low risk, factors which scored mostly S are shown as Medium risk and factors which scored mostly VS are shown as (Very) High risk.

**supplemental Table 13.** Summary of the assessment on certainty of evidence.

|  | Factors that may decrease certainty of evidence | | | | | | | |
| --- | --- | --- | --- | --- | --- | --- | --- | --- |
|  | N  studies | N  patients | Risk of bias | Indirectness | Heterogeneity | Inconsistency | Imprecision | Reproducibility |
|  |  |  | QUADAS | Detection  CIN2+ or CIN3+ | I^2^ | Gradient  by cut-off | Width 95% CI | Expert opinion |
| **All included studies** |  | | | | | | | |
| CIN2+ | 11 | 13487 | Medium risk | High risk | High risk | Low risk | Medium risk | Very high risk |
| CIN3+ | 8 | 12026 | Medium risk | Medium risk | High risk | Low risk | Medium risk | High risk |
| **Studies with complete follow-up** |  | | | | | | | |
| CIN2+ | 8 | 10808 | Low risk | High risk | High risk | Low risk | Medium risk | Very high risk |
| CIN3+ | 7 | 10726 | Low risk | Medium risk | High risk | Low risk | Medium risk | High risk |

**supplemental Table 14.** Certainty of evidence based on all included studies.

|  | Factors that may decrease certainty of evidence | | | | | | | | | | | |
| --- | --- | --- | --- | --- | --- | --- | --- | --- | --- | --- | --- | --- |
|  | N  studies | N  patients | Risk of bias | Indirectness | Heterogeneity (I^2^) | | | Inconsistency | Imprecision (width 95% CI) | | | Repro-ducibility |
|  |  |  | QUADAS | Detection CIN2+/3+ | Sensitivity | Specificity | Judgment | Gradient by cut-off | Relative sensitivity | Relative specificity | Judgment | Expert opinion |
| **CIN2+** |  | | | | | | | | | | | |
| L-grade colpo | 11 | 13311 | 17/143 (11.9%) of QUADAS items scored as high risk of bias | VS | 56.7 | 94.1 | S | NS | - | - | - | VS |
| H-grade colpo | 4 | 7657 |  |  | 62.0 | 79.7 | S |  | - | - | - |  |
| ASC-US+ cyto | 9 | 8855 |  |  | 82.2 | 91.1 | VS |  | - | - | - |  |
| LSIL+ cyto | 6 | 8324 |  |  | 82.4 | 85.8 | VS |  | - | - | - |  |
| HSIL+ cyto | 7 | 8406 |  |  | 82.8 | 66.5 | S |  | - | - | - |  |
| L-grade vs ASC-US+ | 9 | 9746 |  |  | - | - | - |  | 0.28 | 0.08 | S |  |
| L-grade vs LSIL+ | 6 | 8548 |  |  | - | - | - |  | 0.53 | 0.09 | S |  |
| L-grade vs HSIL+ | 7 | 8630 |  |  | - | - | - |  | 1.28 | 0.12 | S |  |
| H-grade vs ASC-US+ | 3 | 6393 |  |  | - | - | - |  | 0.19 | 0.07 | NS |  |
| H-grade vs LSIL+ | 2 | 6311 |  |  | - | - | - |  | 0.27 | 0.02 | S |  |
| H-grade vs HSIL+ | 3 | 6393 |  |  | - | - | - |  | 0.62 | 0.03 | S |  |
| **CIN3+** |  | | | | | | | | | | | |
| L-grade colpo | 8 | 11850 | 17/143 (11.9%) of QUADAS items scored as high risk of bias | S | 43.2 | 95.1 | S | NS | - | - | - | S |
| H-grade colpo | 2 | 7113 |  |  | 15.7 | 87.1 | S |  | - | - | - |  |
| ASC-US+ cyto | 6 | 7418 |  |  | 76.6 | 90.0 | VS |  | - | - | - |  |
| LSIL+ cyto | 4 | 6969 |  |  | 77.3 | 83.7 | VS |  | - | - | - |  |
| HSIL+ cyto | 4 | 6969 |  |  | 80.8 | 75.9 | VS |  | - | - | - |  |
| L-grade vs ASC-US+ | 6 | 8285 |  |  | - | - | - |  | 0.38 | 0.10 | S |  |
| L-grade vs LSIL+ | 4 | 7169 |  |  | - | - | - |  | 0.68 | 0.13 | S |  |
| L-grade vs HSIL+ | 4 | 7169 |  |  | - | - | - |  | 1.20 | 0.14 | S |  |
| H-grade vs ASC-US+ | 1 | 5849 |  |  | - | - | - |  | 0.22 | 0.04 | S |  |
| H-grade vs LSIL+ | 1 | 5849 |  |  | - | - | - |  | 0.30 | 0.02 | S |  |
| H-grade vs HSIL+ | 1 | 5849 |  |  | - | - | - |  | 0.59 | 0.03 | S |  |

**supplemental Table 15.** Certainty of evidence based on studies with complete follow-up.

|  | Factors that may decrease certainty of evidence | | | | | | | | | | | |
| --- | --- | --- | --- | --- | --- | --- | --- | --- | --- | --- | --- | --- |
|  | N  studies | N  patients | Risk of bias | Indirectness | Heterogeneity (I^2^) | | | Inconsistency | Imprecision (width 95% CI) | | | Repro-ducibility |
|  |  |  | QUADAS | Detection CIN2+/3+ | Sensitivity | Specificity | Judgment | Gradient by cut-off | Relative sensitivity | Relative specificity | Judgment | Expert opinion |
| **CIN2+** |  | | | | | | | | | | | |
| L-grade colpo | 8 | 10632 | 6/104 (5.8%) of QUADAS items scored as high risk of bias | VS | 57.9 | 92.2 | S | NS | - | - | - | VS |
| H-grade colpo | 3 | 7195 |  |  | 54.5 | 80.3 | S |  | - | - | - |  |
| ASC-US+ cyto | 6 | 6655 |  |  | 81.5 | 88 | VS |  | - | - | - |  |
| LSIL+ cyto | 3 | 6124 |  |  | 79.4 | 82.3 | VS |  | - | - | - |  |
| HSIL+ cyto | 4 | 6206 |  |  | 76.2 | 61.4 | S |  | - | - | - |  |
| L-grade vs ASC-US+ | 6 | 7470 |  |  | - | - | - |  | 0.44 | 0.11 | S |  |
| L-grade vs LSIL+ | 3 | 6272 |  |  | - | - | - |  | 0.85 | 0.13 | S |  |
| L-grade vs HSIL+ | 4 | 6354 |  |  | - | - | - |  | 1.71 | 0.15 | S |  |
| H-grade vs ASC-US+ | 2 | 5931 |  |  | - | - | - |  | 0.20 | 0.06 | S |  |
| H-grade vs LSIL+ | 1 | 5849 |  |  | - | - | - |  | 0.30 | 0.02 | S |  |
| H-grade vs HSIL+ | 2 | 5931 |  |  | - | - | - |  | 0.65 | 0.03 | S |  |
| **CIN3+** |  | | | | | | | | | | | |
| L-grade colpo | 7 | 10550 | 6/104 (5.8%) of QUADAS items scored as high risk of bias | S | 43.5 | 93.7 | S | NS | - | - | - | S |
| H-grade colpo | 2 | 7113 |  |  | 15.7 | 87.1 | S |  | - | - | - |  |
| ASC-US+ cyto | 5 | 6573 |  |  | 76.1 | 91.1 | VS |  | - | - | - |  |
| LSIL+ cyto | 3 | 6124 |  |  | 74.2 | 85.4 | VS |  | - | - | - |  |
| HSIL+ cyto | 3 | 6124 |  |  | 76.3 | 72.5 | VS |  | - | - | - |  |
| L-grade vs ASC-US+ | 5 | 7388 |  |  | - | - | - |  | 0.50 | 0.11 | S |  |
| L-grade vs LSIL+ | 3 | 6272 |  |  | - | - | - |  | 0.86 | 0.13 | S |  |
| L-grade vs HSIL+ | 3 | 6272 |  |  | - | - | - |  | 1.66 | 0.15 | S |  |
| H-grade vs ASC-US+ | 1 | 5849 |  |  | - | - | - |  | 0.22 | 0.04 | S |  |
| H-grade vs LSIL+ | 1 | 5849 |  |  | - | - | - |  | 0.30 | 0.02 | S |  |
| H-grade vs HSIL+ | 1 | 5849 |  |  | - | - | - |  | 0.59 | 0.03 | S |  |

1. References

1. Visual inspection with acetic acid for cervical-cancer screening: test qualities in a primary-care setting. University of Zimbabwe/JHPIEGO Cervical Cancer Project. *Lancet*. Mar 13 1999;353(9156):869-73.

2. Womack SD, Chirenje ZM, Blumenthal PD, et al. Evaluation of a human papillomavirus assay in cervical screening in Zimbabwe. Article. *British Journal of Obstetrics and Gynaecology*. 2000;107(1):33-38.

3. Blumenthal PD, Gaffikin L, Chirenje ZM, McGrath J, Womack S, Shah K. Adjunctive testing for cervical cancer in low resource settings with visual inspection, HPV, and the Pap smear. *Int J Gynaecol Obstet*. Jan 2001;72(1):47-53. doi:10.1016/s0020-7292(00)00329-5

4. Belinson J, Qiao YL, Pretorius R, et al. Shanxi Province Cervical Cancer Screening Study: a cross-sectional comparative trial of multiple techniques to detect cervical neoplasia. *Gynecol Oncol*. Nov 2001;83(2):439-44. doi:10.1006/gyno.2001.6370

5. Belinson JL, Qiao YL, Pretorius RG, et al. Shanxi Province cervical cancer screening study II: self-sampling for high-risk human papillomavirus compared to direct sampling for human papillomavirus and liquid based cervical cytology. *Int J Gynecol Cancer*. Nov-Dec 2003;13(6):819-26. doi:10.1111/j.1525-1438.2003.13611.x

6. Sankaranarayanan R, Chatterji R, Shastri SS, et al. Accuracy of human papillomavirus testing in primary screening of cervical neoplasia: results from a multicenter study in India. *Int J Cancer*. Nov 1 2004;112(2):341-7. doi:10.1002/ijc.20396

7. Hovland S, Arbyn M, Lie AK, et al. A comprehensive evaluation of the accuracy of cervical pre-cancer detection methods in a high-risk area in East Congo. Article. *British Journal of Cancer*. 2010;102(6):957-965. doi:10.1038/sj.bjc.6605594

8. Poli UR, Gowrishankar S, Swain M, Jeronimo J. Triage of Women Testing Positive With the careHPV Test on Self-Collected Vaginal Samples for Cervical Cancer Screening in a Low-Resource Setting. *J Glob Oncol*. Sep 2018;4:1-7. doi:10.1200/jgo.2016.008078

9. Luckett R, Mogowa N, Li HJ, et al. Performance of Two-Stage Cervical Cancer Screening With Primary High-Risk Human Papillomavirus Testing in Women Living With Human Immunodeficiency Virus. *Obstet Gynecol*. Oct 2019;134(4):840-849. doi:10.1097/aog.0000000000003496

10. Almonte M, Murillo R, Sánchez GI, et al. Multicentric study of cervical cancer screening with human papillomavirus testing and assessment of triage methods in Latin America: the ESTAMPA screening study protocol. *BMJ Open*. May 24 2020;10(5):e035796. doi:10.1136/bmjopen-2019-035796

11. Valls J, Baena A, Venegas G, et al. Performance of standardised colposcopy to detect cervical precancer and cancer for triage of women testing positive for human papillomavirus: results from the ESTAMPA multicentric screening study. *Lancet Glob Health*. Mar 2023;11(3):e350-e360. doi:10.1016/s2214-109x(22)00545-9

12. Dang L, Kong L, Zhao Y, et al. Evaluation of triage strategies for high-risk human papillomavirus-positive women in cervical cancer screening: A multicenter randomized controlled trial in different resource settings in China. *Chin J Cancer Res*. Oct 30 2022;34(5):496-509. doi:10.21147/j.issn.1000-9604.2022.05.09

13. Zhang J, Zhao Y, Dai Y, et al. Effectiveness of High-risk Human Papillomavirus Testing for Cervical Cancer Screening in China: A Multicenter, Open-Label, Randomized Clinical Trial. *JAMA Oncol*. Feb 1 2021;7(2):263-270. doi:10.1001/jamaoncol.2020.6575

14. Luckett R, Ramogola-Masire D, Gompers A, et al. Triage of HPV positivity in a high HIV prevalence setting: A prospective cohort study comparing visual triage methods and HPV genotype restriction in Botswana. *Int J Gynaecol Obstet*. Nov 10 2023;doi:10.1002/ijgo.15225

15. Tan JHJ, Jones A, Steele A, Vicario E, Wrede CDH. Diagnostic outcomes after colposcopy at a tertiary clinic for women aged 50-74 referred after detection of oncogenic human papillomavirus at primary screening. *Aust N Z J Obstet Gynaecol*. Aug 2023;63(4):564-570. doi:10.1111/ajo.13684

16. Deeks JJ, Macaskill P, Irwig L. The performance of tests of publication bias and other sample size effects in systematic reviews of diagnostic test accuracy was assessed. *J Clin Epidemiol* 2005;58(9):882-893.

17. Sterne JA, Hernán MA, Reeves BC, et al. ROBINS-I: a tool for assessing risk of bias in non-randomised studies of interventions. *BMJ* Oct 12 2016;355:i4919. doi: 10.1136/bmj.i4919.
