# Supplementary material for: Accuracy of colposcopy to triage HPV-positive women in cervical cancer screening: a systematic review and meta-analysis
Source: eClinicalMedicine. 2026 Jun 17;96:104009. doi: 10.1016/j.eclinm.2026.104009 (PMC13311182; doi:10.1016/j.eclinm.2026.104009)
Supplement: Translated Abstracts [file mmc2.pdf]

The following translations in Spanish, Chinese, and Hindi were submitted by the authors and we reproduce them as supplied. They have not been peer reviewed. Our editorial processes have only been applied to the original abstract in English, which should serve as reference for this manuscript.

Translation of the Summary in Spanish

Resumen

Antecedentes: Las guías de la Organización Mundial de la Salud (OMS) para el tamizaje del cáncer de cuello uterino recomiendan la colposcopia como uno de los métodos de triaje en mujeres con resultado positivo para el virus del papiloma humano (VPH). El objetivo fue evaluar la precisión diagnóstica de la colposcopia en este contexto.

Métodos: Se realizó una revisión sistemática y metaanálisis de estudios que reportaron la precisión diagnóstica de la colposcopia para detectar neoplasia intraepitelial cervical grado 2 o peor (NIC2+) y grado 3 o peor (NIC3+) en mujeres con prueba de ADN del VPH positiva, publicados en PubMed, Embase y Cochrane hasta el 20 de octubre de 2025. Se incluyeron estudios observacionales y aleatorizados con diseño transversal (es decir, el triaje mediante colposcopia y la biopsia se realizaron al mismo tiempo) o diseño longitudinal (es decir, el triaje mediante colposcopia y el estándar de referencia no se realizaron al mismo tiempo). Se excluyeron los estudios si la población no era representativa de una población de tamizaje o si no se podían recuperar datos agregados sobre el triaje con colposcopia a partir del informe publicado o tras contactar con los autores. Se recopilaron datos del triaje con colposcopia (prueba índice) junto con datos del triaje mediante citología (comparador), cuando estaban disponibles, y se evaluó el riesgo de sesgo utilizando una versión adaptada de la lista de verificación QUADAS-2 (Quality Assessment of Diagnostic Accuracy Studies). Se calcularon la sensibilidad y especificidad agrupadas para detectar NIC2+ y NIC3+, así como la sensibilidad y especificidad relativas del triaje con colposcopia en comparación con el triaje con citología, mediante un modelo bivariado de efectos aleatorios logísticos. La heterogeneidad se midió mediante τ^2 e I^2. Los efectos del tamaño muestral se evaluaron mediante gráficos en embudo de Deeks y la prueba de regresión. La certeza de la evidencia se evaluó utilizando una versión adaptada de la herramienta ROBINS-I (Risk Of Bias In Non-randomised Studies of Interventions). El estudio fue registrado en PROSPERO (CRD42023389772).

Hallazgos: El estudio identificó 5774 registros. Tras el proceso de selección, se incluyeron 11 estudios (13311 participantes), pero el análisis principal se restringió a 8 estudios (10632 participantes) con seguimiento completo tras el triaje con colposcopia. La sensibilidad combinada para NIC2+ y NIC3+ utilizando colposcopia (impresión colposcópica de bajo grado) fue del 84,4% (IC del 95%: 79,0%–88,6%) y del 86,9% (IC del 95%: 81,7%–90,8%), respectivamente; la especificidad agrupada para NIC2+ fue del 64,3% (IC del 95%: 57,4%–70,7%). Aunque la heterogeneidad entre estudios fue alta (I^2 hasta 95,5%), la mayoría de los estudios reportaron una sensibilidad mayor y una especificidad menor en comparación con la citología (células escamosas atípicas de significado indeterminado [ASC-US]): la sensibilidad relativa agrupada para NIC2+ y NIC3+ fue de 1,74 (IC del 95%: 1,53–1,97) y 1,58 (IC del 95%: 1,35–1,85), respectivamente. La especificidad relativa agrupada para NIC2+ fue de 0,67 (IC del 95%: 0,61–0,72). La certeza de la evidencia se consideró baja a muy baja debido a la subjetividad en la evaluación de la colposcopia, la citología y el estándar de referencia, así como a la alta heterogeneidad entre estudios y el riesgo de clasificación errónea del estándar de referencia.

Interpretación: Los estudios reportaron de manera consistente una buena sensibilidad, pero una baja especificidad de la colposcopia para el triaje de mujeres con una prueba de VPH positiva, lo que indica que la colposcopia puede ser una prueba de triaje aceptable en contextos donde existe una pérdida significativa de seguimiento tras el tamizaje, aunque con riesgo de sobretratamiento. Sin embargo,

debido a la subjetividad intrínseca de las evaluaciones y a la elevada heterogeneidad entre estudios, el nivel de evidencia se consideró bajo a muy bajo. Por lo tanto, no es posible recomendar una única prueba de triaje óptima, y las recomendaciones deben tener en cuenta el contexto específico, así como la experiencia, los recursos y la infraestructura disponibles. Los resultados de esta revisión respaldan las directrices de la OMS sobre el tamizaje del cáncer de cuello uterino.

Financiación: Organización Mundial de la Salud (acuerdo para realización de guías sobre detección y tratamiento de la enfermedad cervical preinvasiva); Horizon 2020 Framework Programme for Research and Innovation of the European Commission, a través de la Red RISCC (Subvención No. 847845), Joint Action EUCanScreen (Proyecto No. 101162959), y la European Commission Initiative on Cervical Cancer (EC-CvC).

Translation of the Summary in Chinese

摘要

背景：世界卫生组织（WHO）宫颈癌筛查指南建议，将阴道镜检查作为对人乳头瘤病毒（HPV）阳性女性进行分流（triage）的方法之一。本研究旨在评估在该情境下阴道镜检查的诊断准确性。

方法： 在本系统综述与荟萃分析中，我们检索了截至2025年10月20日发表在 PubMed、Embase 或 Cochrane Library 中、报告阴道镜检查用于检出宫颈上皮内瘤变2级及以上（CIN2+）和3级及以上（CIN3+）且研究对象为 HPV DNA 筛查阳性女性的诊断准确性研究。纳入研究类型包括观察性研究与随机研究，研究设计可为横断面设计（即阴道镜分流与活检同时进行）或纵向设计（即阴道镜分流与参考标准并非同时进行）。若研究人群不能代表筛查人群，或无法从发表报告中提取阴道镜分流的汇总数据、且在联系作者后仍无法获得相关数据，则予以排除。我们收集阴道镜分流（待评估指标）数据，并在可获得时同步收集细胞学分流（对照方法）数据；采用经调整的诊断试验质量评价量表（QUADAS-2）评估偏倚风险。使用双变量逻辑随机效应模型合并计算检出 CIN2+ 与 CIN3+ 的灵敏度与特异度，并计算阴道镜分流相较于细胞学分流的相对灵敏度与相对特异度。异质性通过 τ^2 与 I^2 衡量；样本量效应通过 Deeks 漏斗图及回归检验评估。证据确定性采用经调整的非随机干预研究偏倚风险评估工具（ROBINS-I）评价。本综述已在 PROSPERO 注册（CRD42023389772）。

结果： 文献检索共获得5774条记录。筛选后纳入11项研究（13311名参与者），但主要分析限定为8项在阴道镜分流后具有完整随访的研究（10632名参与者）。以阴道镜（阴道镜表象为低级别）进行分流时，合并灵敏度在检出 CIN2+ 和 CIN3+ 方面分别为84•4%（95% CI：79•0%–88•6%）与86•9%（95% CI：81•7–90•8%）；检出 CIN2+ 的合并特异度为64•3%（95% CI：57•4%–70•7%）。尽管研究间异质性较高（I^2最高达95•5%），多数研究显示，与细胞学（意义不明确的非典型鳞状细胞，ASC-US）分流相比，阴道镜分流具有更高的灵敏度但更低的特异度：检出 CIN2+ 与 CIN3+ 的合并相对灵敏度分别为1•74（95% CI：1•53–1•97）与1•58（95% CI：1•35–1•85）；检出 CIN2+ 的合并相对特异度为0•67（95% CI：0•61–0•72）。由于阴道镜、细胞学及参考标准判读具有主观性，研究间异质性高，并存在参考标准误分型风险，证据确定性被评定为低至极低。

解释： 研究一致报告阴道镜用于 HPV 筛查阳性女性分流时具有良好灵敏度但特异度较低。这提示在筛查后失访率较高的环境中，阴道镜可作为一种可接受的分流检测，但可能增加过度治疗风险。然而，鉴于评估过程的内在主观性及研究间高度异质性，整体证据水平为低至极低。因此，无法推荐单一的最优分流检测；制定建议时应始终结合具体应用场景及可获得的专业能力与基础设施。总体而言，本综述结果支持 WHO 宫颈癌筛查指南。

资助： 世界卫生组织（关于《筛查与治疗宫颈癌前病变》指南的工作绩效协议）；欧盟委员会“Horizon 2020”研究与创新框架计划，通过 RISCC 网络（资助编号：847845）、联合行动 EUCanScreen（项目编号：101162959）以及欧盟委员会宫颈癌倡议（EC-CvC）。

Translation of the Summary in Hindi

सारांश

पृष्ठभूमि: विश्व स्वास्थ्य संगठन (WHO) के गर्भाशय ग्रीवा कैंसर स्क्रीनिंग संबंधी दिशानिर्देश मानव पैपिलोमावायरस (HPV)-सकारात्मक महिलाओं के ट्राइएज के लिए कोल्पोस्कोपी को एक विधि के रूप में अनुशंसित करते हैं। हमारा उद्देश्य इस संदर्भ में कोल्पोस्कोपी की निदान सटीकता का आकलन करना था।

विधि: इस व्यवस्थित समीक्षा और मेटा-विश्लेषण में, हमने उन लेखों की खोज की जो HPV डीएनए स्क्रीनिंग परीक्षण में सकारात्मक महिलाओं में सर्वाइकल इंट्राएपिथेलियल नियोप्लासिया ग्रेड 2 या उससे अधिक (CIN2+) और ग्रेड 3 या उससे अधिक (CIN3+) का पता लगाने के लिए कोल्पोस्कोपी की निदान सटीकता की रिपोर्ट करते हैं, जो 20 अक्टूबर 2025 तक PubMed, Embase या Cochrane लाइब्रेरी में प्रकाशित हुए हैं। अवलोकनात्मक और यादृच्छिक दोनों प्रकार के अध्ययन, चाहे उनका डिज़ाइन क्रॉस-सेक्शनल (यानी कोल्पोस्कोपी ट्राइएज और बायोप्सी एक ही समय में की गईं) या लंबवत (यानी कोल्पोस्कोपी ट्राइएज और संदर्भ मानक एक ही समय में नहीं किए गए) हो, शामिल किए जाने के लिए पात्र थे। अध्ययन को तब बाहर रखा गया यदि अध्ययन आबादी स्क्रीनिंग आबादी का प्रतिनिधि नहीं थी या यदि कोल्पोस्कोपी ट्राइएज पर संकलित डेटा प्रकाशित रिपोर्ट से या लेखकों से संपर्क करने के बाद प्राप्त नहीं किया जा सका। हमने कोल्पोस्कोपी ट्राइएज (सूचकांक) पर डेटा एकत्र किया, साथ ही साइटोलॉजी ट्राइएज (तुलनाकर्ता) पर डेटा, यदि उपलब्ध हो, और एक अनुकूलित गुणवत्ता मूल्यांकन ऑफ डायग्नोस्टिक एक्यूरेसी स्टडीज़ (QUADAS)-2 चेकलिस्ट का उपयोग करके पक्षपात के जोखिम का आकलन किया। द्विचर लॉजिस्टिक यादृच्छिक प्रभाव मॉडल का उपयोग करके CIN2+ और CIN3+ का पता लगाने के लिए समेकित संवेदनशीलता और विशिष्टता, साथ ही साइटोलॉजी ट्रांज़ के मुकाबले कोल्पोस्कोपी ट्रांज़ की सापेक्ष संवेदनशीलता और विशिष्टता की गणना की गई। विषमता को τ^2और $I^{(2)}$ का उपयोग करके मापा गया। नमूना आकार के प्रभावों का मूल्यांकन डीक्स के फनेल प्लॉट और प्रतिगमन परीक्षण द्वारा किया गया। सबूत की निश्चितता का आकलन एक अनुकूलित इंटरवेंशन के गैर-यादृच्छिक अध्ययनों में पक्षपात के जोखिम (ROBINS-I) उपकरण का उपयोग करके किया गया। इस समीक्षा को PROSPERO, CRD42023389772 के साथ पंजीकृत किया गया था।

निष्कर्ष: साहित्य खोज में 5774 रिकॉर्ड मिले। स्क्रीनिंग के बाद, हमने समीक्षा में 11 अध्ययन (13311 प्रतिभागी) शामिल किए, लेकिन प्राथमिक विश्लेषण को 8 अध्ययनों (10632 प्रतिभागी) तक सीमित कर दिया, जिनमें कोल्पोस्कोपी ट्राइएज के बाद पूर्ण फॉलो-अप था। कोल्पोस्कोपी (निम्न-श्रेणी का कोल्पोस्कोपिक इंप्रेशन) का उपयोग करके CIN2+ और CIN3+ के लिए पूल की गई संवेदनशीलता क्रमशः 84·4% (95% CI: 79·0% – 88·6%) और 86·9% (95% CI: 81·7 – 90·8%) थी; CIN2+ के लिए पूल की गई विशिष्टता 64.3% (95% CI: 57.4% – 70.7%) थी। हालांकि अध्ययनों के बीच विषमता अधिक थी, I^2 95.5% तक था, अधिकांश अध्ययनों ने साइटोलॉजी (अज्ञात महत्व की असामान्य स्क्वामस कोशिकाएं (ASC-US)) की तुलना में उच्च संवेदनशीलता और कम विशिष्टता की सूचना दी: CIN2+ और CIN3+ के लिए पूल की गई सापेक्ष संवेदनशीलता क्रमशः 1.74 (95% CI: 1.53 – 1.97) और 1.58 (95% CI: 1.35 – 1.85) थी। CIN2+ के लिए समेकित सापेक्ष विशिष्टता 0·67 (95% CI 0·61 – 0·72) थी। कोल्पोस्कोपी, साइटोलॉजी और संदर्भ मानक के मूल्यांकन में विषयगतता, साथ ही अध्ययनों के बीच उच्च विषमता और संदर्भ मानक के गलत वर्गीकरण के जोखिम के कारण साक्ष्य की निश्चितता को कम से बहुत कम माना गया।

व्याख्या: अध्ययनों ने लगातार अच्छी संवेदनशीलता लेकिन सकारात्मक एचपीवी स्क्रीनिंग परीक्षण वाली महिलाओं के ट्राइएज के लिए कोल्पोस्कोपी की कम विशिष्टता की सूचना दी, जो यह दर्शाता है कि कोल्पोस्कोपी उन परिस्थितियों में एक स्वीकार्य ट्राइएज परीक्षण हो सकता है जहाँ स्क्रीनिंग के बाद फॉलो-अप में महत्वपूर्ण कमी होती है, लेकिन ओवरट्रीटमेंट का खतरा होता है। हालाँकि, मूल्यांकन की अंतर्निहित व्यक्तिपरकता और अध्ययनों के बीच उच्च विषमता के कारण, साक्ष्य का स्तर कम से बहुत कम माना गया। इसलिए, एक एकल इष्टतम ट्राइएज परीक्षण की सिफारिश करना संभव नहीं है, और सिफारिशों में हमेशा उनकी विशिष्ट सेटिंग और उपलब्ध विशेषज्ञता और बुनियादी ढांचे को ध्यान में रखना चाहिए। कुल मिलाकर, इस समीक्षा के परिणाम गर्भाशय ग्रीवा के कैंसर की स्क्रीनिंग पर डब्ल्यूएचओ दिशानिर्देश का समर्थन करते हैं।

वित्त पोषण: विश्व स्वास्थ्य संगठन (पूर्व-आक्रामक गर्भाशय ग्रीवा रोग के स्क्रीनिंग और उपचार पर दिशानिर्देशों के लिए कार्य निष्पादन समझौता); यूरोपीय आयोग के अनुसंधान और नवाचार के लिए होराइजन 2020 फ्रेमवर्क ,

आरआईएससीसी नेटवर्क, संयुक्त कार्रवाई ईयूकेैनस्क्रीन और गर्भाशय ग्रीवा कैंसर पर यूरोपीय आयोग की पहल (ईसी-सीवीसी) के माध्यम से।
